# Chemoenzymatic Oxosulfonylation‐Bioreduction Sequence for the Stereoselective Synthesis of β‐Hydroxy Sulfones

**DOI:** 10.1002/cssc.202101313

**Published:** 2021-08-19

**Authors:** Marina López‐Agudo, Nicolás Ríos‐Lombardía, Javier González‐Sabín, Iván Lavandera, Vicente Gotor‐Fernández

**Affiliations:** ^1^ Organic and Inorganic Chemistry Department University of Oviedo Avenida Julián Clavería 8 Oviedo 33006 Spain; ^2^ EntreChem SL Vivero Ciencias de la Salud Santo Domingo de Guzmán Oviedo 33011 Spain

**Keywords:** alcohol dehydrogenases, biocatalysis, cascade reactions, hydroxy sulfones, oxosulfonylation

## Abstract

A series of optically active β‐hydroxy sulfones has been obtained through an oxosulfonylation‐stereoselective reduction sequence in aqueous medium. Firstly, β‐keto sulfones were synthesized from arylacetylenes and sodium sulfinates to subsequently develop the carbonyl reduction in a highly selective fashion using alcohol dehydrogenases as biocatalysts. Optimization of the chemical oxosulfonylation reaction was investigated, finding inexpensive iron(III) chloride hexahydrate (FeCl_3_ ⋅ 6H_2_O) as the catalyst of choice. The selection of isopropanol in the alcohol‐water media resulted in high compatibility with the enzymatic process for enzyme cofactor recycling purposes, providing a straightforward access to both (*R*)‐ and (*S*)‐β‐hydroxy sulfones. The practical usefulness of this transformation was illustrated by describing the synthesis of a chiral intermediate of Apremilast. Interestingly, the development of a chemoenzymatic cascade approach avoided the isolation of β‐keto sulfone intermediates, which allowed the preparation of chiral β‐hydroxy sulfones in high conversion values (83–94 %) and excellent optical purities (94 to >99 % *ee*).

## Graphical abstract

**Two for one**: The combination of iron(III) chloride and stereoselective alcohol dehydrogenases allows the development of an efficient oxosulfonylation‐bioreduction sequence to produce optically active β‐hydroxy sulfones from arylacetylenes and sodium sulfinates in aqueous medium.

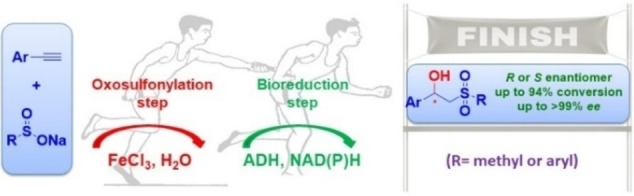

## Introduction

**Scheme 1:**
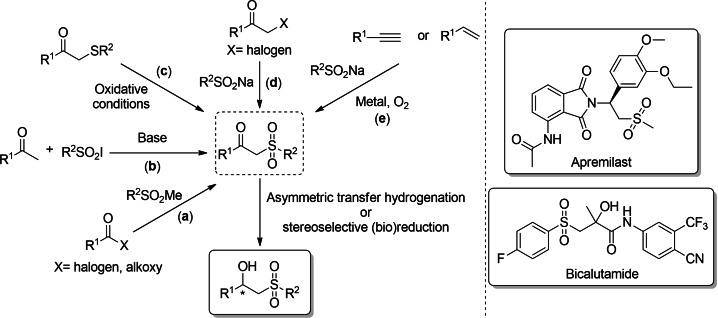
General synthetic approaches towards β‐keto sulfones, and their transformation into optically active β‐hydroxy sulfones. On the right, examples of biologically active molecules containing or derived from β‐hydroxy sulfones.

β‐Keto sulfones, also known as 2‐oxo‐sulfones, are privileged motifs in organic chemistry due to their synthetic versatility to produce a wide family of sulfur compounds.^[1]^ Their straightforward synthesis can be accomplished through different chemical routes, probably the most useful ones being: (a) the acylation of methyl sulfones; (b) reaction between generated enolates with sulfonyl iodides; (c) oxidation of β‐keto sulfides; and the reaction of sulfinate salts with (d) α‐halo ketones or (e) either alkynes or alkenes under aerobic conditions and metal‐ or photo‐catalysis (Scheme 1). In this context, the oxosulfonylation of multiple C−C bonds has received special attention due to its high atom‐economy, simple set‐up, and possibility to carry out a difunctionalization of alkynes in both aqueous and organic media.^[2]^ The presence of the sulfone moiety, α‐acidic protons at the methylene group, and the carbonyl function provide β‐keto sulfones with multiple synthetic possibilities, the asymmetric reduction of the carbonyl group representing the simple manner to provide access to optically active β‐hydroxy sulfones. These stereoselective processes have been successfully achieved using borane reductions,^[3]^ metal‐catalyzed transfer hydrogenations,^[4]^ or enzymatic transformations using carbonyl reductases.^[5]^ In fact, β‐hydroxy sulfones are highly attractive hydroxy‐functionalized compounds with remarkable biological profiles such as in the case of Bicalutamide (Casodex®), an antiandrogen medication for the treatment of prostate cancer, and useful chiral synthetic intermediates, for instance towards Apremilast (Otezla®), a drug employed in the treatment of psoriasis and psoriatic arthritis (Scheme 1).

**Scheme 2:**
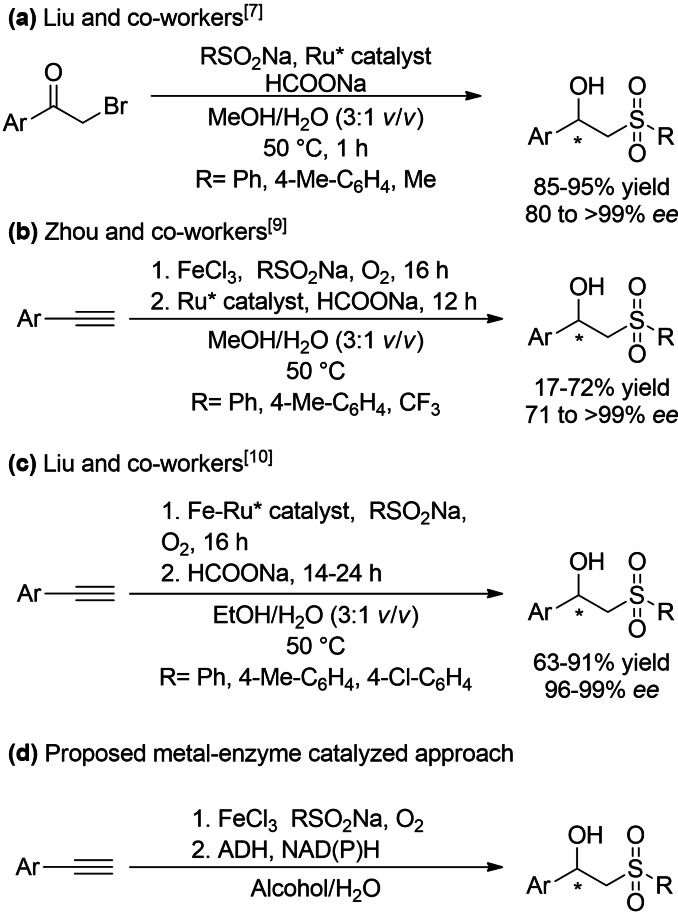
Multicatalytic approaches for the asymmetric synthesis of β‐hydroxy sulfones: (a) one‐pot nucleophilic substitution of α‐bromo ketones with sodium sulfinates followed by metal‐catalyzed ATH; (b, c) oxosulfonylation‐ATH sequences with two metal catalysts or in co‐immobilized form, respectively; (d) oxosulfonylation‐stereoselective bioreduction sequence proposed in this contribution.

Nowadays, multicatalytic transformations are particularly appealing, in order to gradually replace traditional synthetic stepwise sequences by cascade strategies.^[6]^ Thus, the straightforward construction of valuable compounds from accessible low‐cost raw materials and environmentally friendly catalysts becomes plausible, presenting many benefits in terms of economy, energy, waste minimization, and product yield. In this context, the synthesis of β‐hydroxy sulfones has been described in the last years by combining conventional chemical transformations with metal‐catalyzed asymmetric transfer hydrogenations (ATHs). For instance, Liu and co‐workers described a one‐pot process based on the nucleophilic substitution of α‐bromo ketones with sodium sulfinates and subsequent ruthenium‐catalyzed ATH using sodium formate as hydride source (Scheme 2a).^[7]^ Later on, the same research group reported a similar one‐pot approach using a supported chiral ruthenium complex, which was efficiently recycled for at least six cycles without significant decrease of the conversion and selectivity.^[8]^

Zhou and co‐workers also took advantage of the stereoselective action of a ruthenium complex for the reductive step, starting in this case with an oxosulfonylation reaction between terminal alkynes and sodium sulfinates under aerobic conditions. The resulting β‐hydroxy sulfones were obtained in moderate to good yields and good to excellent selectivities through a sequential one‐pot approach (Scheme 2b).^[9]^ More recently, Liu and co‐workers have described the co‐immobilization of iron(III) chloride and the ruthenium complex in mesoporous silica to develop the oxosulfonylation–ATH sequence, although sodium formate was added once the oxosulfonylation was finished (Scheme 2c). Interestingly, the reusability of the hybrid catalyst was feasible along several cycles without significant activity and selectivity loss.^[10]^ Remarkably, an alcohol‐water medium (3 : 1 *v*/*v*, methanol or ethanol) was employed in all these asymmetric metal‐catalyzed transformations. Until now, the reported syntheses of chiral β‐hydroxy sulfones are limited by the use of a unique ruthenium catalyst, therefore providing access in all cases to the same product enantiomer.

The combined use of metals and enzymes illustrates practical and elegant matches to increase molecular complexity, at the same time that the generation of chiral stereocenters can be accomplished.^[11]^ In this context, iron plays a pivotal role in organic chemistry,^[12]^ catalyzing, for instance, a myriad of redox transformations. At the same time, its abundance in Earth, low cost, and environmental profile in comparison with other metals^[13]^ make highly advisable the use of iron species with synthetic purposes. Therefore, encouraged by the symbiosis between iron catalysis and enzymes,^[14]^ and their compatibility in aqueous medium,^[15]^ the design of an oxosulfonylation‐stereoselective bioreduction sequence is herein attempted, searching for the production of both β‐hydroxy sulfone enantiomers (Scheme 2d). With the aim to prepare them, the use of alcohol dehydrogenases (ADHs) with opposite stereopreference will be investigated, trying to combine the exquisite chemoselectivity of metal catalysis, in this case an iron salt, with the enantiodiscrimination displayed by enzymes, which is an ideal solution for stereodivergent synthesis.

## Results and Discussion

The synthesis of 1‐phenyl‐2‐(phenylsulfonyl)ethan‐1‐one (**3** **a**) was selected as model reaction due to the commercial availability of phenylacetylene (**1** **a**) and sodium benzenesulfinate (**2** **a**), which reacted under aerobic conditions in the presence of inexpensive FeCl_3_ ⋅ 6H_2_O, to design an efficient oxosulfonylation reaction. The selection of this procedure was based on its capacity to be carried out in aqueous medium, which would be of interest to later develop the stereoselective cascade process previously depicted in Scheme 2d. The use of an alcohol‐aqueous medium turned out to be critical since the reaction did not proceed at any extension in neat water at 50 °C for 24 h. Such aqueous media were already explored in the literature for cascade β‐hydroxy sulfone syntheses,[^7^, ^8^, ^9^, ^10^] but in our case, the implementation of propan‐2‐ol (isopropanol, 2‐PrOH) as the co‐solvent instead of methanol or ethanol was aimed for cofactor recycling purposes in the bioreduction step. A comprehensive study is disclosed in Table 1, considering different optimization parameters that affect this reaction such as the temperature, phenylacetylene concentration, sulfinate molar excess, reaction medium composition, and catalyst loading.

**Table 1:** Optimization of the oxosulfonylation reaction between **1** **a** and **2** **a**.^[a]^

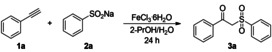						
						
Entry	FeCl_3_ ⋅ 6 H_2_O [mol %]	[**1** **a**] [mm]	**2** **a** [equiv.]	2‐PrOH/H_2_O [*v*/*v*]	*T* [°C]	Conv.^[b]^ [%]
1	20	100	1.5	3 : 1	RT	<3
2	20	100	1.5	3 : 1	50	38
3	20	100	1.5	3 : 1	80	55
4	20	50	1.5	3 : 1	80	38
5	20	200	1.5	3 : 1	80	74
6	20	300	1.5	3 : 1	80	61
7	20	200	1.5	2 : 1	80	77
8	20	200	1.5	1 : 1	80	84
9	20	200	1.5	1 : 2	80	76
10	20	200	1.2	1 : 1	80	77
11	20	200	2.0	1 : 1	80	87
12	10	200	1.5	1 : 1	80	34
13	25	200	1.5	1 : 1	80	96
14	30	200	1.5	1 : 1	80	99

[a] Sodium benzenesulfinate (**2** **a**, 1.2–2.0 equiv.) and FeCl_3_ ⋅ 6H_2_O (10–30 mol%) were added over a solution of phenylacetylene (**1** **a**, 0.18–1.05 mmol) in a 2‐PrOH/H_2_O mixture (3.5 mL), and it was stirred in the range between room temperature and 80 °C for 24 h under aerobic conditions. After this time, the reaction was quenched and the product recovered as described in the Experimental Section. [b] Conversion values were measured by HPLC.

The oxosulfonylation reaction of **1** **a** was firstly attempted at different temperatures ranging from room temperature, which led to the recovery of the unaltered starting materials (entry 1), to 50 °C (38 % conversion, entry 2) or 80 °C (55 % conversion, entry 3), the highest temperature providing the best result. Next, various acetylene concentrations were tested (**1** **a**, 50–300 mm, entries 4–6), obtaining the best conversion at 200 mm (74 %, entry 5). In comparison with the reaction described by Zhou and co‐workers using identical conditions (50 mm) but methanol as co‐solvent (93 %),^[9]^ a lower reactivity was observed (38 %). A minor influence was found when employing different 2‐PrOH/water ratios (3 : 1 to 1 : 2, entries 5 and 7–9), although a remarkable 84 % of **3** **a** was attained with the 1 : 1 mixture (entry 8). The amount of sodium benzenesulfinate was also investigated (entries 8, 10, and 11), reaching a slight improvement with 2 equivalents of the salt (87 % conversion, entry 11), although for atom efficiency and waste minimization, 1.5 equivalents of **2** **a** were selected for a final study in terms of catalyst loading (10–30 mol%, entries 8 and 12–14). Satisfyingly, the desired β‐keto sulfone **3** **a** was attained in over 95 % conversion for both 25 and 30 mol% of FeCl_3_ ⋅ 6H_2_O (entries 13 and 14).

Alternatively, other salts (as hexahydrate form) such as CuCl_2_, CoCl_2_, and NiCl_2_ did not provide better results, only finding conversion for copper(II) chloride (41 % at 20 mol%). In spite of the high FeCl_3_ catalyst loading for a catalytic process, it must be pointed out that this iron salt is a low‐cost reagent presenting very minor toxicity issues with regards to other metallic complexes and salts.[^13^, ^16^]

Finally, the reaction was scaled up at 2 mmol of **1** **a** under the conditions described in entry 8, producing highly pure **3** **a** in 76 % isolated yield after liquid‐liquid extraction. It is worth mentioning that further purification through column chromatography led to a significant yield loss (61 %), highlighting the present chemoenzymatic cascade towards optically active β‐hydroxy sulfone **4** **a** as a valuable synthetic approach to tackle the challenging and low‐yielding purification of the ketone intermediate.

**Scheme 3:**
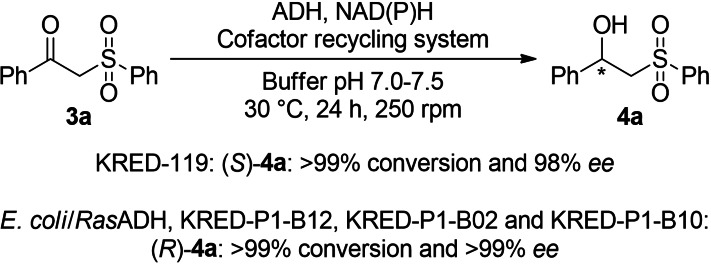
Bioreduction of β‐keto sulfone **3** **a** using ADHs.

Next, the bioreduction of **3** **a** (7 mm) was studied under standard conditions at 30 °C for 24 h (Scheme 3), using seven made in‐house ADHs heterologously expressed in *E. coli* from *Ralstonia* species (*Ras*ADH),^[17]^ *Sphingobium yanoikuyae* (*Sy*ADH),^[18]^ *Thermonoaerobacter* species (ADH‐T),^[19]^ *Thermonoaerobacter ethanolicus* (*TeS*ADH),^[20]^ *Rhodococcus ruber* (ADH‐A),^[21]^ *Lactobacillus brevis* (*Lb*ADH),^[22]^ and *Lactobacillus kefir* (*Lk*ADH),^[23]^ plus 20 commercial ketoreductases from Codexis Inc. (Codex® KRED Screening Kit) or Evoxx Technologies (evo.1.1.200). In each case, the best reported cofactor recycling system was employed. Extensive screening results and reaction conditions can be found in the Supporting Information (Table S1), finding quantitative conversions towards the formation of both 1‐phenyl‐2‐(phenylsulfonyl)ethan‐1‐ol (**4** **a**) enantiomers depending on the enzyme of choice. Remarkably, KRED‐119 provided access towards the (*S*)‐enantiomer in 98 % *ee*, while *Ras*ADH, KRED‐P1‐B12, KRED‐P1‐B02, and KRED‐P1‐B10 resulted as the best enzymes for the selective production of the (*R*)‐antipode.

In order to approach a manufacture setting, biotransformations at higher substrate concentrations (50 mm, Table 2, entries 1, 3, and 5–7) were also tested, leading in every case to quantitative conversions with excellent selectivities towards the production of enantiopure **4** **a**. In spite of also reaching a good productivity, further increase to 100 mm concentration revealed that KRED‐119 notably decreased the conversion (entries 1 and 2), while KRED‐P1‐B12 remained very selective towards (*R*)‐**4** **a** with excellent activity (entries 3 and 4).

**Table 2:** Bioreduction of **3** **a** at different concentrations with selected ADHs.^[a]^

Entry	ADH	[**3** **a**] [mM]	Conv.^[b]^ [%]	*ee* ^[b]^ [%]
1	KRED‐119	50	>99	>99 (*S*)
2	KRED‐119	100	75	>99 (*S*)
3	KRED‐P1‐B12	50	>99	>99 (*R*)
4	KRED‐P1‐B12	100	>99	>99 (*R*)
5	KRED‐P1‐B02	50	>99	>99 (*R*)
6	KRED‐P1‐B10	50	>99	>99 (*R*)
7	*Ras*ADH	50	>99	>99 (*R*)

[a] **Bioreduction with KRED‐119**: KRED‐119 (1 mg), β‐keto sulfone **3** **a** (50–100 mm), DMSO (1.8 % *v*/*v*), and 2‐PrOH (17.1 % *v*/*v*) were added to a 1.5 mL Eppendorf tube containing the mix‐N. **Bioreduction with KRED‐P1‐B12, KRED‐P1‐B02 and KRED‐P1‐B10**: the corresponding KRED (1 mg), β‐keto sulfone **3** **a** (50–100 mm), DMSO (1.8 % *v*/*v*), and 2‐PrOH (17.1 % *v*/*v*) were added to a 1.5 mL Eppendorf tube containing the mix‐P. **Bioreduction with** ***E. coli/Ras*** **ADH**: lyophilized cells of *Ras*ADH heterologously expressed in *E. coli* (15 mg), β‐keto sulfone **3** **a** (50 mm), DMSO (2.5 % *v*/*v*), an aqueous solution of d‐glucose (50 mm, 10 % *v*/*v*), and GDH‐105 (10 U, 11.7 % *v*/*v*) were added to a 1.5 mL Eppendorf tube with Tris⋅HCl buffer pH 7.5 (50 mm) and NADPH (1 mm). The reaction was shaken at 250 rpm and 30 °C for 24 h, analyzing the reaction outcome as described in the Experimental Section. [b] Conversion and enantiomeric excess values were measured by HPLC. The obtained β‐hydroxy sulfone **4** **a** enantiomer appears in parentheses.

**Scheme 4:**
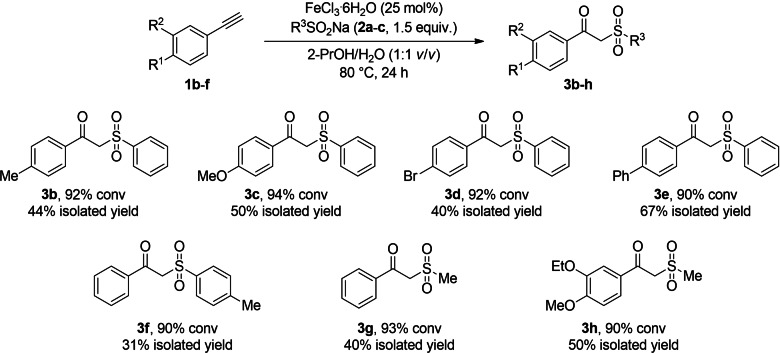
Synthesis of β‐keto sulfones **3** **b**–**h**.

At this point, the substrate scope was extended to a series of arylacetylenes (**1** **b**–**f**) and sodium sulfinates (**2** **a**–**c**), looking for the development of a general strategy towards the synthesis of optically active β‐hydroxy sulfones **4** **b**–**h**. For this selection (Scheme 4), a search of commercially available acetylene derivatives was made, finding **1** **b**–**e** as suitable candidates, as well as sodium sulfinates bearing a phenyl (**2** **a**), a 4‐methylphenyl (**2** **b**), or a methyl (**2** **c**) substitution. Remarkably, the synthesis of β‐keto sulfone **3** **h** was also considered, since it has been described as a suitable intermediate of Apremilast (Scheme 1), a medication employed in the treatment of certain types of psoriatic diseases.^[24]^

Prior to the assessment of the chemoenzymatic cascade, the β‐keto sulfone intermediates **3** **b**–**h** were purified trying to identify the optimal ADHs for the bioreduction process. In spite of the very high conversion values (90–94 %), the ketones were obtained in low to moderate yield after liquid‐liquid extraction and column chromatography purification (31–67 %). This fact stresses the importance of avoiding the isolation/purification of the transiently formed carbonyl species due to their instability, this step being avoided in the cascade approach.

Pleasingly, highly stereoselective and complementary ADHs were identified for most of the β‐keto sulfones **3** **b**–**h**. Although a comprehensive study can be found in the Supporting Information (Tables S2–S8), the best results in terms of enzyme activity and selectivity have been included in Table 3.

**Table 3:** Selected results in the bioreduction of β‐keto sulfones **3** **b**–**h**.^[a]^

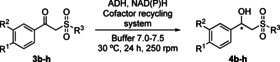				
				
Entry	Ketone **3** **b**–**h**	ADH	**4** **b**–**h** ^[b]^ [%]	*ee* ^[b]^ [%]
1	**3** **b**	KRED‐119	>99	>99 (*S*)
2	(R^1^=Me, R^2^=H, R^3^=Ph)	RasADH	86	>99 (*R*)
3		KRED‐130	>99	>99 (*R*)
4		KRED‐P1‐B02	>99	>99 (*R*)
5	**3** **c**	KRED‐119	>99	92 (*S*)
6	(R^1^=OMe, R^2^=H, R^3^=Ph)	KRED‐P1‐B05	94	>99 (*S*)
7		RasADH	95	>99 (*R*)
8	**3** **d**	KRED‐119	>99	>99 (*S*)
9	(R^1^=Br, R^2^=H, R^3^=Ph)	KRED‐P1‐B05	>99	>99 (*S*)
10		RasADH	91	>99 (*R*)
11		KRED‐130	97	>99 (*R*)
12		KRED‐P1‐B02	94	>99 (*R*)
13	**3** **e**	KRED‐119	92	>99 (*S*)
14	(R^1^=Ph, R^2^=H, R^3^=Ph)	KRED‐P1‐B05	89	>99 (*S*)
15		RasADH	60	>99 (*R*)
16		KRED‐130	55	>99 (*R*)
17	**3** **f**	KRED‐119	>99	93 (*S*)
18	(R^1^=R^2^=H, R^3^=4‐Me–C_6_H_4_)	RasADH	97	95 (*R*)
19		KRED‐P1‐B02	>99	>99 (*R*)
20		KRED‐P1‐B10	>99	>99 (*R*)
21	**3** **g**	KRED‐119	>99	>99 (*S*)
22	(R^1^=R^2^=H, R^3^=Me)	KRED‐P1‐B05	>99	>99 (*S*)
23		KRED‐P1‐B02	>99	>99 (*R*)
24		RasADH	>99	98 (*R*)
25		KRED‐P1‐B10	>99	>99 (*R*)
26	**3** **h**	KRED‐119	95	8 (*S*)
27	(R^1^=OMe, R^2^=OEt, R^3^=Me)	KRED‐P2‐D03	34	98 (*S*)
28		KRED‐P2‐D12	42	95 (*S*)
29		RasADH	93	80 (*R*)

[a] See the Experimental Section for additional details. [b] Conversion values and enantiomeric excess values were measured by HPLC. The major β‐hydroxy sulfone **4** **b**–**h** enantiomer appears in parentheses.

Remarkably, KRED‐119 allowed the access to the (*S*)‐β‐hydroxy sulfones with high activity and selectivity (92 to >99 % for both conversion and *ee*, entries 1, 5, 8, 13, 17, and 21), losing only the selectivity for the generation of (*S*)‐**4** **h** (8 % *ee*, entry 26). Conversely, other reductases such as KRED‐P2‐D03 and KRED‐P2‐D12 led to (*S*)‐**4** **h** in 98 and 95 % *ee* (entries 27 and 28), respectively, but with moderate conversions (34–42 %). Attempts to improve the conversion towards this alcohol were accomplished by testing DMSO as co‐solvent in different ratios (1.5–10 % *v*/*v*), different substrate concentrations (2.5–7 mm), and raising the temperature from 30 to 45 °C (Table S9). As a result of this parametrization, optimal results were found when using KRED‐P2‐D12 at 7 mm of substrate concentration, 30 °C, and the reaction medium supplemented with 10 % of DMSO, the target β‐hydroxy sulfone being recovered in 49 % conversion and 98 % *ee*. Continuing with the production of (*S*)‐β‐hydroxy sulfones, KRED‐P1‐B05 resulted to be a highly selective ketoreductase in many cases, rendering good to excellent conversions (entries 6, 9, 14, and 22). Regarding the production of the (*R*)‐enantiomers, it is worth mentioning that except for the formation of the alkoxy‐substituted (*R*)‐**4** **c** and (*R*)‐**4** **h**, or bulky (*R*)‐**4** **e**, suitable commercial ADHs such as KRED‐P1‐B02 were found to catalyze the bioreduction in very high to excellent conversions (94 to >99 %). In this context, and moving to the tested heterologously expressed enzymes on *E. coli*, *Ras*ADH provided the best results, favoring in all cases the formation of (*R*)‐**4** **b**–**h** with 60 to >99 % conversion and 80 to >99 % *ee* (entries 2, 7, 10, 15, 18, 24, and 29).

Next, the performance of the oxosulfonylation‐bioreduction sequence was explored, being the use of 2‐PrOH, instead of methanol, a crucial decision for the correct behavior of the selected ADHs. It must be mentioned that a sequential approach was devised due to the high temperature required for the oxosulfonylation reaction (80 °C), which is not compatible with the thermostability of the ADHs herein employed. For this reason, the concurrent cascade approach was discarded. According to the optimized conditions (see entry 13 in Table 1), which were alkyne **1** **a** (200 mm in a 2‐PrOH/H_2_O 1 : 1 *v*/*v* mixture), 1.5 equivalents of sulfinate **2** **a** and 25 mol% of FeCl_3_⋅6H_2_O at 80 °C, the oxosulfonylation was carried out for 24 h. Then the reaction mixture was cooled down, and two possibilities were studied: (a) take directly an aliquot (eighth of the total volume) from the reaction; or (b) centrifuge the mixture and take the same aliquot volume from the supernatant. Both aliquots were adjusted to a total volume corresponding to different substrate concentrations (10 or 50 mm for commercially available ADHs, and 25 mm for *Ras*ADH), and later subjected to the bioreduction process for 24 h at 30 °C and 250 rpm. Interestingly, the same results were achieved independently of the centrifugation step, so it was avoided for future experiments.

A comprehensive study regarding the development of this sequential approach has been addressed in the Supporting Information (Table S10), starting the oxosulfonylation reaction at 200 mm substrate concentration at 80 °C, and after 24 h the reaction mixture being alternatively diluted to 10, 25, or 50 mm substrate concentration, and the enzyme, NAD(P)H, and the nicotinamide cofactor recycling system subsequently added. The best results have been summarized in Table 4, where the highest ketone concentration was used to achieve the maximum productivity, maintaining excellent selectivity towards the formation of both β‐hydroxy sulfone **4** **a**–**g** enantiomers.

**Table 4:** Cascade oxosulfonylation‐bioreduction sequence between **1** **a**–**e** and **2** **a**–**c** for the production of enantioenriched **4** **a**–**g**.^[a]^

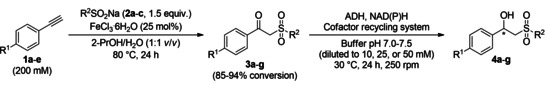							
							
Entry	R^1^	R^2^	**3** **a**–**g** ^[b]^	ADH	[**3** **a**–**g**] [mm]	**4** **a**–**g** ^[c]^ [%]	*ee* ^[d]^ [%]
1	H	Ph	**3** **a** (94 %)	KRED‐119	10	>99 (94)	>99 (*S*)
2				RasADH	25	>99 (94)	>99 (*R*)
3				KRED‐P1‐B02	50	>99 (94)	>99 (*R*)
4	Me	Ph	**3** **b** (92 %)	KRED‐119	10	>99 (92)	>99 (*S*)
5				RasADH	25	>99 (92)	>99 (*R*)
6				KRED‐P1‐B02	10	>99 (92)	>99 (*R*)
7	OMe	Ph	**3** **c** (94 %)	KRED‐119	10	>99 (94)	>99 (*S*)
8				RasADH	25	95 (89)	>99 (*R*)
9	Br	Ph	**3** **d** (92 %)	KRED‐119	50	>99 (92)	>99 (*S*)
10				KRED‐130	10	>99 (92)	>99 (*R*)
11	Ph	Ph	**3** **e** (90 %)	KRED‐119	10	92 (83)	>99 (*S*)
12				KRED‐130	50	>99 (90)	>99 (*R*)
13	H	4‐Me–C_6_H_4_	**3** **f** (85 %)	KRED‐119	50	>99 (85)	>99 (*S*)
14				KRED‐P1‐B02	50	>99 (85)	>99 (*R*)
15	H	Me	**3** **g** (88 %)	KRED‐119	50	>99 (88)	94 (*S*)
16				KRED‐P1‐B02	50	>99 (88)	>99 (*R*)

[a] Sodium sulfinate salt **2** **a**–**c** and FeCl_3_ ⋅ 6H_2_O were added to a solution of arylacetylene **1** **a**–**e** in a 2‐PrOH/H_2_O mixture, and it was stirred at 80 °C for 24 h under aerobic conditions. After this time, an aliquot was taken and added to another 1.5 mL Eppendorf tube. Subsequently, DMSO, 2‐PrOH, mix‐P (for KRED‐P1‐B02) or mix‐N (for KRED‐119 and KRED‐130), and the corresponding KRED were added. The reaction mixture with the required **3** **a‐**‐**g** concentration (10–50 mm) was shaken at 250 rpm and 30 °C for 24 h. For the reactions using *E. coli*/*Ras*ADH, after the oxosulfonylation reaction, DMSO, Tris⋅HCl buffer pH 7.5, NADPH, an aqueous solution of d‐glucose, GDH‐105, and the enzyme were added. [b] Conversion values of the oxosulfonylation step were measured by HPLC. [c] Conversion values of the bioreduction step were measured by HPLC. The overall conversion value for the oxosulfonylation‐bioreduction sequence appears in parentheses. [d] Enantiomeric excess values of the β‐hydroxy sulfones **4** **a**–**g** were measured by HPLC using a chiral column. The major enantiomer appears in parentheses.

Pleasingly, the sequential process proceeded smoothly since the ketone intermediates were obtained in high conversions (85–94 %), and these carbonyl intermediates were stereoselectively reduced using the previously identified enzyme candidates. In fact, in some cases a cooperative action of the metal and enzyme catalysts was observed, improving the final reaction conversion. All the alcohols **4** **a**–**g** were obtained in enantiomerically pure form, except methyl sulfone (*S*)‐**4** **g**, which was recovered with a remarkable 94 % *ee* (entry 15). The synthetic utility of this sequential transformation was demonstrated by setting up semipreparative reactions under optimized reaction conditions starting from 0.39 mmol of phenylacetylene. Thus, enantiopure (*R*)‐**4** **a** was recovered after liquid‐liquid extraction and column chromatography with commercial KRED‐P1‐B02 (66 % yield) and *Ras*ADH heterologously expressed in *E. coli* (53 % yield).

Finally, we performed a simplified environmental impact analysis making use of the E‐factor concept,^[25]^ and comparing it with the ones obtained for the most similar approaches[^7^, ^9^, ^10^] shown in Scheme 2 to synthesize β‐hydroxy sulfone **4** **a** (Section VI in the Supporting Information). Taking into the account the reagents, catalysts, and solvents employed in all these sequential protocols, we could confirm that our methodology presented a comparable value to the others (1.5‐fold higher than the one shown by Liu and co‐workers,^[10]^ 1.4‐fold higher than the method described by Zhou and co‐workers,^[9]^ and 1.2‐fold lower than the one shown by Liu and co‐workers^[7]^), with the advantage in our case that the most‐contributing factor, the solvent, is mainly composed of water, while in the other examples the medium is mostly organic. This shows that our chemoenzymatic method is competitive with other described chemical systems and demonstrates its high potential.

The assignment of the absolute configurations for the β‐hydroxy sulfones **4** **a**–**g** was established by comparison of their HPLC elution orders (Table S13) and optical rotation values (Table S14) with those reported in the literature, even though some discrepancies were found when analyzing the previously reported data. With this aim, the optical rotation values of optically active alcohols **4** **a**–**g** were measured after the performance of semipreparative bioreduction processes, and the assignment of their absolute configurations is next discussed.

The hydroxy sulfone **4** **a** obtained using *Ras*ADH led to similar optical rotation values as the ones obtained using complementary chemical methods such as the oxygenation of (*R*)‐1‐phenyl‐2‐phenylthioethan‐1‐ol^[26]^ or a (*R*)‐β‐hydroxy sulfoximine derivative,^[27]^ the asymmetric transfer hydrogenation of **3** **a** using (*S*,*S*)‐RuCl[*N*‐(tosyl)‐1,2‐diphenylethylenediamine](*p*‐cymene),[^4f^, ^28^] or the enantioselective conjugate boration of the corresponding α,β‐unsaturated sulfone using a (*R*,*S*)‐phosphine ligand.^[3d]^ In accordance, the opposite sign was found when (*S*)‐**4** **a** was obtained by enzymatic reduction with *Curvularia lunata*,^[5g]^ chemical reduction of **3** **a** using a chiral sulfonamide as ligand,^[3b]^ or by metal‐catalyzed asymmetric transfer hydrogenations of **3** **a** using a ruthenium(II) catalyst and a (*S*)‐diphenylphosphine ligand,^[4a]^ a cationic (*R*,*R*)‐ruthenium diamine catalyst,^[4g]^ or the combination of iridium and a chiral ferrocene‐based ligand.^[4i]^

The optical rotations obtained for other substrates such as **4** **b**–**d** and **4** **g** were also in accordance with those reported in the literature.[^4g^, ^4i^] All these works showed consistent results, only finding discrepancies in the literature since Zhou and co‐workers seem to have wrongly assigned the absolute configuration of **4** **a** when studying the Ru‐catalyzed asymmetric transfer hydrogenation of **3** **a** using a (*S*,*S*)‐ruthenium catalyst complex.^[9]^

On the other hand, the elution order of the chemoenzymatically obtained **4** **a** enantiomers using a Chiralcel AD−H column was compared with the one reported in the literature, finding a clear trend and appearing first the (*R*)‐enantiomer when using the same chiral stationary phase.[^4d^, ^4f^, ^4g^, ^4h^] Similarly, the enantiomer elution orders for **4** **b**[^4g^, ^4h^] and **4** **h** ^[24a]^ in the same column, and also for **4** **c** ^[4h]^ and **4** **d**[^4g^, ^4h^, ^4i^] in the Chiralcel OJ−H were in agreement with our previous assignment based on their optical rotation values. As pointed out before, it seems that an opposite elution order has been wrongly proposed by Liu and co‐workers and Zhou and co‐workers when using a ruthenium catalyst.[^7^, ^8^, ^9^]

## Conclusions

The combined use of metal and enzyme catalysis has been demonstrated this time for the development of a sequential oxosulfonylation‐bioreduction cascade transforming easily accessible acetylenes into highly valuable β‐hydroxy sulfones in optically active form. To achieve this aim, both steps have been individually analyzed. Firstly, the oxosulfonylation reaction of a series of arylacetylenes, bearing different substitutions at the C‐3 and C‐4 positions, in the presence of sodium alkyl and aryl sulfinates has been optimized by studying different parameters that affected to this catalytic system. The FeCl_3_ loading, substrate concentration, sulfinate molar excess, and temperature are key parameters in order to achieve very high conversions. This process has been performed in an aqueous‐alcoholic medium, replacing the traditional use of methanol by isopropanol, which simplifies the coupling of the bioreduction step as it provides the hydrogen donor for cofactor recycling purposes.

Exhaustive biocatalyst screening was made to identify active and stereoselective alcohol dehydrogenases, providing access to the desired β‐hydroxy sulfones with excellent conversion and selectivity. The sequential transformation was performed in two steps, firstly allowing the production of the β‐keto sulfone intermediates (85–94 % conversion), which were immediately diluted and transformed under ideal enzymatic conditions. Thus, β‐hydroxy sulfone enantiomers were obtained depending on the enzyme of choice with excellent enantioselectivity (94 to >99 %) and high to excellent global conversion values of the two consecutive processes (83–94 %).

## Experimental Section

### Materials

Codex® KRED Screening Kit and glucose dehydrogenase GDH‐105 (48 U mg^−1^ prepared as a stock solution of 3 mg in 1 mL of water) were purchased from Codexis Inc. KRED recycle mix‐P contains 128 mm sodium phosphate, 1.7 mm magnesium sulfate, and 1.1 mm NADP^+^, pH 7.0, while mix‐N contains 263 mm sodium phosphate, 1.7 mm magnesium sulfate, 1.1 mm NADP^+^, 1.1 mm NAD^+^, 80 mm d‐glucose, and 4.3 U mL^−1^ GDH, pH 7.0. Lyophilized *E. coli*/*Ras*ADH cells were obtained as previously described in the bibliography, and their activity was approximately 0.3 U mg^−1^ for the bioreduction of its model substrate (propiophenone).^[17b]^ Nicotinamide cofactors NADH and NADPH were acquired from Sigma Aldrich. Information from other ADHs can be found in the Supporting Information (Section I).

### General procedures

**Oxosulfonylation reaction**: Arylacetylene **1** **a**–**f** (0.7 mmol) was dissolved in a 2‐PrOH/H_2_O mixture (3.5 mL, 1 : 1 *v*/*v*) in a 25 mL round‐bottom flask. Then, sodium sulfinate **2** **a**–**c** (1.05 mmol) and FeCl_3_ ⋅ 6H_2_O (25 mol%) were added and the mixture stirred at 80 °C for 24 h under aerobic conditions. An aliquot was taken (5 μL) and dissolved in MeOH (200 μL) to determine the conversion by reverse‐phase HPLC. After this time, the reaction was quenched by the addition of H_2_O (5.0 mL), and the mixture extracted with ethyl acetate (3×10 mL). The combined organic layers were dried over Na_2_SO_4_, filtered. and evaporated under reduced pressure. The residue was purified by silica gel column chromatography to give the corresponding β‐keto sulfone **3** **a**–**h** (31–67 % yield, Scheme 4). The identities of these products were confirmed by the performance of NMR analyses and comparison with the reported spectra in the literature; data can be found in the Supporting Information (Section II.2).

**Bioreduction with ADHs from Codex® KRED Screening Kit**: The corresponding KRED (1 mg), β‐keto sulfone **3** **a**–**h**, (7 mm, 555 μL total volume), DMSO (10 μL, 1.8 % *v*/*v*), and 2‐PrOH (95 μL, 17.1 % *v*/*v*) were added to a 1.5 mL Eppendorf tube containing the mix‐P (450 μL of a stock solution of 300 mg mix‐P per 10 mL of water). The reaction was shaken at 250 rpm and 30 °C for 24 h. After this time, the mixture was extracted with ethyl acetate (2×500 μL), and the organic layers were separated by centrifugation (90 s, 13000 rpm), combined, and finally dried over Na_2_SO_4_. Conversions were determined by reverse‐phase HPLC and enantiomeric excess values of the alcohols were measured by HPLC using different chiral columns after evaporating the solvent and dissolving the samples in EtOH (see Tables S1–S9 in the Supporting Information). In the case of KRED‐101, KRED‐119, KRED‐130, KRED‐NADH‐101, and KRED‐NADH‐110, the mix‐N (450 μL of a stock solution of 90 mg mix‐N per 3 mL of water) was used.

**Bioreduction with** ***E**. **coli*** **/*Ras*ADH**: Lyophilized cells of *Ras*ADH heterologously expressed in *E. coli* (15 mg), β‐keto sulfone **3** **a**–**h** (25 mm, 600 μL total volume), DMSO (15 μL, 2.5 % *v*/*v*), an aqueous solution of D‐glucose (50 mm, 60 μL, 10 % *v*/*v*) and GDH‐105 (10 U, 70 μL, 11.7 % *v*/*v*) were added to a 1.5 mL Eppendorf tube with Tris⋅HCl buffer pH 7.5 (50 mm, 395 μL) and NADPH (1 mm, 60 μL of a 10 mm NADPH water solution). The reaction was shaken at 250 rpm and 30 °C for 24 h. After this time, the mixture was extracted with ethyl acetate (2×500 μL), and the organic layers were separated by centrifugation (90 s, 13000 rpm), combined, and finally dried over Na_2_SO_4_. Conversion and enantiomeric excess values were determined as already described for commercially available enzymes.

**Oxosulfonylation–bioreduction process in a sequential mode using ADHs from Codex® KRED Screening Kit**: Arylacetylene **1** **a**–**e** (0.7 mmol) was dissolved in a 2‐PrOH/H_2_O mixture (3.5 mL, 1 : 1 *v*/*v*) in a 25 mL round‐bottom flask. Then, the corresponding sodium sulfinate salt **2** **a**–**c** (1.05 mmol) and FeCl_3_ ⋅ 6H_2_O (25 mol%) were added and the mixture was stirred at 80 °C for 24 h under aerobic conditions. After this time, an aliquot (0.004 mmol, 20 μL) was taken and added to another 1.5 mL Eppendorf tube. Subsequently, DMSO (16 μL, 4 % *v*/*v*), 2‐PrOH (60 μL, 15 % *v*/*v*), mix‐P (304 μL for KRED‐P1‐B02 and KRED‐P1‐B05) or mix‐N (304 μL for KRED‐119 and KRED‐130), and the corresponding KRED (1 mg) were added. The reaction mixture with a final **3** **a**–**g** concentration of approximately 10 mm was shaken at 250 rpm and 30 °C for 24 h. Finally, the mixture was extracted with ethyl acetate (2×500 μL), and the organic layers were separated by centrifugation (90 s, 13000 rpm), combined, and finally dried over Na_2_SO_4_. Conversion and enantiomeric excess values were determined by HPLC as previously stated for the individual oxosulfonylation and bioreduction reactions.

**Oxosulfonylation‐bioreduction process in a sequential mode using** ***E**. **coli*** **/*Ras*ADH**: Arylacetylene **1** **a** or **1** **b** (0.67 mmol) was dissolved in a 2‐PrOH/H_2_O mixture (3.36 mL, 1 : 1 *v*/*v*) in a 25 mL round‐bottom flask. Then, sodium benzenesulfinate (**2** **a**, 165 mg, 1.01 mmol) and FeCl_3_ ⋅ 6H_2_O (36 mg, 25 mol%) were added and the mixture was stirred at 80 °C for 24 h under aerobic conditions. After this time, an aliquot (0.015 mmol, 75 μL) was taken and added to another 1.5 mL Eppendorf tube. Subsequently, DMSO (15 μL, 2.5 % *v*/*v*), Tris⋅HCl buffer pH 7.5 (50 mm, 320 μL), NADPH (1 mm, 60 μL of a 10 mm NADPH water solution), an aqueous solution of D‐glucose (50 mm, 60 μL, 10 % *v*/*v*), GDH‐105 (10 U, 70 μL, 11.7 % *v*/*v*), and *E. coli*/*Ras*ADH (15 mg) were added. The reaction mixture with a final **3** **a** or **3** **b** concentration of approximately 25 mm was shaken at 250 rpm and 30 °C for 24 h. Finally, the mixture was extracted with ethyl acetate (2×500 μL), and the organic layers were separated by centrifugation (90 s, 13000 rpm), combined, and finally dried over Na_2_SO_4_. Conversion and enantiomeric excess values of β‐hydroxy sulfone **4** **a** or **4** **b** were determined by HPLC as previously stated for the individual oxosulfonylation and bioreduction reactions.

## Conflict of interest

The authors declare no conflict of interest.

## Supporting information

As a service to our authors and readers, this journal provides supporting information supplied by the authors. Such materials are peer reviewed and may be re‐organized for online delivery, but are not copy‐edited or typeset. Technical support issues arising from supporting information (other than missing files) should be addressed to the authors.

Supporting Information

## References

[1a] Y. M. Markitanov , V. M. Timoshenko , Y. G. Shermolovich , J. Sulfur Chem. 2014, 35, 188–236;

[1b] J. Pena , R. F. Moro , I. S. Marcos , D. Díez , Curr. Org. Chem. 2014, 18, 2972–3036;

[1c] K. M. Elattar , A. Fekri , N. M. Bayoumy , A. A. Fadda , Res. Chem. Intermed. 2017, 43, 4227–4264;

[1d] R. J. Reddy , A. H. Kumari , J. J. Kumar , Org. Biomol. Chem. 2021, 19, 3087–3118. 33885563 10.1039/d1ob00111f

[2] S. Ghosh , S. Samanta , A. K. Ghosh , S. Neogi , A. Hajra , Adv. Synth. Catal. 2020, 362, 4552–4578.

[3] For representative examples:

[3a] B. T. Cho , D. J. Kim , Tetrahedron: Asymmetry 2001, 12, 2043–2047;

[3b] G. Zhao , J.-b. Hu , Z.-s. Qian , W.-x. Yin , Tetrahedron: Asymmetry 2002, 13, 2095–2098;

[3c] G.-y. Wang , X.-y. Liu , G. Zhao , Synlett 2006, 1150–1154;

[3d] A. L. Moure , R. Gómez Arrayás , J. C. Carretero , Chem. Commun. 2011, 47, 6701–6703. 10.1039/c1cc11949d 21584290

[4] For representative examples:

[4a] P. Bertus , P. Phansavath , V. Ratovelomanana-Vidal , J.-P. Genêt , A. R. Touati , T. Homri , B. B. Hassine , Tetrahedron Lett. 1999, 40, 3175–3178;

[4b] P. Bertus , P. Phansavath , V. Ratovelomanana-Vidal , J.-P. Genêt , A. R. Touati , T. Homri , B. B. Hassine , Tetrahedron: Asymmetry 1999, 10, 1369–1380;

[4c] H.-L. Zhang , X.-L. Hou , L.-X. Dai , Z.-B. Luo , Tetrahedron: Asymmetry 2007, 18, 224–228;

[4d] X. Wan , Q. Meng , H. Zhang , Y. Sun , W. Fan , Z. Zhang , Org. Lett. 2007, 9, 5613–5616; 18047366 10.1021/ol702565x

[4e] R. Touati , B. B. Hassine , Lett. Org. Chem. 2008, 5, 240–243;

[4f] Z. Ding , J. Yang , T. Wang , Z. Shen , Y. Zhang , Chem. Commun. 2009, 571–573; 10.1039/b818257d 19283294

[4g] X.-F. Huang , S.-Y. Zhang , Z.-C. Geng , C.-Y. Kwok , P. Liu , H.-Y. Li , X.-W. Wang , Adv. Synth. Catal. 2013, 355, 2860–2872;

[4h] V. K. Vyas , P. Srivastava , P. Bhatt , V. Shende , P. Ghosh , B. M. Bhanage , ACS Omega 2018, 3, 12737–12745; 31458000 10.1021/acsomega.8b01316 PMC6644779

[4i] L. Tao , C. Yin , X.-Q. Dong , X. Zhang , Org. Biomol. Chem. 2019, 17, 785–788. 30627711 10.1039/c8ob02923g

[5a] K. Nakamura , K. Ushio , S. Oka , A. Ohno , S. Yasui , Tetrahedron Lett. 1984, 36, 3979–3982;

[5b] R. Takinaga , K. Hosoya , A. Kaji , Chem. Lett. 1987, 16, 829–832;

[5c] R. Takinaga , K. Hosoya , A. Kaji , J. Chem. Soc. Perkin Trans. 1 1988, 1799–1803;

[5d] T. Sato , Y. Okumura , J. Itai , T. Fujisawa , Chem. Lett. 1988, 17, 1537–1540;

[5e] S. Robin , F. Huet , A. Fauve , H. Veschambre , Tetrahedron: Asymmetry 1993, 4, 239–246;

[5f] K. Lorraine , S. King , R. Greasham , M. Chartrain , Enzyme Microb. Technol. 1996, 19, 250–255; 8987484 10.1016/0141-0229(95)00242-1

[5g] V. Gotor , F. Rebolledo , R. Liz , Tetrahedron: Asymmetry 2001, 12, 513–515.

[6a] D. B. Ramachary , S. Jain , Org. Biomol. Chem. 2011, 9, 1277–1300; 21120241 10.1039/c0ob00611d

[6b] Ł. Albrecht , H. Jiang , K. A. Jørgensen , Angew. Chem. Int. Ed. 2011, 50, 8492–8509; 10.1002/anie.201102522 21826772

[6c] M. J. Climent , A. Corma , S. Iborra , M. J. Sabater , ACS Catal. 2014, 4, 870–891;

[6d] Y. Hayashi , Chem. Sci. 2016, 7, 866–880; 28791118 10.1039/c5sc02913a PMC5529999

[6e] F. R. Bisogno , M. G. López-Vidal , G. de Gonzalo , Adv. Synth. Catal. 2017, 359, 2026–2049;

[6f] J. H. Schrittwieser , S. Velikogne , M. Hall , W. Kroutil , Chem. Rev. 2018, 118, 270–348; 28481088 10.1021/acs.chemrev.7b00033

[6g] E. Heuson , F. Dumeignil , Catal. Sci. Technol. 2020, 10, 7082–7100.

[7] D. Zhang , T. Cheng , Q. Zhao , J. Xu , G. Liu , Org. Lett. 2014, 16, 5764–5767. 25341700 10.1021/ol502832a

[8] J. Wang , L. Wu , X. Hu , R. Jin , G. Liu , Catal. Sci. Technol. 2017, 7, 4444–4450.

[9] P. Cui , Q. Liu , J. Wang , H. Liu , H. Zhou , Green Chem. 2019, 21, 634–639.

[10] S. Wang , C. Wang , N. Lv , C. Tan , T. Cheng , G. Liu , ChemCatChem 2021, 13, 909–915.

[11a] C. A. Denard , J. F. Hartwig , H. Zhao , ACS Catal. 2013, 3, 2856–2864;

[11b] H. Gröger , W. Hummel , Curr. Opin. Chem. Biol. 2014, 19, 171–179; 24709123 10.1016/j.cbpa.2014.03.002

[11c] V. Köhler , N. J. Turner , Chem. Commun. 2015, 51, 450–464; 10.1039/c4cc07277d 25350691

[11d] F. Rudroff , M. D. Mihovilovic , H. Gröger , R. Snajdrova , H. Iding , U. T. Bornscheuer , Nat. Catal. 2018, 1, 12–22;

[11e] X. Huang , M. Cao , H. Zhao , Curr. Opin. Chem. Biol. 2020, 55, 161–170; 32179434 10.1016/j.cbpa.2020.02.004

[11f] Y. Liu , P. Liu , S. Gao , Z. Wang , P. Luan , J. González-Sabín , Y. Jiang , Chem. Eng. J. 2021, 420, 127659.

[12a] C. Bolm , J. Legros , J. Le Paih , L. Zani , Chem. Rev. 2004, 104, 6217–6254; 15584700 10.1021/cr040664h

[12b] I. Bauer , H.-J. Knölker , Chem. Rev. 2015, 115, 3170–3387; 25751710 10.1021/cr500425u

[12c] A. Fürstner , ACS Cent. Sci. 2016, 2, 778–789; 27981231 10.1021/acscentsci.6b00272 PMC5140022

[12d] A. Guðmundsson , J. E. Bäckvall , Molecules 2020, 25, 1349.

[13] K. S. Egorova , V. P. Ananikov , Angew. Chem. Int. Ed. 2016, 55, 12150–12162; 10.1002/anie.201603777 27532248

[14a] A. Quintard , Chem. Eur. J. 2021, 27, 89–105; 32490569 10.1002/chem.202002092

[14b] P. Kaur , V. Tyagi , Adv. Synth. Catal. 2021, 363, 877–905.

[15] N. Ríos-Lombardía , J. García-Álvarez , J. González-Sabín , Catalysts 2018, 8, 75.

[16] K. S. Egorova , V. P. Ananikov , Organometallics 2017, 36, 4071–4090.

[17a] I. Lavandera , A. Kern , B. Ferreira-Silva , A. Glieder , S. de Wildeman , W. Kroutil , J. Org. Chem. 2008, 73, 6003–6005; 18597534 10.1021/jo800849d

[17b] H. Man , K. Kędziora , J. Kulig , A. Frank , I. Lavandera , V. Gotor-Fernández , D. Rother , S. Hart , J. P. Turkenburg , G. Grogan , Top. Catal. 2014, 57, 356–365.

[18] I. Lavandera , A. Kern , V. Resch , B. Ferreira-Silva , A. Glieder , W. M. F. Fabian , S. de Wildeman , W. Kroutil , Org. Lett. 2008, 10, 2155–2158. 18459797 10.1021/ol800549f

[19] J. Peters , T. Minuth , M.-R. Kula , Enzyme Microb. Technol. 1993, 11, 950–958. 10.1016/0141-0229(93)90171-w 7764255

[20] C. Heiss , M. Laivenieks , J. G. Zeikus , R. S. Phillips , Bioorg. Med. Chem. 2001, 9, 1659–1666. 11425565 10.1016/s0968-0896(01)00073-6

[21] W. Stampfer , B. Kosjek , C. Moitzi , W. Kroutil , K. Faber , Angew. Chem. Int. Ed. 2002, 41, 1014–1017; 10.1002/1521-3773(20020315)41:6<1014::aid-anie1014>3.0.co;2-6 12491297

[22] S. Leuchs , L. Greiner , Chem. Biochem. Eng. Q. 2011, 25, 267–281.

[23a] C. W. Bradshaw , W. Hummel , C. H. Wong , J. Org. Chem. 1992, 57, 1532–1536;

[23b] A. Weckbecker , W. Hummel , Biocatal. Biotransform. 2006, 24, 380–389.

[24a] A. L. Ruchelman , T. J. Connolly , Tetrahedron: Asymmetry 2015, 26, 553–559;

[24b] K. B. Vega , D. M. V. Cruz , A. R. T. Oliveira , M. R. da Silva , T. L. G. de Lemos , M. C. F. Oliveira , R. D. S. Bernardo , J. R. de Sousa , G. Zanatta , F. D. Nasário , A. J. Marsaioli , M. C. de Mattos , J. Braz. Chem. Soc. 2021, 32, 1100–1110.

[25a] M. Eissen , J. O. Metzger , Chem. Eur. J. 2002, 8, 3580–3585; 12203284 10.1002/1521-3765(20020816)8:16<3580::AID-CHEM3580>3.0.CO;2-J

[25b] J. H. Schrittwieser , F. Coccia , S. Kara , B. Grischek , W. Kroutil , N. d'Alessandro , F. Hollmann , Green Chem. 2013, 15, 3318–3331;

[25c] EATOS: Environmental Assessment Tool for Organic Syntheses, http://www.metzger.chemie.uni-oldenburg.de/eatos/english.htm.

[26] M. Node , K. Nishide , Y. Shigeta , K. Obata , H. Shiraki , H. Kunishige , Tetrahedron 1997, 53, 12883–12894.

[27] J. Hachtel , H.-J. Gais , Eur. J. Org. Chem. 2000, 1457–1465.

[28] Z. Xu , S. Zhu , Y. Liu , L. He , Z. Geng , Y. Zhang , Synthesis 2010, 811–817.

