# Supplementary material for: Chemoenzymatic Oxosulfonylation‐Bioreduction Sequence for the Stereoselective Synthesis of β‐Hydroxy Sulfones
Source: ChemSusChem. 2021 Aug 19;15(9):e202101313. doi: 10.1002/cssc.202101313 (PMC9292901; doi:10.1002/cssc.202101313)
Supplement: Supplementary file 1 — Supporting Information [file CSSC-15-0-s001.pdf]

ChemSusChem

Supporting Information

Chemoenzymatic Oxosulfonylation-Bioreduction Sequence for the Stereoselective Synthesis of β-Hydroxy Sulfones

Marina López-Agudo, Nicolás Ríos-Lombardía, Javier González-Sabín,* Iván Lavandera, and Vicente Gotor-Fernández*This publication is part of a collection of invited contributions focusing on “Biocatalysis as Key to Sustainable Industrial Chemistry”. Please visit to view all contributions.© 2021 The Authors. ChemSusChem published by Wiley-VCH GmbH. This is an open access article under the terms of the Creative Commons Attribution License, which permits use, distribution and reproduction in any medium, provided the original work is properly cited.

Supporting Information (Page 1 out of 83)

I. General considerations S3

II. Chemical synthesis and product characterization S4

II.1. Preparation of 3-ethoxy-4-methoxyphenylacetylene (1f) S4

II.2. General procedure for the synthesis of β-keto sulfones 3a-h S5

II.3. General procedure for the reduction of β-keto sulfones 3a-h to produce racemic β-hydroxy sulfones 4a-h S7

III. General procedure for enzymatic reduction screenings S9

III.1. Bioreduction of 3a-h with KREDs from Codexis Inc S9

III.2. Bioreduction of 3a-h with *Ras*ADH S9

III.3. Bioreduction of 3a-h with *Sy*ADH, ADH-T, *TeS*ADH or ADH-A S9

III.4. Bioreduction of 3a-h with *Lb*ADH S10

III.5. Bioreduction of 3a-h with *Lk*ADH S10

III.6. Bioreduction of 3a-h with commercial evo-1.1.200 ADH S10

III.7. Results of the bioreduction screenings S11

IV. Study of the dilution in the sequential process S19

V. Semi-preparative scale-up for the oxosulfonylation-bioreduction sequence to produce (*R*)-4a S21

V.1. Sequential transformation using KRED-P1-B02 S21

V.2. Sequential transformation using *Ras*ADH S21

VI. EATOS calculations S22

VII. HPLC analyses for the determination of reaction conversion and product enantiomeric excess values S25

VII.1. HPLC analyses for determination of reaction conversion values S25

VII.2. HPLC analyses for determination of β-hydroxy sulfone enantiomeric excess values S26

VII.3. Calibration curves for conversion values determination S27

VII.3.1. Calibration curve for conversion of phenylacetylene (1a) into 1-phenyl-2-(phenylsulfonyl)ethan-1-one (3a) at 228 nm wavelength S27

VII.3.2. Calibration curve for conversion of phenylacetylene (1a) into 1-phenyl-2-tosylethan-1-one (3f) at 228 nm wavelength S27

VII.3.3. Calibration curve for conversion of phenylacetylene (1a) into 2-(methylsulfonyl)-1-phenylethan-1-one (3g) at 218 nm wavelength S28

VII.3.4. Calibration curve for conversion of 3-ethoxy-4-methoxyphenylacetylene (1f) into 1-(3-ethoxy-4-methoxyphenyl)-2-(methylsulfonyl)ethan-1-one (3h) at 228 nm wavelength S28

VII.3.5. Calibration curve for conversion of 1-phenyl-2-(phenylsulfonyl)ethan-1-one (3a) into 1-phenyl-2-(phenylsulfonyl)ethan-1-ol (4a) at 210 nm wavelength S29

VII.3.6. Calibration curve for conversion of 1-(4-methylphenyl)-2-(phenylsulfonyl) ethan-1-one (3b) into 1-(4-methylphenyl)-2-(phenylsulfonyl)ethan-1-ol (4b) at 210 nm wavelength S29

VII.3.7. Calibration curve for conversion of 1-(4-methoxyphenyl)-2-(phenylsulfonyl)ethan-1-one (3c) into 1-(4-methoxyphenyl)-2-(phenylsulfonyl) ethan-1-ol (4c) at 210 nm wavelength S30

VII.3.8. Calibration curve for conversion of 1-(4-bromophenyl)-2-(phenylsulfonyl) ethan-1-one (3d) into 1-(4-bromophenyl)-2-(phenylsulfonyl)ethan-1-ol (4d) at 210 nm wavelength S30

VII.3.9. Calibration curve for conversion of 1-[(1,1'-biphenyl)-4-yl] -2-(phenylsulfonyl)ethan-1-one (3e) into 1-[(1,1'-biphenyl)-4-yl] -2-(phenylsulfonyl)ethan-1-ol (4e) at 210 nm wavelength S31

VII.3.10. Calibration curve for conversion of 1-phenyl-2-tosylethan-1-one (3f) into 1-phenyl-2-tosylethan-1-ol (4f) at 210 nm wavelength S31

VII.3.11. Calibration curve for conversion of 2-(methylsulfonyl)-1-phenylethan--1-one (3g) into 2-(methylsulfonyl)-1-phenylethan-1-ol (4g) at 210 nm wavelength S32

VII.3.12. Calibration curve for conversion of into 1-(3-ethoxy-4-methoxyphenyl) -2-(methylsulfonyl)ethan-1-one (3h) into 1-(3-ethoxy-4-methoxyphenyl) -2-(methylsulfonyl)ethan-1-ol (4h) at 210 nm wavelength S32

VIII. HPLC chromatograms S33

VIII.1. Copy of HPLC chromatograms for racemic and enantioenriched 4a-h S33

VIII.2. HPLC analysis for semi-preparative sequential transformation of 1a into 4a S41

IX. Assignment of the absolute configuration for β-hydroxy sulfones 4a-h S42

X. References S43

XI. NMR spectra S44

I. General considerations

Codex® KRED Screening Kit and glucose dehydrogenase GDH-105 (44 U/mg) were purchased from Codexis Inc. The evo.1.1.200 was purchased from Evoxx Technologies. Nicotinamide cofactors NADH and NADPH were acquired from Sigma Aldrich. Lyophilized *E. coli*/*Ras*ADH cells were obtained as previously described in the bibliography, and their activity was approximately 0.3 U/mg for the bioreduction of its model substrate (propiophenone).[1] Other alcohol dehydrogenases have been obtained as described elsewhere: *Sphingobium yanoikuyae* (*Sy*ADH),[2] *Thermonoaerobacter* species (ADH-T),[3] *Thermonoaerobacter ethanolicus* (*TeS*ADH),[4] *Rhodococcus ruber* (ADH-A),[5] *Lactobacillus brevis* (*Lb*ADH)[6] and *Lactobacillus kefir* (*Lk*ADH, 1468 U/mL for acetophenone bioreduction)[7].

^{1}H, ^{13}C and DEPT NMR experiments were recorded on a Bruker AV 300 MHz spectrometer. All chemical shifts (δ) are given in parts per million (ppm). Optical rotations were measured using a Perkin-Elmer 241 polarimeter. IR spectra were recorded in a Jasco FT/IR-4700 spectrometer using pure neat samples. Melting points measurements were performed in open capillary tubes and are uncorrected. Thin-layer chromatography (TLC) analyses were conducted with Merck Silica Gel 60 F254 precoated plates and visualized with UV, and potassium permanganate stain. Column chromatographies were performed using silica gel 60 (230-240 mesh).

High-performance liquid chromatography (HPLC) analyses were carried out for the measurement of conversion and enantiomeric excess values. An Agilent RR1200 chromatograph with a reverse phase column (Zorbax Eclipse XDB-C18, RR, 18 μm, 4.6 x 50 mm, Agilent) was used for the measurement of conversion values of the oxosulfonylation and bioreduction steps (see Section VI.1). An Agilent 1260 Infinity chromatograph in combination with chiral columns was employed for the determination of the β-hydroxy sulfone enantiomeric excess values (see Section VI.2).

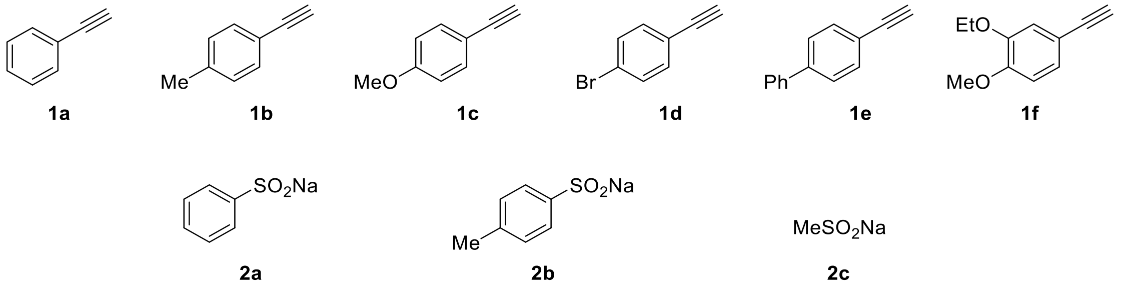

Figure S1. Starting materials employed in this contribution: Arylacetylenes **1a-f** and sodium sulfinates **2a-c**.

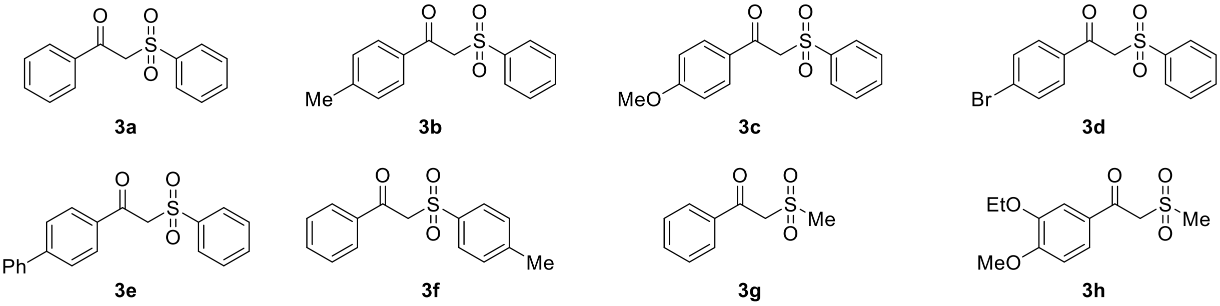

Figure S2. β-Keto sulfones **3a-h** described in this contribution.

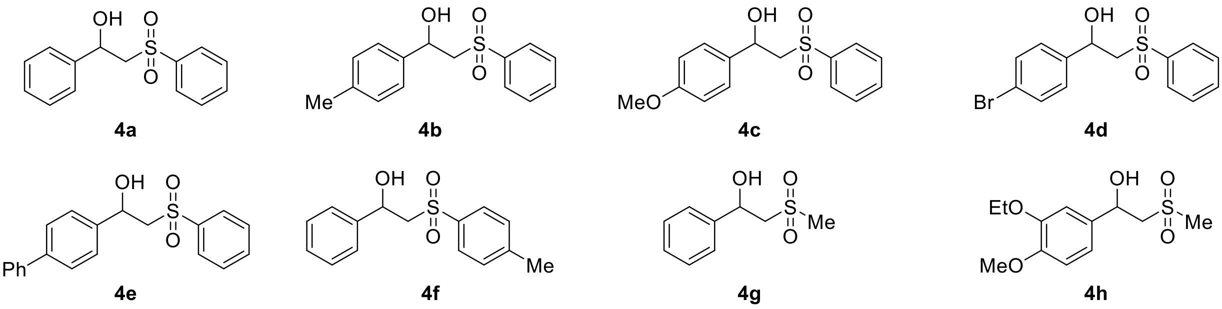

Figure S3. β-Hydroxy sulfones **4a-h** described in this contribution.

II. Chemical synthesis and product characterization

II.1. Preparation of 3-ethoxy-4-methoxyphenylacetylene (1f)[8]

A solution of triphenylphosphine (Ph_3P, 10.5 g, 40 mmol) in CH_2Cl_2 (10 mL) was added dropwise to a cooled (ice/water bath) and well-stirred solution of 3-ethoxy-4-methoxybenzaldehyde (1.8 g, 10 mmol) and carbon tetrabromide (CBr_4, 6.63 g, 20 mmol) in CH_2Cl_2 (10 mL). After stirring for 30 min, the solvent was removed under reduced pressure and the residue was purified by column chromatography on silica gel (Hex:EtOAc 9:1) to obtain a crude solid (2.16 g, approximately 6.5 mmol of the intermediate compound, 65% yield). Then, the crude reaction was dissolved in anhydrous THF (6 mL) under nitrogen atmosphere, and *n*-BuLi (5.4 mL, 13 mmol, 2.4 M in hexane) was added dropwise at –78 ºC. After 1 h, MeOH (5 mL) was added, and the mixture was stirred for additional 1 h. Finally, the reaction was quenched with a NH_4Cl saturated aqueous solution at 0 ºC and the aqueous phase was extracted with Et_2O (2 x 20 mL). The organic layers were combined, dried over Na_2SO_4, filtered and concentrated under reduced pressure to obtain alkyne **1f** as a yellow solid (1.11 g, 63% global yield). R_f (Hex:EtOAc, 9:1): 0.63. Yellowish solid. Mp: 95-97 ºC. IR (neat): ν 3259 (C≡CH),

2099, 1511, 1458, 1443, 1412, 1321, 1233, 1129, 1045, 1020 cm^{-1}. ^{1}H-NMR ($CDCl_3$, 300.13 MHz): δ 1.48 (t, *J* = 7.0 Hz, 3H), 3.01 (s, 1H), 3.89 (s, 3H), 4.10 (q, *J* = 7.0 Hz, 2H), 6.81 (d, *J* = 8.3 Hz, 1H), 7.00 (d, *J* = 1.9 Hz, 1H), 7.10 (dd, *J* = 8.3, 1.9 Hz, 1H). ^{13}C-NMR ($CDCl_3$, 75.5 MHz): δ 14.7 (CH_3), 55.9 (CH_3), 64.3 (CH_2), 75.6 (CH), 83.8 (C), 111.1 (CH), 114.1 (C), 116.0 (CH), 125.4 (CH), 147.9 (C), 150.1 (C).

II.2. General procedure for the synthesis of β-keto sulfones 3a-h

Arylacetylene **1a-f** (0.7 mmol) was dissolved in a 2-PrOH/H_2O mixture (3.5 mL, 1:1 v/v) in a 25 mL round bottom flask. Then, sodium sulfinate **2a-c** (1.05 mmol) and $FeCl_3 \cdot 6H_2O$ (25 mol%) were added and the mixture stirred at 80 ºC for 24 h under aerobic conditions. After this time, the reaction was quenched by the addition of H_2O (5.0 mL), and the mixture extracted with EtOAc (3 x 10 mL). The combined organic layers were dried over Na_2SO_4, filtered and evaporated under reduced pressure. The residue was purified by silica gel column chromatography to give the corresponding β-keto sulfone **3a-h** (31-67% yield).

1-Phenyl-2-(phenylsulfonyl)ethan-1-one (3a). 61% yield. Purified by column chromatography ($CHCl_3$). R_f (Hex:EtOAc, 2:1): 0.35. Orange solid. Mp: 94-96 ºC. IR (neat): ν 3061, 1678 (C=O), 1447, 1308 (SO_2), 1273, 1152 (SO_2), 684 cm^{-1}. ^{1}H-NMR ($CDCl_3$, 300.13 MHz): δ 4.76 (s, 2H), 7.46-7.70 (m, 6H), 7.93-7.96 (m, 4H). ^{13}C-NMR ($CDCl_3$, 75.5 MHz): δ 63.4 (CH_2), 128.6 (2CH), 128.9 (2CH), 129.2 (2CH), 129.3 (2CH), 134.3 (CH), 134.4 (CH), 135.7 (C), 138.7 (C), 188.0 (C).

1-(4-Methylphenyl)-2-(phenylsulfonyl)ethan-1-one (3b). 44% yield. Purified by column chromatography ($CHCl_3$). R_f (Hex:EtOAc, 2:1): 0.43. Orange solid. Mp: 120-123 ºC. IR (neat): ν 3005, 1667 (C=O), 1448, 1275 (SO_2), 1267, 1161 (SO_2), 766 cm^{-1}. ^{1}H-NMR ($CDCl_3$, 300.13 MHz): δ 2.44 (s, 3H), 4.73 (s, 2H), 7.29 (m, 2H), 7.53-7.59 (m, 2H), 7.68 (m, 1H), 7.84-7.93 (m, 4H). ^{13}C-NMR ($CDCl_3$, 75.5 MHz): δ 21.8 (CH_3), 63.4 (CH_2), 128.6 (2CH), 129.2 (2CH), 129.5 (2CH), 129.6 (2CH), 133.3 (C), 134.2 (CH), 138.8 (C), 145.7 (C), 187.5 (C).

1-(4-Methoxyphenyl)-2-(phenylsulfonyl)ethan-1-one (3c) 50% yield. Purified by column chromatography ($CHCl_3$). R_f (Hex:EtOAc, 2:1): 0.30. Yellowish solid. Mp: 110-113 ºC. IR (neat): ν 2994, 1663 (C=O), 1593, 1275 (SO_2), 1259, 1155 (SO_2), 751 cm^{-1}. ^{1}H-NMR ($CDCl_3$, 300.13 MHz): δ 3.89 (s, 3H), 4.71 (s, 2H), 6.94 (d, 2H, *J* = 9.0 Hz), 7.56 (m, 2H), 7.61-7.69 (m, 1H), 7.92 (t, 4H, *J* = 9.0 Hz). ^{13}C-NMR ($CDCl_3$, 75.5 MHz): δ 55.6 (CH_3), 63.4 (CH_2), 114.1 (2CH), 128.5 (2CH), 128.8 (C), 129.2 (2CH), 131.9 (2CH), 134.2 (CH), 138.8 (C), 164.6 (C), 186.2 (C).

1-(4-Bromophenyl)-2-(phenylsulfonyl)ethan-1-one (3d). 40% yield. Purified by column chromatography ($CHCl_3$). R_f (Hex:EtOAc, 2:1): 0.51. Yellow solid. Mp: 133-136 ºC. IR (neat): ν 3005, 1675 (C=O), 1583, 1275 (SO_2), 1261, 1069 (SO_2), 750 cm^{-1}. ^{1}H-NMR ($CDCl_3$, 300.13 MHz): δ 4.72 (s, 2H), 7.57-7.70 (m, 5H), 7.82-7.91 (m, 4H). ^{13}C-NMR ($CDCl_3$, 75.5 MHz): δ 63.5 (CH_2), 128.5 (2CH), 129.3 (2CH), 130.0 (C), 130.8 (2CH), 132.3 (2CH), 134.4 (CH), 138.5 (C), 138.5 (C), 187.1 (C).

1-[(1,1'-Biphenyl)-4-yl]-2-(phenylsulfonyl)ethan-1-one (3e). 67% yield. Purified by column chromatography ($CHCl_3$). R_f (Hex:EtOAc, 2:1): 0.40. Orange solid. Mp: 140-143 ºC. IR (neat): ν 3006, 1674 (C=O), 1448, 1275 (SO_2), 1261, 1147 (SO_2), 764 cm^{-1}. ^{1}H-NMR ($CDCl_3$, 300.13 MHz): δ 4.80 (s, 2H), 7.47-7.73 (m, 12H), 7.93-7.96 (m, 1H), 8.04 (d, J = 8.5 Hz, 1H). ^{13}C-NMR ($CDCl_3$, 75.5 MHz): δ 63.6 (CH_2), 127.1 (CH), 127.4 (2CH), 127.5 (2CH), 128.6 (2CH), 129.1 (2CH), 129.3 (2CH), 130.0 (2CH), 134.3 (CH), 134.4 (C), 138.7 (C), 139.4 (C), 147.1 (C), 187.5 (C).

1-Phenyl-2-tosylethan-1-one (3f). 31% yield. Purified by column chromatography ($CHCl_3$). R_f (Hex:EtOAc, 2:1): 0.44. Orange solid. Mp: 105-107 ºC. IR (neat): ν 2999, 1677 (C=O), 1447, 1270 (SO_2), 1186, 1147 (SO_2), 735 cm^{-1}. ^{1}H-NMR ($CDCl_3$, 300.13 MHz): δ 2.45 (s, 3H), 4.74 (s, 2H), 7.35 (d, J = 7.8 Hz, 2H), 7.49 (dd, J = 8.3, 7.0 Hz, 2H), 7.56-7.69 (m, 1H), 7.78 (d, J = 8.5 Hz, 2H), 7.96 (dd, J = 7.1, 1.8 Hz, 2H). ^{13}C-NMR ($CDCl_3$, 75.5 MHz): δ 21.7 (CH_3), 63.6 (CH_2), 128.6 (2CH), 128.8 (2CH), 129.3 (2CH), 129.8 (2CH), 134.3 (CH), 135.8 (2C), 145.4 (C), 188.2 (C).

2-(Methylsulfonyl)-1-phenylethan-1-one (3g). 40% yield. Purified by column chromatography ($CHCl_3$). R_f (Hex:EtOAc, 2:1): 0.29. Yellow solid. Mp: 105-107 ºC. IR (neat): ν 3028, 1674 (C=O), 1451, 1298 (SO_2), 1220, 1117 (SO_2), 688 cm^{-1}. ^{1}H-NMR ($CDCl_3$, 300.13 MHz): δ 3.15 (s, 3H), 4.63 (s, 2H), 7.53 (t, J = 7.4 Hz, 2H), 7.65 (m, 1H), 8.00 (dd, J = 8.5, 1.4 Hz, 2H). ^{13}C-NMR ($CDCl_3$, 75.5 MHz): δ 41.9 (CH_3), 61.2 (CH_2), 129.1 (2CH), 129.2 (2CH), 134.7 (CH), 135.6 (C), 189.3 (C).

1-(3-Ethoxy-4-methoxyphenyl)-2-(methylsulfonyl)ethan-1-one (3h). 50% yield. Purified by column chromatography (eluent gradient 2:1 to 1:1 Hex:EtOAc). R_f (Hex:EtOAc, 2:1): 0.18. Orange solid. Mp: 138-140 ºC. IR (neat): ν 2933, 1663 (C=O), 1514, 1241 (SO_2), 1145, 1116 (SO_2), 806 cm^{-1}. ^{1}H-NMR ($CDCl_3$, 300.13 MHz): δ 1.50 (t, J = 6.9 Hz, 3H), 3.15 (s, 3H), 3.97 (s, 3H), 4.17 (q, J = 7.0 Hz, 2H), 4.57 (s, 2H), 6.95 (d, J = 8.5 Hz, 1H), 7.47-7.69 (m, 2H). ^{13}C-NMR ($CDCl_3$, 75.5 MHz): δ 14.6 (CH_3), 41.7 (CH_3), 56.3 (CH_3), 61.1 (CH_2), 64.5 (CH_2), 110.4 (CH), 111.7 (CH), 124.9 (CH), 128.8 (C), 148.7 (C), 155.1 (C), 187.4 (C).

II.3. General procedure for the reduction of β-keto sulfones 3a-h to produce racemic β-hydroxy sulfones 4a-h

β-Keto sulfones **3a-h** (0.1 mmol) were dissolved in MeOH (1 mL) and cooled to 0 ºC, then $NaBH_4$ (15.1 mg, 0.4 mmol) was added in portions, and the mixture stirred at room temperature. The reaction was monitored by TLC (Hex:EtOAc, 2:1) until complete disappearance of the starting material after 1 h, then the reaction was quenched with H_2O (5.0 mL), and the mixture extracted with EtOAc (3 x 10 mL). The organic layers were combined, dried over Na_2SO_4, filtered and evaporated under reduced pressure, obtaining the corresponding racemic β-hydroxy sulfones **4a-h** (95-97% yield).

(*R*)-1-Phenyl-2-(phenylsulfonyl)ethan-1-ol (4a). 97% yield. R_f (Hex:EtOAc, 2:1): 0.25. Orange oil. IR (neat): ν 3494 (OH), 3061, 1447, 1305 (SO_2), 1275, 1136 (SO_2), 732 cm^{-1}. ^{1}H-NMR ($CDCl_3$, 300.13 MHz): δ 3.37 (dd, *J* = 14.4, 1.9 Hz, 1H), 3.53 (dd, *J* = 14.4, 10.0 Hz, 1H), 5.30 (dd, *J* = 10.0, 1.9 Hz, 1H), 7.26-7.33 (m, 5H), 7.55-7.66 (m, 2H), 7.70 (d, *J* = 7.5 Hz, 1H), 7.92-8.00 (m, 2H). ^{13}C-NMR ($CDCl_3$, 75.5 MHz): δ 64.0 (CH_2), 68.5 (CH), 125.7 (2CH), 128.0 (2CH), 128.4 (CH), 128.8 (2CH), 129.5 (2CH), 134.2 (CH), 139.2 (C), 140.6 (C).

(*S*)-1-(4-Methylphenyl)-2-(phenylsulfonyl)ethan-1-ol (4b). 96% yield. R_f (Hex:EtOAc, 2:1): 0.38. Orange oil. IR (neat): ν 3479 (OH), 3006, 1446, 1267 (SO_2), 1261, 1137 (SO_2), 767 cm^{-1}. ^{1}H-NMR ($CDCl_3$, 300.13 MHz): δ 2.34 (s, 3H), 3.35 (dd, *J* = 14.4, 1.9 Hz, 1H), 3.53 (dd, *J* = 14.3, 10.0 Hz, 1H), 3.61 (br s, 1H), 5.26 (dd, *J* = 10.0, 1.9 Hz, 1H), 7.19 (dd, *J* = 9.6, 7.4 Hz, 4H), 7.59-7.64 (m, 2H), 7.69-7.71 (m, 1H), 7.97-8.00 (d, *J* = 9.0 Hz, 2H). ^{13}C-NMR ($CDCl_3$, 75.5 MHz): δ 21.1 (CH_3), 64.0 (CH_2), 68.3 (CH), 125.6 (2CH), 128.0 (2CH), 129.4 (2CH), 129.5 (2CH), 134.1 (CH), 137.7 (C), 138.2 (C), 139.3 (C).

(*S*)-1-(4-Methoxyphenyl)-2-(phenylsulfonyl)ethan-1-ol (4c). 95% yield. R_f (Hex:EtOAc, 2:1): 0.25. Yellow oil. IR (neat): ν 3480 (OH), 3005, 1512, 1276 (SO_2), 1260, 1135 (SO_2), 749 cm^{-1}. ^{1}H-NMR ($CDCl_3$, 300.13 MHz): δ 3.34 (dd, *J* = 14.3, 2.0 Hz, 1H), 3.53 (dd, *J* = 14.3, 10.0 Hz, 1H), 3.63 (br s, 1H), 3.79 (s, 3H), 5.24 (dd, *J* = 12.0, 3.0 Hz, 1H), 6.86 (d, *J* = 8.7 Hz, 2H), 7.23 (dd, *J* = 6.0, 3.0 Hz, 2H), 7.58-7.64 (m, 2H), 7.68-7.71 (m, 1H), 7.97 (dd, *J* = 9.0, 3.0 Hz, 2H). ^{13}C-NMR ($CDCl_3$, 75.5 MHz): δ 55.3 (CH_3), 63.9 (CH_2), 68.1 (CH), 114.1 (2CH), 127.0 (2CH), 128.0 (2CH), 129.4 (2CH), 132.8 (C), 134.1 (CH), 139.2 (C), 159.6 (C).

(*S*)-1-(4-Bromophenyl)-2-(phenylsulfonyl)ethan-1-ol (4d). 95% yield. R_f (Hex:EtOAc, 2:1): 0.37; Orange oil. IR (neat): ν 3467 (OH), 3005, 1447, 1276 (SO_2), 1261, 1136 (SO_2), 764 cm^{-1}. ^{1}H-NMR ($CDCl_3$, 300.13 MHz): δ 3.32 (dd, *J* = 14.3, 2.0 Hz, 1H), 3.48 (dd, *J* = 14.3, 9.9 Hz,

1H), 3.80 (br s, 1H), 5.28 (dd, J = 9.9, 2.0 Hz, 1H), 7.19 (dd, J = 6.0, 3.0 Hz, 2H), 7.46 (dd, J = 9.0, 3.0 Hz, 2H), 7.60-7.65 (m, 2H), 7.70-7.75 (m, 1H), 7.97 (dd, J = 6.0, 3.0 Hz, 2H). ^{13}C-NMR ($CDCl_3$, 75.5 MHz): δ 63.7 (CH_2), 67.9 (CH), 122.2 (C), 127.4 (2CH), 128.0 (2CH), 129.6 (2CH), 131.9 (2CH), 134.3 (CH), 139.0 (C), 139.7 (C).

(*S*)-1-[(1,1'-Biphenyl)-4-yl]-2-(phenylsulfonyl)ethan-1-ol (4e). 95% yield. Orange oil. R_f (Hex:EtOAc, 2:1): 0.33. IR (neat): ν 3479 (OH), 3028, 1446, 1276 (SO_2), 1261, 1135 (SO_2), 750 cm^{-1}. ^{1}H-NMR ($CDCl_3$, 300.13 MHz): δ 3.42 (dd, 1H, J = 12.0, 3.0 Hz), 3.58 (dd, 1H, J = 15.0, 9.0 Hz), 3.76 (br s, 1H), 5.37 (d, 1H, J = 9.0 Hz), 7.38-7.45 (m, 5H), 7.55-7.64 (m, 6H), 7.65-7.72 (m, 1H), 7.98-8.01 (m, 2H). ^{13}C-NMR ($CDCl_3$, 75.5 MHz): δ 63.9 (CH_2), 68.3 (CH), 126.2 (2CH), 127.1 (2CH), 127.5 (2CH), 128.0 (2CH), 128.8 (2CH), 129.2 (CH), 129.5 (2CH), 134.1 (CH), 139.2 (C), 139.6 (C), 140.5 (C), 141.3 (C).

(*R*)-1-Phenyl-2-tosylethan-1-ol (4f). 97% yield. Orange oil. R_f (Hex:EtOAc, 2:1): 0.35. IR (neat): ν 3483 (OH), 1452, 1285 (SO_2), 1133 (SO_2), 698 cm^{-1}. ^{1}H-NMR ($CDCl_3$, 300.13 MHz): δ 2.49 (s, 3H), 3.34 (dd, J = 14.3, 1.9 Hz, 1H), 3.50 (dd, J = 14.3, 10.0 Hz, 1H), 3.78 (br s, 1H), 5.27 (d, J = 10.0 Hz, 1H), 7.27-7.42 (m, 8H), 7.86 (d, J = 8.0 Hz, 2H). ^{13}C-NMR ($CDCl_3$, 75.5 MHz): δ 21.7 (CH_3), 64.0 (CH_2), 68.5 (CH), 125.7 (2CH), 128.0 (2CH), 128.3 (CH), 128.8 (2CH), 130.1 (2CH), 136.1 (C), 140.7 (C), 145.3 (C).

(*R*)-2-(Methylsulfonyl)-1-phenylethan-1-ol (4g). 96% yield. Yellow solid. R_f (Hex:EtOAc, 2:1): 0.21. Mp= 102-104 ºC. IR (neat): ν 3430 (OH), 1494, 1273 (SO_2), 1233, 1121 (SO_2), 754 cm^{-1}. ^{1}H-NMR ($CDCl_3$, 300.13 MHz): δ 3.04 (s, 3H), 3.14 (d, J = 14.7 Hz, 1H), 3.27 (br s, 1H), 3.45 (dd, J = 14.8, 10.3 Hz, 1H), 5.32 (dd, J = 10.1, 1.9 Hz, 1H), 7.33-7.41 (m, 5H). ^{13}C-NMR ($CDCl_3$, 75.5 MHz): δ 42.9 (CH_3), 62.4 (CH_2), 69.3 (CH), 125.7 (2CH), 128.6 (CH), 129.0 (2 CH), 141.1 (C).

(*S*)-1-(3-Ethoxy-4-methoxyphenyl)-2-(methylsulfonyl)ethan-1-ol (4h). 97% yield. Orange solid. R_f (Hex:EtOAc, 2:1): 0.12. Mp: 119-121 ºC. IR (neat): ν 3458 (OH), 1516, 1256 (SO_2), 1117 (SO_2), 805, 610 cm^{-1}. ^{1}H-NMR ($CDCl_3$, 300.13 MHz): δ 1.49 (t, J = 7.0 Hz, 3H), 3.06 (s, 3H), 3.16 (d, J = 15.0 Hz, 1H), 3.47 (dd, J = 14.7, 10.2 Hz, 1H), 3.88 (s, 3H), 4.11 (q, J = 7.0 Hz, 2H), 5.28 (dd, J = 10.2, 2.2 Hz, 1H), 6.85-6.93 (m, 3H). ^{13}C-NMR ($CDCl_3$, 75.5 MHz): δ 14.8 (CH_3), 42.8 (CH_3), 56.0 (CH_3), 62.6 (CH_2), 64.4 (CH_2), 69.2 (CH), 110.0 (CH), 111.6 (CH), 117.9 (CH), 133.6 (C), 148.7 (C), 149.5 (C).

III. General procedure for enzymatic reduction screenings

III.1. Bioreduction of 3a-h with KREDs from Codexis Inc

The corresponding KRED (1 mg), β-keto sulfone **3a-h**, (7 mM, 555 μL total volume), DMSO (10 µL, 1.8% v/v) and 2-PrOH (95 µL, 17.1% v/v) were added to a 1.5 mL Eppendorf tube containing the mix-P (450 μL of a stock solution of 300 mg mix-P/10 mL of water). The reaction was shaken at 250 rpm and 30 ºC for 24 h. After this time, the mixture was extracted with EtOAc (2 x 500 μL), the organic layers separated by centrifugation (90 s, 13000 rpm), combined and finally dried over Na_2SO_4. Conversions were determined by reverse phase HPLC and enantiomeric excess of the alcohols were measured by HPLC using different chiral columns (see Tables S1-S9). In the case of KRED-101, KRED-119, KRED-130, KRED-NADH-101 and KRED-NADH-110, mix-N (450 μL of a stock solution of 90 mg mix-P/3 mL of water) was used.

III.2. Bioreduction of 3a-h with *Ras*ADH

Lyophilized cells of *Ras*ADH heterologously expressed in *E. coli* (15 mg), β-keto sulfone **3a-h** (25 mM, 600 µL total volume), DMSO (15 µL, 2.5% v/v), an aqueous solution of D-glucose (50 mM, 60 µL, 10% v/v) and GDH-105 (10 U, 70 µL, 11.7% v/v) were added to a 1.5 mL Eppendorf tube with a Tris·HCl buffer pH 7.5 (50 mM, 395 µL) and NADPH (1 mM, 60 µL of a 10 mM NADPH water solution). The reaction was shaken at 250 rpm and 30 ºC for 24 h. After this time, the mixture was extracted with EtOAc (2 x 500 μL), the organic layers separated by centrifugation (90 s, 13000 rpm), combined and finally dried over Na_2SO_4. Conversions were determined by reverse phase HPLC and enantiomeric excess of the alcohols were measured by HPLC using different chiral columns (see Tables S1-S8).

III.3. Bioreduction of 3a-h with *Sy*ADH, ADH-T, *TeS*ADH or ADH-A

Lyophilized cells of the corresponding ADH heterologously expressed in *E. coli* (15 mg), β-keto sulfone **3a-h** (25 mM, 600 µL total volume), DMSO (15 µL, 2.5% v/v) and 2-PrOH (30 μL, 5% v/v) were added to a 1.5 mL Eppendorf tube with a Tris·HCl buffer pH 7.5 (50 mM, 495 µL) containing NADPH (1 mM, 60 µL of a 10 mM NADPH water solution) except for ADH-A that NADH was used. The reaction was shaken at 250 rpm and 30 ºC for 24 h. After this time, the mixture was extracted with EtOAc (2 x 500 μL), the organic layers separated by centrifugation (90 s, 13000 rpm), combined and finally dried over Na_2SO_4. Conversions were determined by

reverse phase HPLC and enantiomeric excess of the alcohols were measured by HPLC using different chiral columns (see Tables S1-S8).

III.4. Bioreduction of 3a-h with*Lb*ADH

Lyophilized cells of *Lb*ADH heterologously expressed in *E. coli* (15 mg), β-keto sulfone **3a-h** (25 mM, 630 µL total volume), DMSO (16 µL, 2.5% v/v) and 2-PrOH (32 µL, 5% v/v) were added to a 1.5 mL Eppendorf tube with a Tris·HCl buffer pH 7.5 (50 mM, 464 µL), $MgCl_2$ (1 mM, 60 µL of a 10 mM $MgCl_2$ water solution) and NADPH (1 mM, 60 µL of a 10 mM NADPH water solution). The reaction was shaken at 250 rpm and 30 ºC for 24 h. After this time, the mixture was extracted with EtOAc (2 x 500 µL), the organic layers separated by centrifugation (90 s, 13000 rpm), combined and finally dried over Na_2SO_4. Conversions were determined by reverse phase HPLC and enantiomeric excess of the alcohols were measured by HPLC using different chiral columns (see Tables S1-S8).

III.5. Bioreduction of 3a-h with *Lk*ADH

Cell-free extract of *Lk*ADH heterologously expressed in *E. coli* (10 µL, 1.5% v/v), β-keto sulfone **3a-h** (7 mM, 627 µL total volume), DMSO (10 µL, 1.5% v/v) and 2-PrOH (67 µL, 11% v/v) were added to a 1.5 mL Eppendorf tube with a KH_2PO_4 buffer pH 7.0 (50 mM, 540 µL, 86% v/v) containing $MgCl_2$ (1 mM, 60 µL of a 10 mM $MgCl_2$ water solution) and NADPH (1 mM, 60 µL of a 10 mM NADPH water solution). The reaction was shaken at 250 rpm and 30 ºC for 24 h. After this time, the mixture was extracted with EtOAc (2 x 500 µL), the organic layers separated by centrifugation (90 s, 13000 rpm), combined and finally dried over Na_2SO_4. Conversions were determined by reverse phase HPLC and enantiomeric excess of the alcohols were measured by HPLC using different chiral columns (see Tables S1-S8).

III.6. Bioreduction of 3a-h with commercial evo-1.1.200 ADH

Commercial evo-1.1.200 ADH (10 mg), β-keto sulfone **3a-h** (25 mM, 450 µL total volume), DMSO (12 µL, 2.5% v/v) and 2-PrOH (25 µL, 5.5% v/v) were added to a 1.5 mL Eppendorf tube with a Tris·HCl buffer pH 7.5 (50 mM, 314 µL), $MgCl_2$ (1 mM, 50 µL of a 10 mM $MgCl_2$ water solution) and NADPH (1 mM, 50 µL of a 10 mM NADPH water solution). The reaction was shaken at 250 rpm and 30 ºC for 24 h. After this time, the mixture was extracted with EtOAc (2 x 500 µL), the organic layers separated by centrifugation (90 s, 13000 rpm), combined and finally dried over Na_2SO_4. Conversions were determined by reverse phase HPLC

and enantiomeric excess of the alcohols were measured by HPLC using different chiral columns (see Tables S1-S8).

III.7. Results of the bioreduction screenings

After following the previously described general procedures, the results from the bioreduction screenings over β-keto sulfone **3a-h** are depicted in Tables S1 to S9.

Table S1. Bioreduction of 1-phenyl-2-(phenylsulfonyl)ethan-1-one (**3a**).[a]

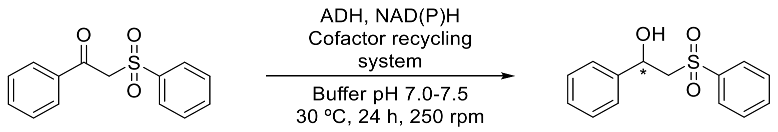

Entry	Enzyme	*c* (%)[b]	*ee* (%)[c]	Entry	Enzyme	*c* (%)[b]	*ee* (%)[c]
1	KRED-101	35	n.d.	15	KRED-P1-A04	<1	---
2	KRED-119	>99	98 (*S*)	16	KRED-P2-H07	<1	---
3	KRED-130	>99	86 (*R*)	17	KRED-P2-D03	>99	26 (*R*)
4	KRED-NADH-101	<1	---	18	KRED-P2-D11	52	86 (*R*)
5	KRED-NADH-110	>99	92 (*S*)	19	KRED-P2-D12	>99	16 (*R*)
6	KRED-P1-B12	>99	>99 (*R*)	20	*Ras*ADH	>99	>99 (*R*)
7	KRED-P1-B02	>99	>99 (*R*)	21	*Sy*ADH	59	18 (*R*)
8	KRED-P1-B05	>99	20 (*R*)	22	ADH-T	<1	---
9	KRED-P1-B10	>99	>99 (*R*)	23	*TeS*ADH	<1	---
10	KRED-P2-C02	>99	90 (*R*)	24	ADH-A	<1	---
11	KRED-P2-C11	20	n.d.	25	*Lb*ADH	<1	---
12	KRED-P1-H08	>99	71 (*R*)	26	*Lk*ADH	<1	---
13	KRED-P3-G09	<1	---	27	evo.1.1.200	31	>99 (*R*)
14	KRED-P2-B02	>99	80 (*R*)				

[a] Best results, already highlighted in the main manuscript, appear in red colour.
[b] Conversion values were measured by HPLC.
[c] Enantiomeric excess values of the β-hydroxy sulfone **4a** were measured by HPLC using a Chiralpak AD-H column. The major enantiomer appears in parentheses. n.d.: Not determined.

Table S2. Bioreduction of 1-(4-methylphenyl)-2-(phenylsulfonyl)ethan-1-one (**3b**).[a]

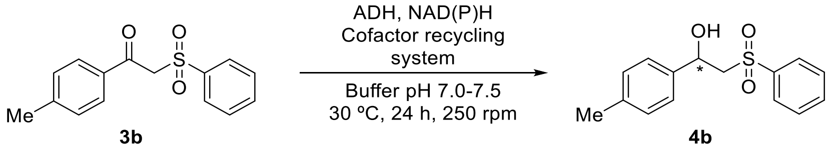

Entry	Enzyme	*c* (%)[b]	*ee* (%)[c]	Entry	Enzyme	*c* (%)[b]	*ee* (%)[c]
1	KRED-101	17	n.d.	15	KRED-P1-A04	<1	---
2	KRED-119	>99	>99 (*S*)	16	KRED-P2-H07	<1	---
3	KRED-130	>99	>99 (*R*)	17	KRED-P2-D03	>99	10 (*S*)
4	KRED-NADH-101	<1	---	18	KRED-P2-D11	15	n.d.
5	KRED-NADH-110	28	n.d.	19	KRED-P2-D12	>99	64 (*S*)
6	KRED-P1-B12	35	n.d.	20	*Ras*ADH	86	>99 (*R*)
7	KRED-P1-B02	>99	>99 (*R*)	21	*Sy*ADH	55	80 (*S*)
8	KRED-P1-B05	>99	91 (*S*)	22	ADH-T	<1	---
9	KRED-P1-B10	19	n.d.	23	*Te*SADH	<1	---
10	KRED-P2-C02	>99	20 (*R*)	24	ADH-A	<1	---
11	KRED-P2-C11	5	n.d.	25	*Lb*ADH	<1	---
12	KRED-P1-H08	28	n.d.	26	*Lk*ADH	<1	---
13	KRED-P3-G09	<1	---	27	evo.1.1.200	20	n.d.
14	KRED-P2-B02	>99	20 (*R*)				

[a] Best results, already highlighted in the main manuscript, appear in red colour.

[b] Conversion values were measured by HPLC.

[c] Enantiomeric excess values of the β-hydroxy sulfone **4b** were measured by HPLC using a Chiralpak AD-H column. The major enantiomer appears in parentheses. n.d.: Not determined.

For the determination of optical rotation values (see Section VIII), the bioreduction of **3b** was carried out with 10 mg of substrate, using KRED-119 and leading to enantiopure (*S*)-**4b** in 92% yield.

Table S3. Bioreduction of 1-(4-methoxyphenyl)-2-(phenylsulfonyl)ethan-1-one (**3c**).[a]

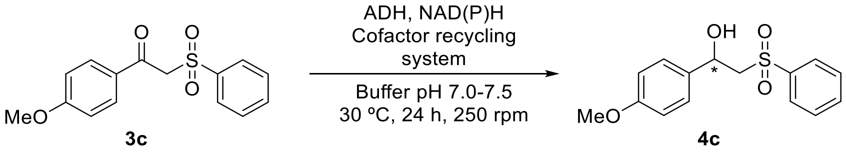

Entry	Enzyme	*c* (%)[b]	*ee* (%)[c]	Entry	Enzyme	*c* (%)[b]	*ee* (%)[c]
1	KRED-101	<1	---	15	KRED-P1-A04	<1	---
2	KRED-119	>99	92 (*S*)	16	KRED-P2-H07	<1	---
3	KRED-130	40	n.d.	17	KRED-P2-D03	41	34 (*R*)
4	KRED-NADH-101	<1	---	18	KRED-P2-D11	<1	---
5	KRED-NADH-110	18	n.d.	19	KRED-P2-D12	10	n.d.
6	KRED-P1-B12	<1	---	20	*Ras*ADH	95	>99 (*R*)
7	KRED-P1-B02	<1	---	21	*Sy*ADH	6	n.d.
8	KRED-P1-B05	94	>99 (*S*)	22	ADH-T	<1	---
9	KRED-P1-B10	<1	---	23	*TeS*ADH	<1	---
10	KRED-P2-C02	10	n.d.	24	ADH-A	<1	---
11	KRED-P2-C11	<1	---	25	*Lb*ADH	<1	---
12	KRED-P1-H08	<1	---	26	*Lk*ADH	<1	---
13	KRED-P3-G09	<1	---	27	evo.1.1.200	<1	---
14	KRED-P2-B02	57	16 (*R*)				

[a] Best results, already highlighted in the main manuscript, appear in red colour.
[b] Conversion values were measured by HPLC.
[c] Enantiomeric excess values of the β-hydroxy sulfone **4c** were measured by HPLC using a Chiralcel OJ-H column. The major enantiomer appears in parentheses. n.d.: Not determined.

For the determination of optical rotation values (see Section VIII), the bioreduction of **3c** was carried out with 10 mg of substrate, using KRED-P1-B05 and leading to enantiopure (*S*)-**4c** in 94% yield.

Table S4. Bioreduction of 1-(4-bromophenyl)-2-(phenylsulfonyl)ethan-1-one (**3d**).[a]

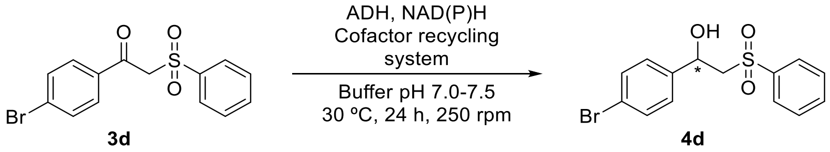

Entry	Enzyme	*c* (%)[b]	*ee* (%)[c]	Entry	Enzyme	*c* (%)[b]	*ee* (%)[c]
1	KRED-101	7	n.d.	15	KRED-P1-A04	<1	---
2	KRED-119	>99	>99 (*S*)	16	KRED-P2-H07	<1	---
3	KRED-130	97	>99 (*R*)	17	KRED-P2-D03	94	30 (*R*)
4	KRED-NADH-101	8	n.d.	18	KRED-P2-D11	9	n.d.
5	KRED-NADH-110	64	86 (*S*)	19	KRED-P2-D12	91	52 (*S*)
6	KRED-P1-B12	31	n.d.	20	*Ras*ADH	91	>99 (*R*)
7	KRED-P1-B02	94	>99 (*R*)	21	*Sy*ADH	>99	96 (*S*)
8	KRED-P1-B05	>99	>99 (*S*)	22	ADH-T	<1	---
9	KRED-P1-B10	31	n.d.	23	*TeS*ADH	<1	---
10	KRED-P2-C02	97	68 (*R*)	24	ADH-A	<1	---
11	KRED-P2-C11	10	n.d.	25	*Lb*ADH	<1	---
12	KRED-P1-H08	21	n.d.	26	*Lk*ADH	<1	---
13	KRED-P3-G09	<1	---	27	evo.1.1.200	24	n.d.
14	KRED-P2-B02	90	6 (*R*)				

[a] Best results, already highlighted in the main manuscript, appear in red colour.
[b] Conversion values were measured by HPLC.
[c] Enantiomeric excess values of the β-hydroxy sulfone **4d** were measured by HPLC using a Chiralcel OJ-H column. The major enantiomer appears in parentheses. n.d.: Not determined.

For the determination of optical rotation values (see Section VIII), the bioreduction of **3d** was carried out with 10 mg of substrate, using KRED-P1-B05 and leading to enantiopure (*S*)-**4d** in 95% yield.

Table S5. Bioreduction of 1-[(1,1'-biphenyl)-4-yl]-2-(phenylsulfonyl)ethan-1-one (**3e**).[a]

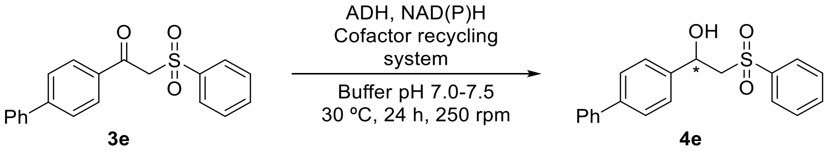

Entry	Enzyme	*c* (%)[b]	*ee* (%)[c]	Entry	Enzyme	*c* (%)[b]	*ee* (%)[c]
1	KRED-101	10	n.d.	15	KRED-P1-A04	<1	---
2	KRED-119	92	>99 (*S*)	16	KRED-P2-H07	<1	---
3	KRED-130	55	>99 (*R*)	17	KRED-P2-D03	30	n.d.
4	KRED-NADH-101	<1	---	18	KRED-P2-D11	5	n.d.
5	KRED-NADH-110	3	n.d.	19	KRED-P2-D12	8	n.d.
6	KRED-P1-B12	<1	---	20	*Ras*ADH	60	>99 (*R*)
7	KRED-P1-B02	7	n.d.	21	*Sy*ADH	22	n.d.
8	KRED-P1-B05	89	>99 (*S*)	22	ADH-T	<1	---
9	KRED-P1-B10	<1	---	23	*TeS*ADH	<1	---
10	KRED-P2-C02	41	>99 (*R*)	24	ADH-A	<1	---
11	KRED-P2-C11	<1	---	25	*Lb*ADH	<1	---
12	KRED-P1-H08	15	n.d.	26	*Lk*ADH	<1	---
13	KRED-P3-G09	<1	---	27	evo.1.1.200	5	n.d.
14	KRED-P2-B02	28	n.d.				

[a] Best results, already highlighted in the main manuscript, appear in red colour.
[b] Conversion values were measured by HPLC.
[c] Enantiomeric excess values of the β-hydroxy sulfone **4e** were measured by HPLC using a Chiralpak AD-H column. The major enantiomer appears in parentheses. n.d.: Not determined.

For the determination of optical rotation values (see Section VIII), the bioreduction of **3e** was carried out with 10 mg of substrate, using KRED-119 and leading to enantiopure (*S*)-**4e** in 90% yield.

Table S6. Bioreduction of 1-phenyl-2-tosylethan-1-one (**3f**).[a]

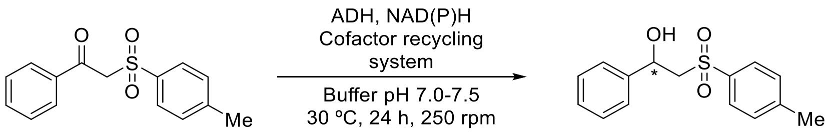

Entry	Enzyme	*c* (%)[b]	*ee* (%)[c]	Entry	Enzyme	*c* (%)[b]	*ee* (%)[c]
1	KRED-101	31	n.d.	15	KRED-P1-A04	<1	---
2	KRED-119	>99	93 (*S*)	16	KRED-P2-H07	45	n.d.
3	KRED-130	82	92 (*R*)	17	KRED-P2-D03	>99	31 (*S*)
4	KRED-NADH-101	7	n.d.	18	KRED-P2-D11	10	n.d.
5	KRED-NADH-110	93	96 (*S*)	19	KRED-P2-D12	93	22 (*R*)
6	KRED-P1-B12	94	>99 (*R*)	20	*Ras*ADH	97	95 (*R*)
7	KRED-P1-B02	>99	>99 (*R*)	21	*Sy*ADH	60	14 (*S*)
8	KRED-P1-B05	>99	60 (*R*)	22	ADH-T	<1	---
9	KRED-P1-B10	>99	>99 (*R*)	23	*Te*SADH	<1	---
10	KRED-P2-C02	>99	70 (*R*)	24	ADH-A	<1	---
11	KRED-P2-C11	5	n.d.	25	*Lb*ADH	<1	---
12	KRED-P1-H08	35	n.d.	26	*Lk*ADH	<1	---
13	KRED-P3-G09	16	n.d.	27	evo.1.1.200	13	n.d.
14	KRED-P2-B02	94	56 (*R*)				

[a] Best results, already highlighted in the main manuscript, appear in red colour.
[b] Conversion values were measured by HPLC.
[c] Enantiomeric excess values of the β-hydroxy sulfone **4f** were measured by HPLC using a Chiralpak AD-H column. The major enantiomer appears in parentheses. n.d.: Not determined.

For the determination of optical rotation values (see Section VIII), the bioreduction of **3f** was carried out with 10 mg of substrate, using KRED-P1-B10 and leading to enantiopure (*R*)-**4f** in 94% yield.

Table S7. Bioreduction of 2-(methylsulfonyl)-1-phenylethan-1-one (**3g**).[a]

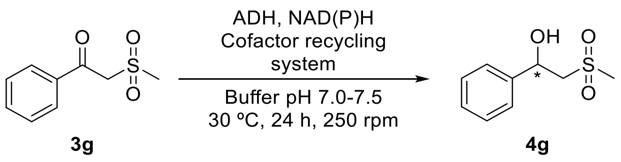

Entry	Enzyme	*c* (%)[b]	*ee* (%)[c]	Entry	Enzyme	*c* (%)[b]	*ee* (%)[c]
1	KRED-101	7	n.d.	15	KRED-P1-A04	<1	---
2	KRED-119	>99	>99 (*S*)	16	KRED-P2-H07	<1	---
3	KRED-130	30	n.d.	17	KRED-P2-D03	70	4 (*S*)
4	KRED-NADH-101	<1	---	18	KRED-P2-D11	98	86 (*R*)
5	KRED-NADH-110	29	n.d.	19	KRED-P2-D12	>99	4 (*S*)
6	KRED-P1-B12	>99	86 (*R*)	20	*Ras*ADH	>99	98 (*R*)
7	KRED-P1-B02	>99	>99 (*R*)	21	*Sy*ADH	>99	98 (*R*)
8	KRED-P1-B05	>99	>99 (*S*)	22	ADH-T	<1	---
9	KRED-P1-B10	>99	>99 (*R*)	23	*Te*SADH	<1	---
10	KRED-P2-C02	>99	2 (*R*)	24	ADH-A	<1	---
11	KRED-P2-C11	24	n.d.	25	*Lb*ADH	<1	---
12	KRED-P1-H08	88	38 (*R*)	26	*Lk*ADH	<1	---
13	KRED-P3-G09	5	n.d.	27	evo.1.1.200	15	n.d.
14	KRED-P2-B02	>99	70 (*S*)				

[a] Best results, already highlighted in the main manuscript, appear in red colour.

[b] Conversion values were measured by HPLC.

[c] Enantiomeric excess values of the β-hydroxy sulfone **4g** were measured by HPLC using a Chiralpak AD-H column. The major enantiomer appears in parentheses. n.d.: Not determined.

For the determination of optical rotation values (see Section VIII), the bioreduction of **3g** was carried out with 10 mg of substrate, using KRED-P1-B10 and leading to enantiopure (*R*)-**4g** in 90% yield.

Table S8. Bioreduction of 1-(3-ethoxy-4-methoxyphenyl)-2-(methylsulfonyl)ethan-1-one (**3h**).[a]

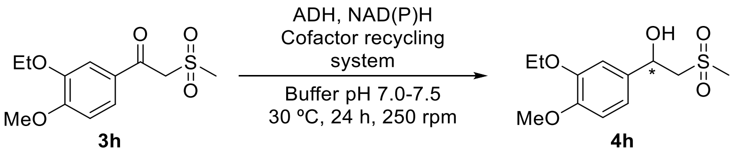

Entry	Enzyme	*c* (%)[b]	*ee* (%)[c]	Entry	Enzyme	*c* (%)[b]	*ee* (%)[c]
1	KRED-101	<1	---	15	KRED-P1-A04	<1	---
2	KRED-119	95	8 (*S*)	16	KRED-P2-H07	<1	---
3	KRED-130	<1	---	17	KRED-P2-D03	34	98 (*S*)
4	KRED-NADH-101	<1	---	18	KRED-P2-D11	97	50 (*R*)
5	KRED-NADH-110	<1	---	19	KRED-P2-D12	42	95 (*S*)
6	KRED-P1-B12	<1	---	20	*Ras*ADH	93	80 (*R*)
7	KRED-P1-B02	20	n.d.	21	*Sy*ADH	<1	---
8	KRED-P1-B05	<1	---	22	ADH-T	<1	---
9	KRED-P1-B10	<1	---	23	*TeS*ADH	<1	---
10	KRED-P2-C02	19	n.d.	24	ADH-A	<1	---
11	KRED-P2-C11	5	n.d.	25	*Lb*ADH	<1	---
12	KRED-P1-H08	<1	---	26	*Lk*ADH	<1	---
13	KRED-P3-G09	<1	---	27	evo.1.1.200	<1	---
14	KRED-P2-B02	90	44 (*R*)				

[a] Best results, already highlighted in the main manuscript, appear in red colour.

[b] Conversion values were measured by HPLC.

[c] Enantiomeric excess values of the β-hydroxy sulfone **4h** were measured by HPLC using a Chiralpak AD-H column. The major enantiomer appears in parentheses. n.d.: Not determined.

Additional optimization was performed with the best enzymes (KRED-P2-D03 and KRED-P2-D12) in order to obtain higher conversion values (Table S9).

Table S9. Bioreduction of β-keto sulfone **3h** varying different reaction parameters.

Entry	KRED	[3h] (mM)	DMSO (% v/v)	T (ºC)	*c* (%)[a]	*ee* (%)[b]
1	KRED-P2-D03	7	10	30	17	n.d.
2	KRED-P2-D12	7	10	30	49	98 (*S*)
3	KRED-P2-D03	7	10	45	3	--
4	KRED-P2-D12	7	10	45	7	--
5	KRED-P2-D03	7	1.5	45	5	--
6	KRED-P2-D12	7	1.5	45	10	--
7	KRED-P2-D03	5	1.5	30	45	98 (*S*)
8	KRED-P2-D12	5	10	30	41	98 (*S*)
9	KRED-P2-D03	2.5	1.5	30	24	n.d.
10	KRED-P2-D12	2.5	10	30	30	n.d.

[a] Conversion values were measured by HPLC.

[b] Enantiomeric excess values of the β-hydroxy sulfone **4h** were measured by HPLC using a Chiralpak AD-H column. The major enantiomer appears in parentheses. n.d.: Not determined.

IV. Study of the dilution in the sequential process

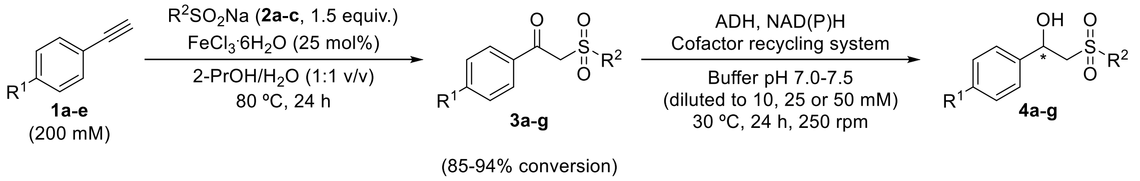

Table S10. Influence of the dilution in the sequential oxosulfonylation-bioreduction approach.

Entry	3a-g	KRED	[3a-g] (mM)	4a-g (%)[a]	*ee* (%)[b]
1	**3a**	KRED-119	10	>99	>99 (*S*)
2		KRED-P1-B02	10	>99	>99 (*R*)
3		KRED-119	50	>99	96 (*S*)
4		KRED-P1-B02	50	>99	>99 (*R*)
5		*Ras*ADH	25	>99	>99 (*R*)
6	**3b**	KRED-119	10	>99	>99 (*S*)
7		KRED-P1-B02	10	>99	>99 (*R*)
8		KRED-119	50	98	>99 (*S*)
9		KRED-P1-B02	50	90	>99 (*R*)
10		*Ras*ADH	25	>99	>99 (*R*)
11	**3c**	KRED-119	10	>99	>99 (*S*)
12		KRED-P1-B05	10	>99	>99 (*R*)
13		KRED-119	50	>99	>99 (*S*)
14		KRED-P1-B05	50	>99	>99 (*R*)
15	**3d**	KRED-119	10	>99	>99 (*S*)
16		KRED-130	10	>99	>99 (*R*)
17		KRED-119	50	>99	>99 (*S*)
18		KRED-130	50	45	>99 (*R*)
19	**3e**	KRED-119	10	92	>99 (*S*)
20		KRED-130	10	>99	>99 (*R*)
21		KRED-P1-B05	10	>99	>99 (*S*)
22		KRED-119	50	77	>99 (*S*)
23		KRED-130	50	>99	>99 (*R*)
24		KRED-P1-B05	50	94	>99 (*S*)

[a] Conversion values were measured by HPLC. [b] Enantiomeric excess values of the β-hydroxy sulfones were measured by chiral HPLC. The major enantiomer appears in parentheses.

Table S10 continuation. Influence of the dilution in the sequential oxosulfonylation-bioreduction approach.

Entry	3a-g	KRED	[3a-g] (mM)	4a-g (%)[a]	*ee* (%)[b]
25	**3f**	KRED-119	10	>99	94 (*S*)
26		KRED-P1-B02	10	>99	>99 (*R*)
27		KRED-119	50	>99	94 (*S*)
28		KRED-P1-B02	50	>99	>99 (*R*)
29	**3g**	KRED-119	10	>99	>99 (*S*)
30		KRED-P1-B02	10	>99	>99 (*R*)
31		KRED-119	50	>99	>99 (*S*)
32		KRED-P1-B05	50	>99	>99 (*S*)

[a] Conversion values were measured by HPLC.

[b] Enantiomeric excess values of the β-hydroxy sulfones were measured by chiral HPLC. The major enantiomer appears in parentheses.

Reactions were scaled-up with:

- substrate **1a** (0.385 mmol) and *Ras*ADH, yielding 53% of (*R*)-**4a** in >99% *ee*
- substrate **1f** (0.385 mmol) and *Ras*ADH, yielding 26% of (*R*)-**4h** in 79% *ee*

These experiments have also allowed to measure the optical rotation values of the so-obtained products.

V. Semi-preparative scale-up for the oxosulfonylation-bioreduction sequence to produce (*R*)-4a

V.1. Sequential transformation using KRED-P1-B02

Phenylacetylene (**1a**, 42 μL, 0.39 mmol) was dissolved in a mixture of 2-PrOH:H_2O (1.93 mL, 1:1 v/v) inside a 25 mL round-bottom flask. Then, sodium benzenesulfinate (**2a**, 94 mg, 0.58 mmol), and $FeCl_3·6H_2O$ (26 mg, 25 mol%) were added, and the mixture stirred at 80 ºC for 24 h under air atmosphere. After this time, the reaction mixture was allowed to cool to room temperature and diluted 4 times (final concentration: 50 mM) with DMSO (308 μL, 4% v/v), 2-PrOH (160 μL, 15% v/v in the final mixture) and mix-P (5.3 mL, 69% v/v). Subsequently, KRED-P1-B02 (100 mg) was added, and the resulting mixture shaken at 250 rpm and 30 ºC for 24 h. After this time, full conversion was observed by HPLC analysis (see section VI.1), so the mixture was extracted with EtOAc (3 x 20 mL). The organic layers were combined, dried over Na_2SO_4.and evaporated under reduced pressure. The reaction crude was finally purified by column chromatography on silica gel (eluent: $CHCl_3$), obtaining enantiopure (*R*)-**4a** (66 mg, 66% yield) as an orange oil.

V.2. Sequential transformation using *Ras*ADH

Phenylacetylene (**1a**, 42 μL, 0.39 mmol) was dissolved in a mixture of 2-PrOH:H_2O (1.93 mL, 1:1 v/v) inside a 25 mL round-bottom flask. Then, sodium benzenesulfinate (**2a**, 94 mg, 0.58 mmol), and $FeCl_3·6H_2O$ (26 mg, 25 mol%) were added, and the mixture was stirred at 80 ºC for 24 h under air atmosphere. After this time, the reaction mixture was cooled to room temperature and diluted 8 times (final concentration: 25 mM) with DMSO (385 μL, 2.5% v/v) and a Tris·HCl buffer pH 7.5 (50 mM, 8.14 mL, 53% v/v) containing NADPH (1 mM, 1.54 mL of a 10 mM NADPH water solution), D-glucose (50 mM, 1.54 mL, 10% v/v), and GDH-105 (10 U, 1.80 mL, 11.7% v/v) was added. Subsequently, *E. coli/Ras*ADH (150 mg) was added, and the resulting mixture shaken at 30 ºC and 250 rpm for 24 h. Finally, the mixture was extracted with EtOAc (3 x 20 mL), and the organic layers separated by centrifugation (3 min, 13000 rpm), to be later combined, dried over Na_2SO_4 and evaporated under reduced pressure. The reaction crude was finally purified by column chromatography on silica gel (eluent: $CHCl_3$), obtaining enantiopure (*R*)-**4a** (53 mg, 53% yield) as an orange oil. $[\alpha]_D^{20}$= –27.0 (c 1.0, $CHCl_3$).

VI. EATOS calculations

Environmental E-factor calculations (Figures S4-7) were performed using the EATOS (v. 1.1) software tool.[9] Due to the fact that some experimental data made by other research groups were missing regarding the exact quantities used of solvents and silica gel for purification of β-hydroxy sulfone **4a**, it was assumed that these quantities needed for isolation and purification would be comparatively the same for all the protocols, and therefore, the results shown in this study are based on the reagents, catalysts and solvents used in the reaction sequences. All transformations were treated as proceeding to the corresponding isolated yields, hence all losses in yield are accounted for as 'unknown by-products'.[10]

**Oxosulfonylation-Bioreduction (this contribution)**						
**Hydroxysulfone 4a**						
isolated yield:	66%					
**Material**	**(Non)volatile [mg]**	**(Non)volatile [E]**	**Solvent [mL]**		**E factor:**	
Starting Materials					starting materials	2,2
substrate **1a**	39,8				solvents	110,9
compound **2a**	95,2	0,462			auxiliaries (reaction)	0,7
O_2	6,4	0,002			by-products	1,1
water	7,2	0,003				
2-propanol	24,0	0,009			**total**	**114,9**
KRED-P1-B02	100,0	1,481				
$FeCl_3$	8,1	0,234				
Solvents						
water		92,785	6,27			
2-propanol		13,096	1,13			
dimethylsulfoxide		5,018	0,31			
Auxiliaries (reaction)						
phosphate salt	43,4	0,643				
NADP cofactor	4,0	0,058				
$MgCl_2$	0,5	0,008				
By-products						
unknown by-products		0,708				
NaOH		0,153				
acetone		0,221				

Figure S4. Calculated E-factor for the oxosulfonylation-bioreduction protocol shown in this contribution to synthesize β-hydroxy sulfone **4a**.

Nucleophilic substitution-Ru-catalyzed reduction (Liu et al., Org. Lett. 2014, 16, 5764-5767)						
**Hydroxysulfone 4a**						
isolated yield:	95%					
**Material**	**(Non)volatile [mg]**	**(Non)volatile [E]**	**Solvent [mL]**		**E factor:**	
Starting Materials					starting materials	0,7
2-bromoacetophenone	19,9				solvents	135,4
compound **2a**	18,1	0,066			auxiliaries (reaction)	0,0
sodium formate	20,4	0,546			by-products	0,8
water	2,0	0,007				
Ru catalyst	3,4	0,126			**total**	**137,0**
Solvents						
water		40,127	1,00			
methanol		95,319	3,00			
By-products						
unknown by-products		0,101				
NaBr		0,392				
NaOH		0,153				
CO_2		0,168				

Figure S5. Calculated E-factor for the nucleophilic substitution-Ru-catalyzed reduction protocol shown by Liu *et al.* to synthesize β-hydroxy sulfone **4a**.[11]

Oxosulfonylation-Ru-catalyzed reduction (Zhou et al., Green Chem. 2019, 21, 634-639)						
**Hydroxysulfone 4a**						
isolated yield:	82%					
**Material**	**(Non)volatile [mg]**	**(Non)volatile [E]**	**Solvent [mL]**		**E factor:**	
Starting Materials					starting materials	3,5
substrate **1a**	20,4				solvents	78,5
compound **2a**	49,4	0,382			auxiliaries (reaction)	0,0
sodium formate	136,0	2,845			by-products	0,8
oxygen	3,4	0,004				
water	7,6	0,008			**total**	**82,7**
$FeCl_3$	6,5	0,151				
Ru catalyst	3,1	0,072				
Solvents						
water		23,245	1,00			
methanol		55,215	3,00			
By-products						
unknown by-products		0,323				
NaOH		0,305				
CO_2		0,168				

Figure S6. Calculated E-factor for the oxosulfonylation-Ru-catalyzed reduction sequence shown by Zhou *et al.* to synthesize β-hydroxy sulfone **4a**.[12]

Immobilized oxosulfonylation-Ru-catalyzed reduction (Liu et al., ChemCatChem 2021, 13, 909-915)

Hydroxysulfone 4a

isolated yield: 91%

Material	(Non)volatile [mg]	(Non)volatile [E]	Solvent [mL]	E factor:	
Starting Materials				starting materials	3,1
substrate **1a**	20,4			solvents	70,3
compound **2a**	49,4	0,344		auxiliaries (reaction)	0,9
sodium formate	136,0	2,564		by-products	0,6
oxygen	3,4	0,003			
water	7,6	0,008		**total**	**74,9**
$FeCl_3$	6,5	0,136			
Ru catalyst	3,1	0,065			
Solvents					
water		20,946	1,00		
ethanol		49,327	3,00		
Auxiliaries (reaction)					
solid carrier	40,8	0,854			
By-products					
unknown by-products		0,146			
NaOH		0,305			
CO_2		0,168			

Figure S7. Calculated E-factor for the oxosulfonylation-Ru-catalyzed reduction sequence in a solid carrier shown by Liu *et al.* to synthesize β-hydroxy sulfone **4a**.[13]

VII. HPLC analyses for the determination of reaction conversion and product enantiomeric excess values

VII.1. HPLC analyses for determination of reaction conversion values

HPLC analyses were carried out in an Agilent chromatograph using a reverse phase column (Zorbax Eclipse XDB-C18, RR, 1.8 μm, 4.6 x 50 mm, Agilent). As eluent, mixtures of acetonitrile (MeCN) and water with 0.1% trifluoroacetic acid (TFA) were used (Tables S11 and S12).

Samples were eluted at flow rate of 2 mL/min with three linear gradients from 10% to 60% v/v MeCN during 5.70 min, followed by another from 60% to 100% v/v MeCN during 0.5 min and a third one from 100% to 10% v/v MeCN during 1.90 min.

Detection and spectral characterization of peaks (UV absorption maxima) were performed with a diode array detector and ChemStation Rev.B.03.01 software (Agilent).

Table S11. Retention times in the HPLC for the metal-catalyzed step.

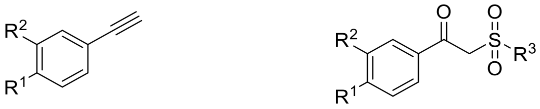

1a-f 3a-h

Compound	Retention time (min)	
	Alkyne	β-keto sulfone
R^1= H, R^2= H, R^3= Ph (**1a** and **3a**)[a]	4.5	4.3
R^1= Me, R^2= H, R^3= Ph (**1b** and **3b**)[a]	5.3	4.9
R^1= OMe, R^2= H, R^3= Ph (**1c** and **3c**)[a]	5.2	4.9
R^1= Br, R^2= H, R^3= Ph (**1d** and **3d**)[a]	6.0	5.4
R^1= Ph, R^2= H, R^3= Ph (**1e** and **3e**)[a]	6.5	6.1
R^1= H, R^2= H, R^3= 4-Me-C_6H_4 (**1a** and **3f**)[a]	4.5	4.6
R^1= H, R^2= H, R^3= Me (**1a** and **3g**)[b]	4.5	2.0
R^1= OMe, R^2= OEt, R^3= Me (**1f** and **3h**)[a]	4.6	2.7

[a] Peak detection was performed at λ = 228 nm.

[b] Peak detection was performed at λ = 218 nm.

Table S12. Retention times in the HPLC for the bioreduction step.

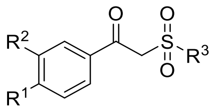

3a-h

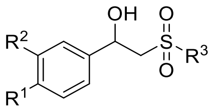

4a-h

**Compound**[a]	**Retention time (min)**	
	β-keto sulfone	β-hydroxy sulfone
R^1= H, R^2= H, R^3= Ph (**a**)	4.3	3.6
R^1= Me, R^2= H, R^3= Ph (**b**)	4.9	4.3
R^1= OMe, R^2= H, R^3= Ph (**c**)	4.9	4.3
R^1= Br, R^2= H, R^3= Ph (**d**)	5.4	4.7
R^1= Ph, R^2= H, R^3= Ph (**e**)	6.1	5.5
R^1= H, R^2= H, R^3= 4-Me-C_6H_4 (**f**)	4.6	4.1
R^1= H, R^2= H, R^3= Me (**g**)	2.0	1.5
R^1= OMe, R^2= OEt, R^3= Me (**h**)	2.7	1.9

[a] Peak detection was performed at λ = 210 nm.

VII.2. HPLC analyses for determination of β-hydroxy sulfone enantiomeric excess values

Table S13. Analytical condition and retention times for the measurement of β-hydroxy sulfone enantiomeric excess values.

**β-Hydroxy sulfone**[a]	**Column**	**Eluent (Hex/2-PrOH)**	**Flow (mL/min)**	**T (°C)**	**Retention time (min)**
**4a**	AD-H	90:10	1.0	25	31.9 (*R*), 36.8 (*S*)
**4b**	AD-H	90:10	1.0	25	36.6 (*R*), 39.7 (*S*)
**4c**	OJ-H	80:20	1.0	25	44.8 (*R*), 61.7 (*S*)
**4d**	OJ-H	80:20	1.0	25	30.9 (*R*), 39.2 (*S*)
**4e**	AD-H	90:10	1.0	25	57.0 (*R*), 62.3 (*S*)
**4f**	AD-H	90:10	1.0	25	29.7 (*R*), 47.0 (*S*)
**4g**	AD-H	90:10	1.0	25	12.8 (*S*), 14.4 (*R*)
**4h**	AD-H	90:10	1.0	25	20.6 (*S*), 22.1 (*R*)

[a] Peak detection was performed at λ = 210 nm.

VII.3. Calibration curves for conversion values determination

VII.3.1. Calibration curve for conversion of phenylacetylene (1a) into 1-phenyl-2-(phenylsulfonyl)ethan-1-one (3a) at 228 nm wavelength

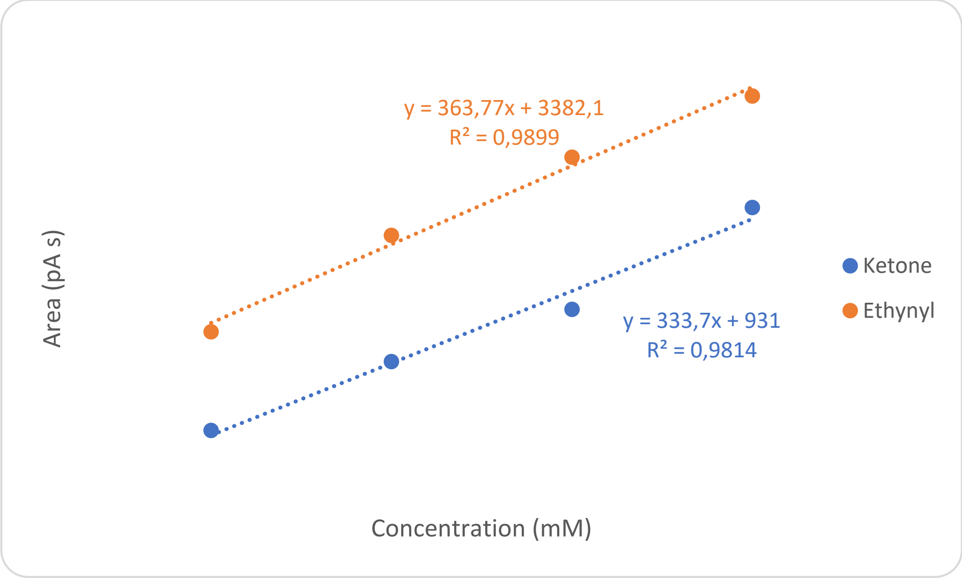

Correction factor = 1.090

VII.3.2. Calibration curve for conversion of phenylacetylene (1a) into 1-phenyl-2-tosylethan-1-one (3f) at 228 nm wavelength

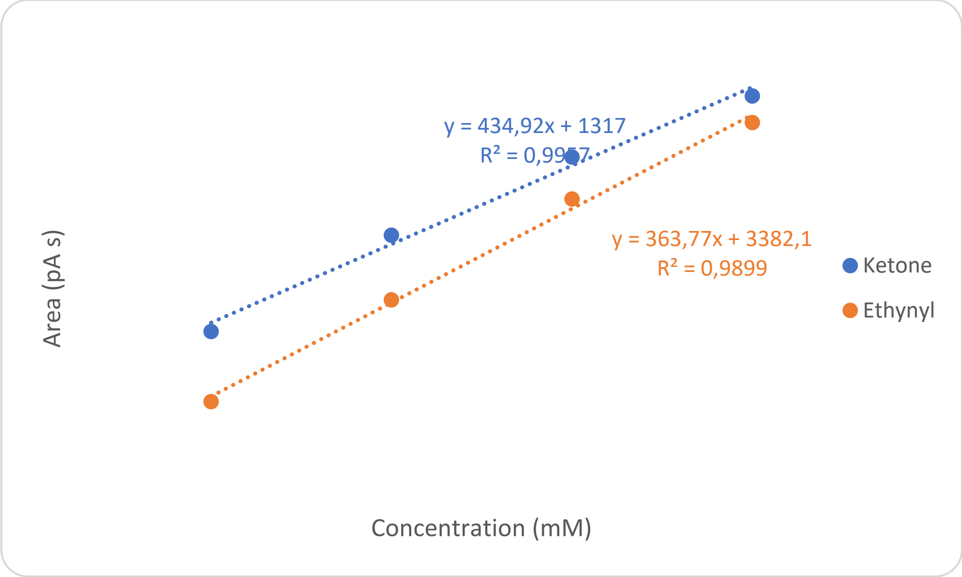

Correction factor = 1.196

VII.3.3. Calibration curve for conversion of phenylacetylene (1a) into 2-(methylsulfonyl)-1-phenylethan-1-one (3g) at 218 nm wavelength

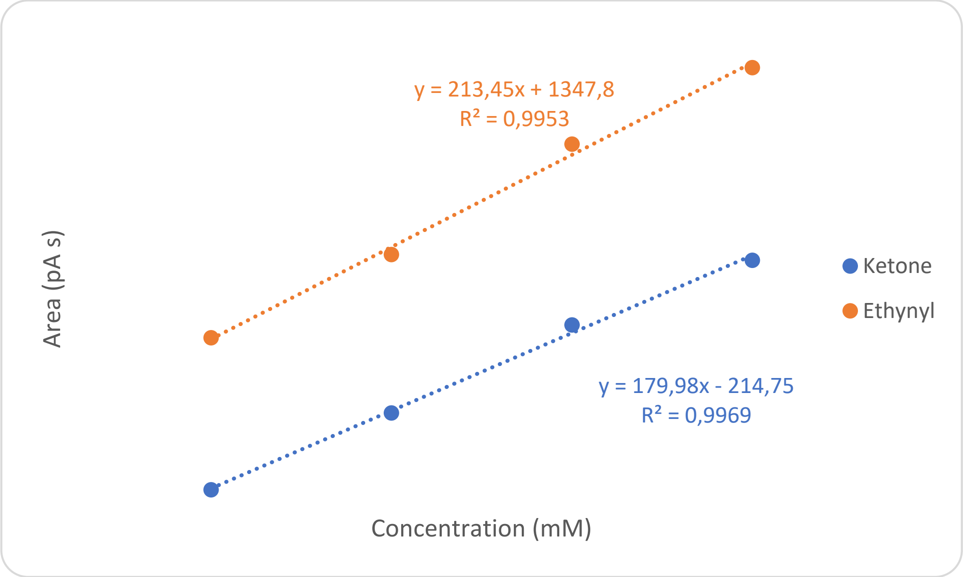

Correction factor = 1.186

VII.3.4. Calibration curve for conversion of 3-ethoxy-4-methoxyphenylacetylene (1f) into 1-(3-ethoxy-4-methoxyphenyl)-2-(methylsulfonyl)ethan-1-one (3h) at 228 nm wavelength

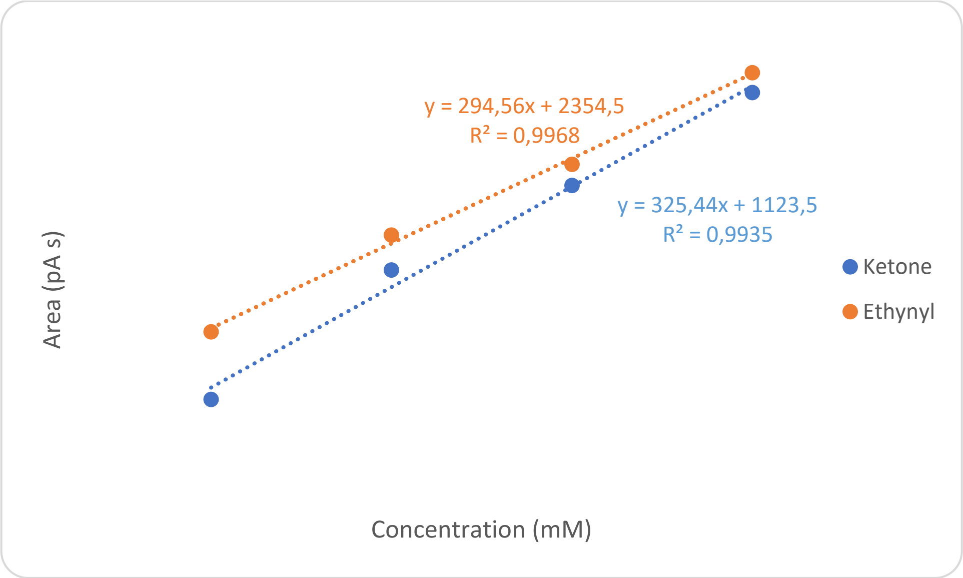

Correction factor = 1.250

VII.3.5. Calibration curve for conversion of 1-phenyl-2-(phenylsulfonyl)ethan-1-one (3a) into 1-phenyl-2-(phenylsulfonyl)ethan-1-ol (4a) at 210 nm wavelength

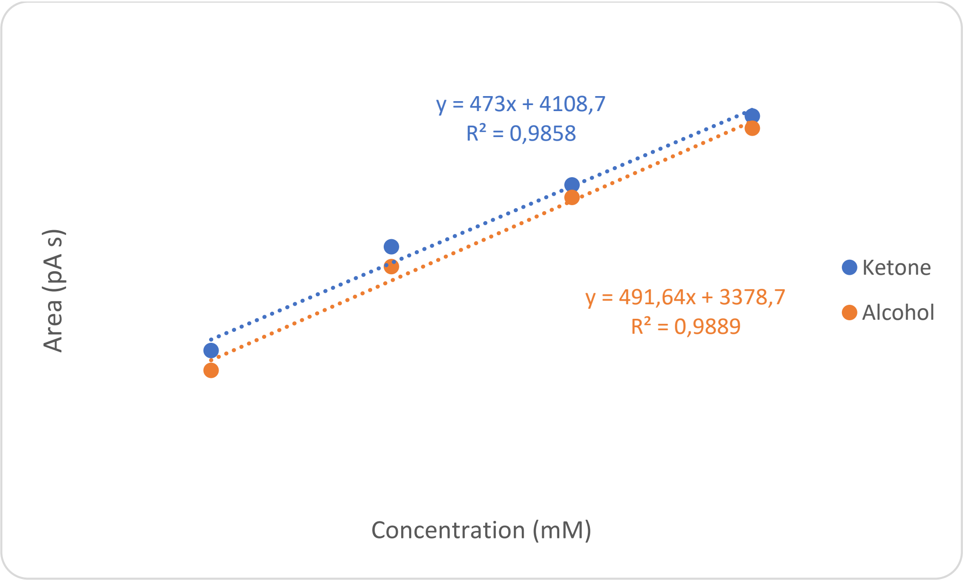

Correction factor = 1.039

VII.3.6. Calibration curve for conversion of 1-(4-methylphenyl)-2-(phenylsulfonyl)ethan-1-one (3b) into 1-(4-methylphenyl)-2-(phenylsulfonyl)ethan-1-ol (4b) at 210 nm wavelength

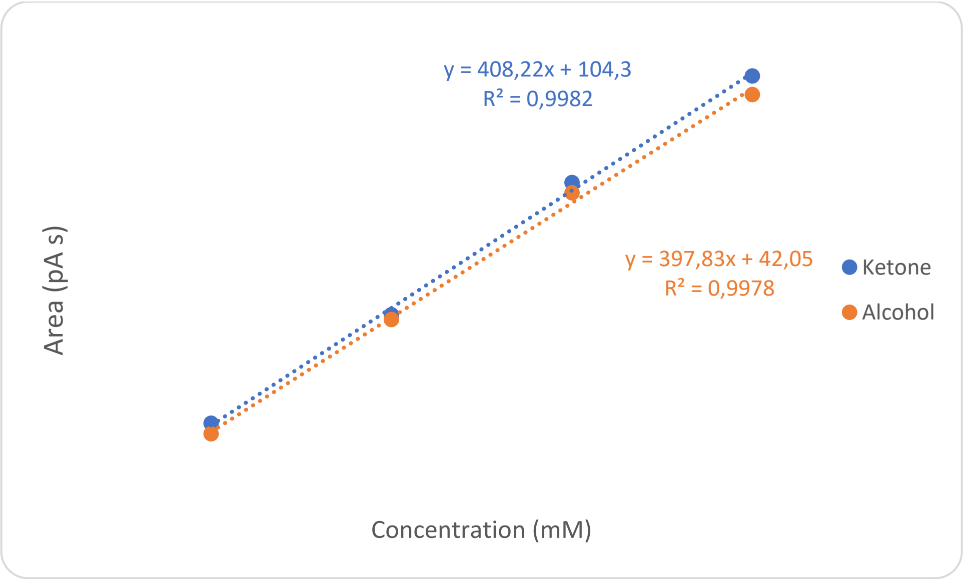

Correction factor = 1.026

VII.3.7. Calibration curve for conversion of 1-(4-methoxyphenyl)-2-(phenylsulfonyl)ethan-1-one (3c) into 1-(4-methoxyphenyl)-2-(phenylsulfonyl)ethan-1-ol (4c) at 210 nm wavelength

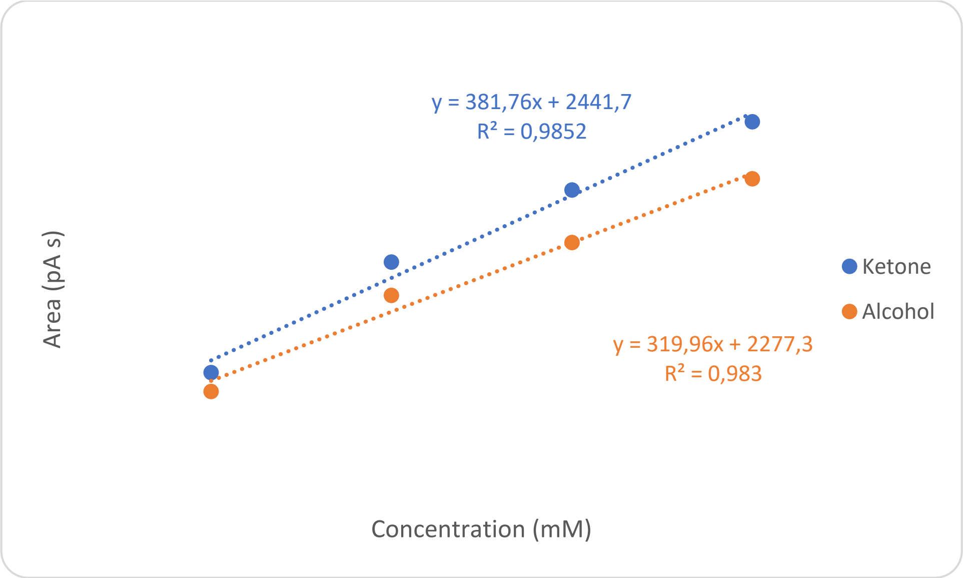

Correction factor = 1.190

VII.3.8. Calibration curve for conversion of 1-(4-bromophenyl)-2-(phenylsulfonyl)ethan-1-one (3d) into 1-(4-bromophenyl)-2-(phenylsulfonyl)ethan-1-ol (4d) at 210 nm wavelength

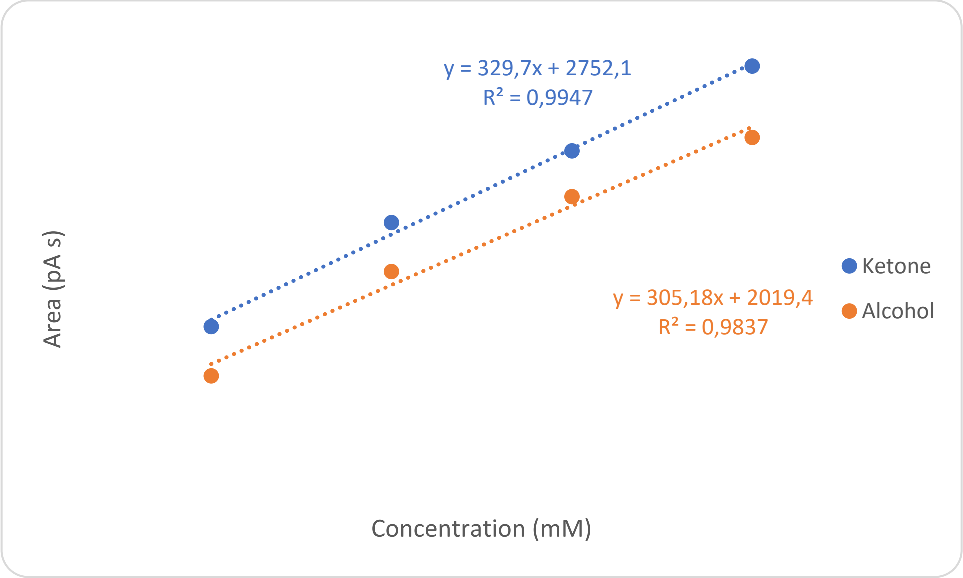

Correction factor = 1.080

VII.3.9. Calibration curve for conversion of 1-[(1,1'-biphenyl)-4-yl]-2-(phenylsulfonyl)ethan-1-one (3e) into 1-[(1,1'-biphenyl)-4-yl]-2-(phenylsulfonyl)ethan-1-ol (4e) at 210 nm wavelength

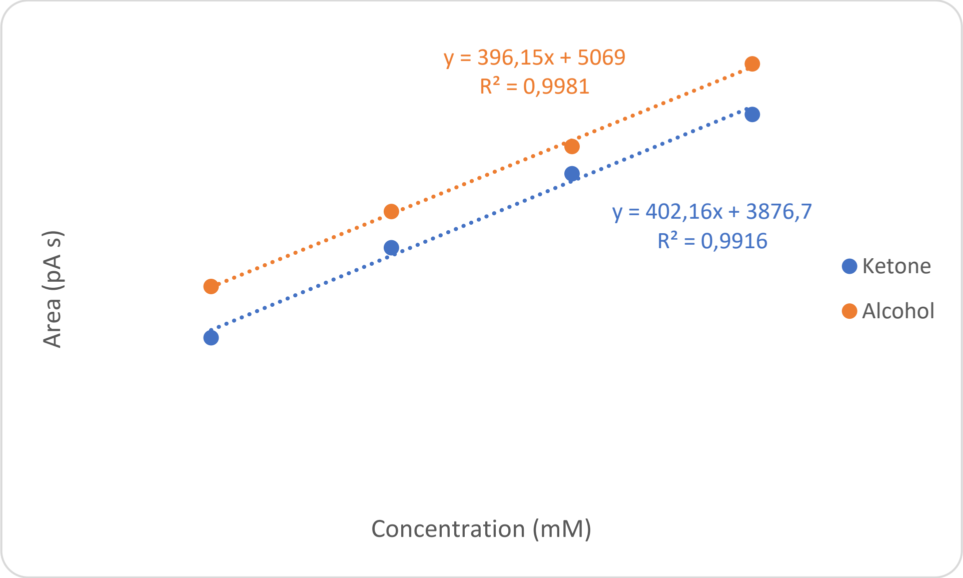

Correction factor = 1.015

VII.3.10. Calibration curve for conversion of 1-phenyl-2-tosylethan-1-one (3f) into 1-phenyl-2-tosylethan-1-ol (4f) at 210 nm wavelength

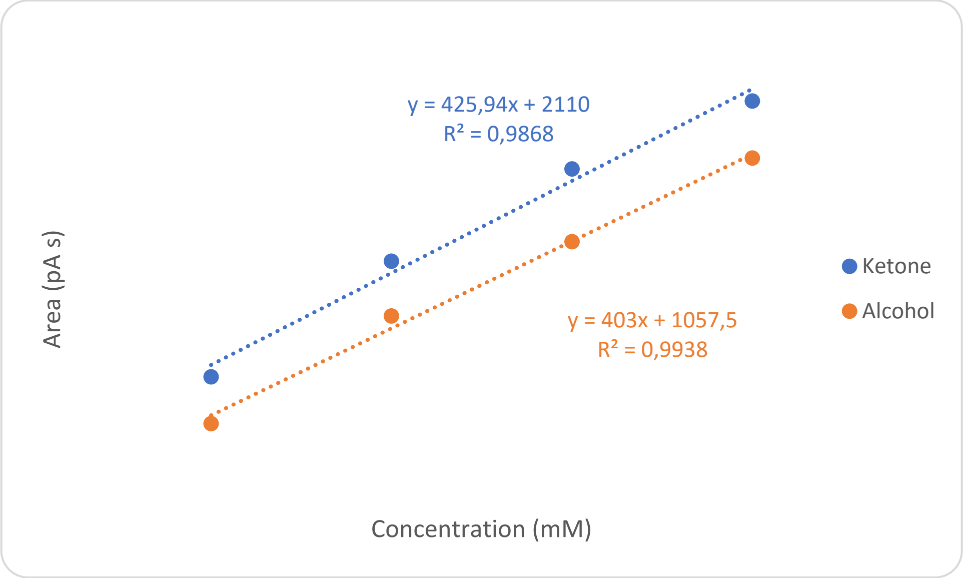

Correction factor = 1.057

VII.3.11. Calibration curve for conversion of 2-(methylsulfonyl)-1-phenylethan-1-one (3g) into 2-(methylsulfonyl)-1-phenylethan-1-ol (4g) at 210 nm wavelength

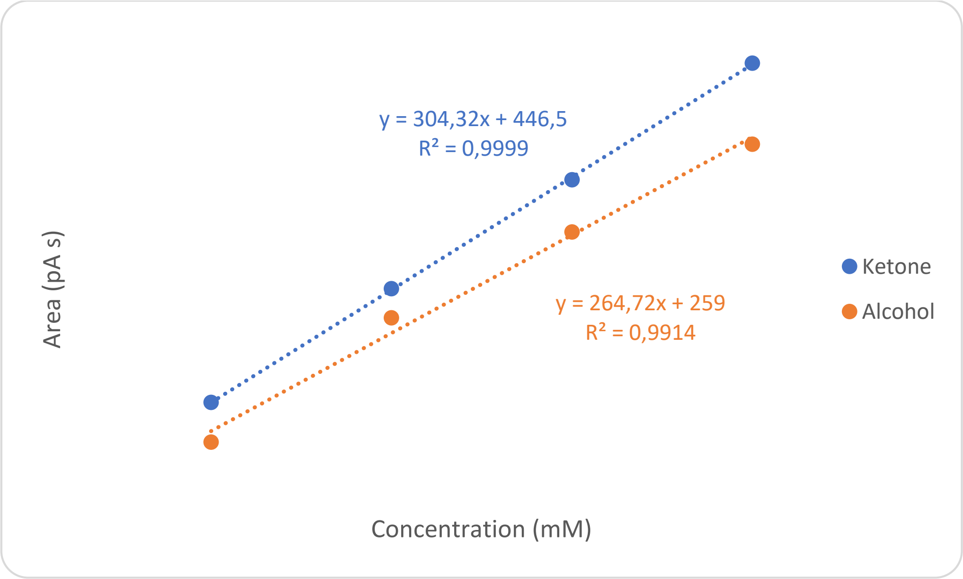

Correction factor = 1.150

VII.3.12. Calibration curve for conversion of into 1-(3-ethoxy-4-methoxyphenyl)-2-(methylsulfonyl)ethan-1-one (3h) into 1-(3-ethoxy-4-methoxyphenyl)-2-(methylsulfonyl)ethan-1-ol (4h) at 210 nm wavelength

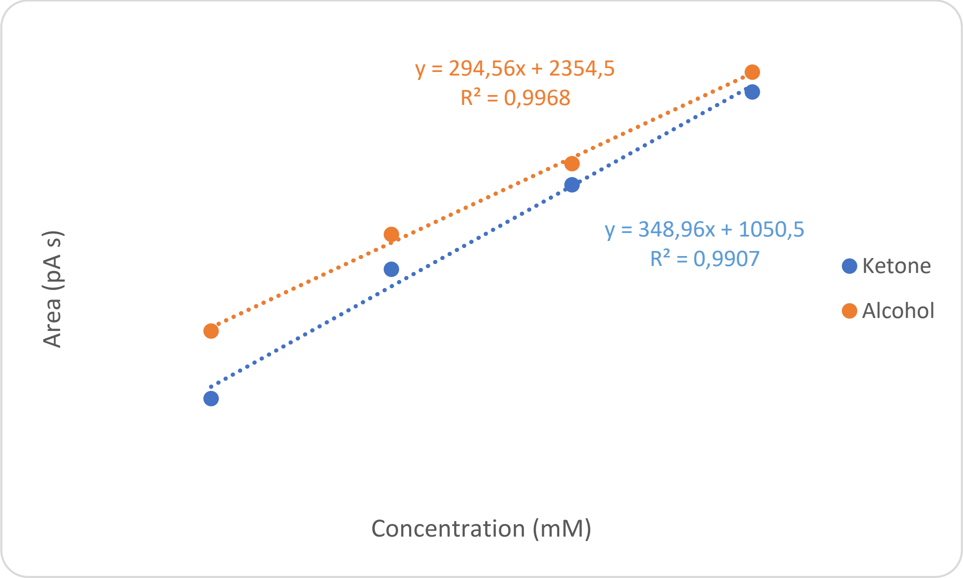

Correction factor = 1.180

VIII. HPLC chromatograms

VIII.1. Copy of HPLC chromatograms for racemic and enantioenriched 4a-h

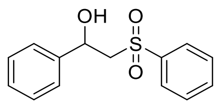

4a

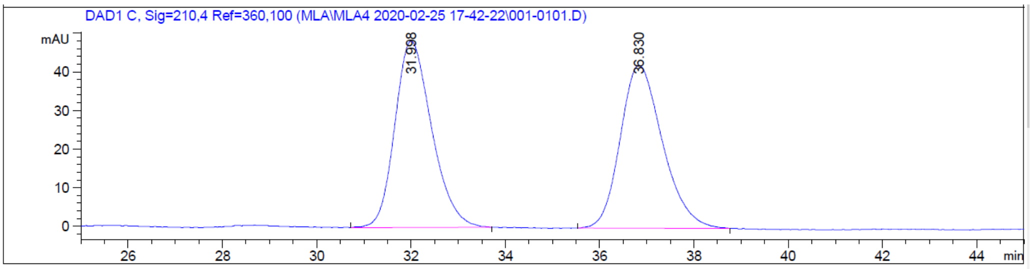

(S)-1-Phenyl-2-(phenylsulfonyl)ethan-1-ol **4a** (KRED-119, 98% *ee*)

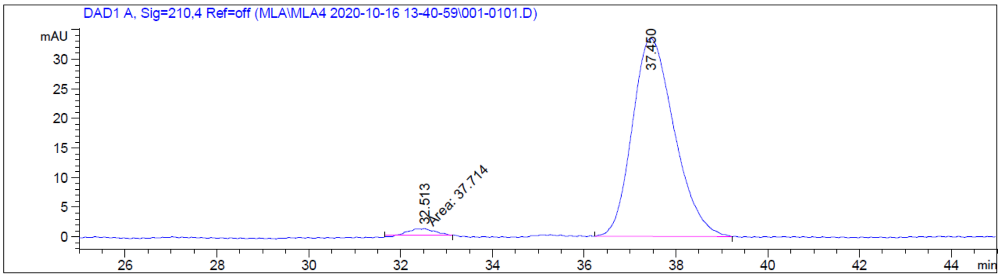

(R)-1-Phenyl-2-(phenylsulfonyl)ethan-1-ol **4a** (KRED-P1-B02, >99% *ee*)

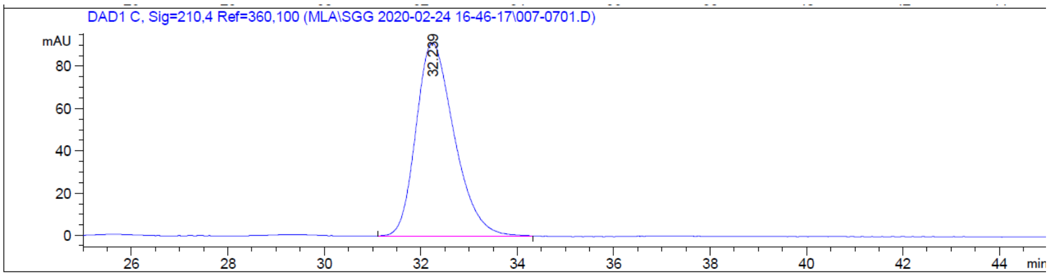

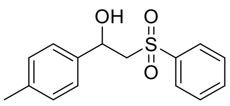

4b

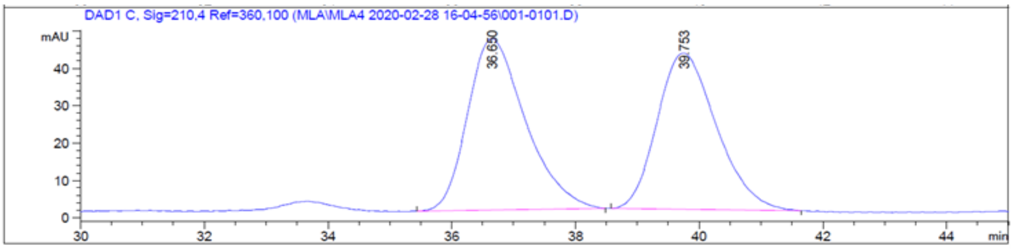

(S)-1-(4-Methylphenyl)-2-(phenylsulfonyl)ethan-1-ol **4b** (KRED-119, >99% *ee*)

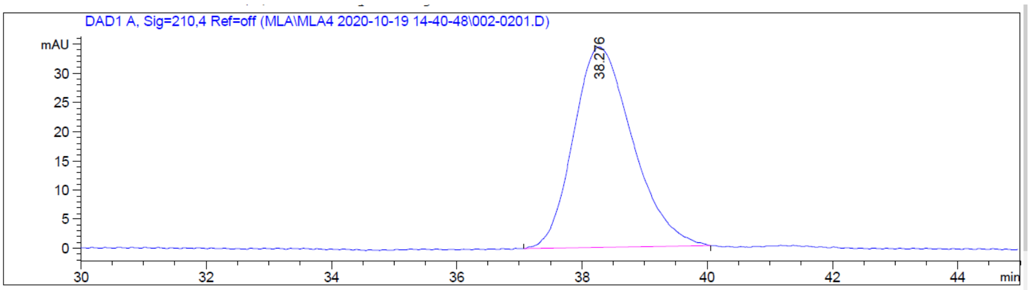

(R)-1-(4-Methylphenyl)-2-(phenylsulfonyl)ethan-1-ol **4b** (KRED-P1-B02, >99% *ee*)

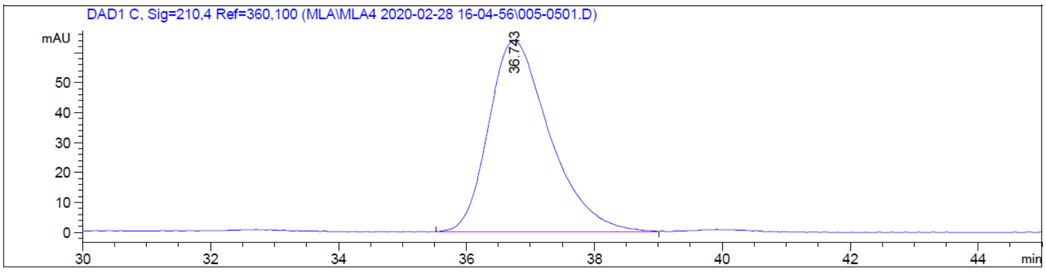

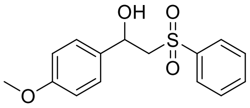

4c

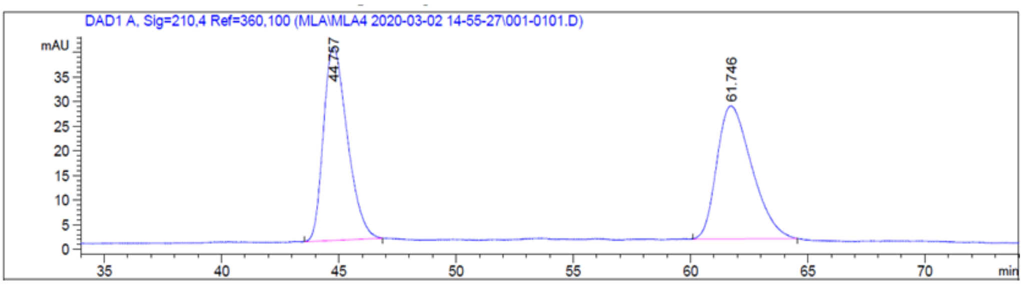

(S)-1-(4-Methoxyphenyl)-2-(phenylsulfonyl)ethan-1-ol **4c** (KRED-P1-B05, >99% *ee*)

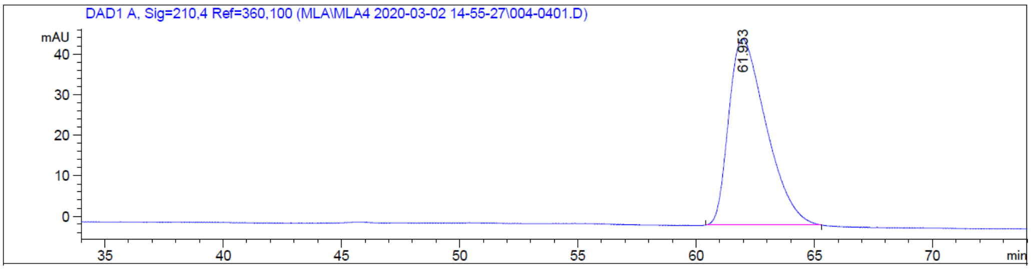

(R)-1-(4-Methoxyphenyl)-2-(phenylsulfonyl)ethan-1-ol **4c** (*Ras*ADH, >99% *ee*)

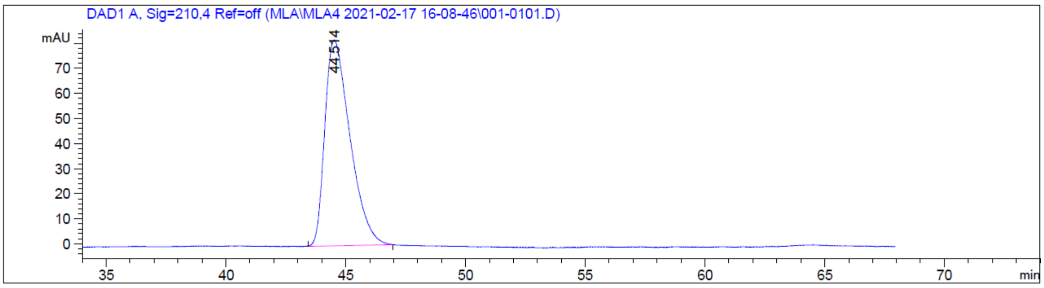

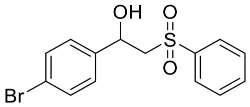

4d

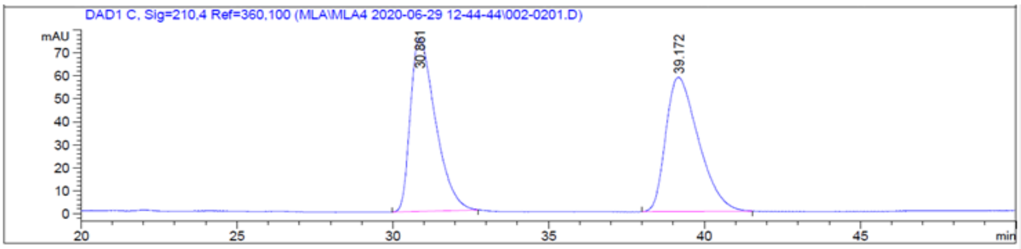

(S)-1-(4-Bromophenyl)-2-(phenylsulfonyl)ethan-1-ol **4d** (KRED-119, >99% *ee*)

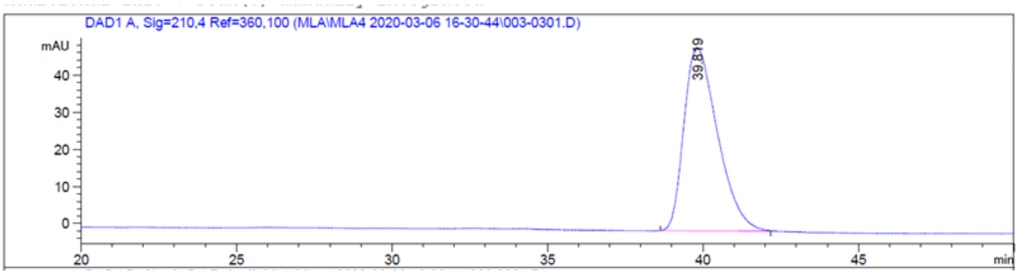

(R)-1-(4-Bromophenyl)-2-(phenylsulfonyl)ethan-1-ol **4d** (KRED-P1-B02, >99% *ee*)

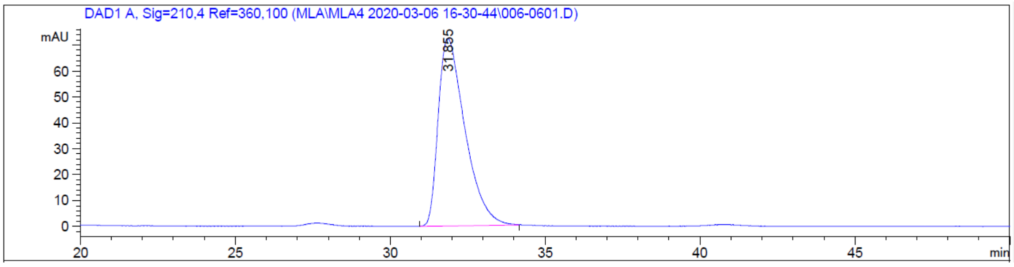

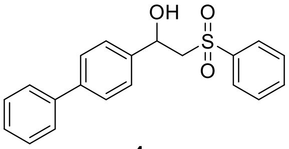

4e

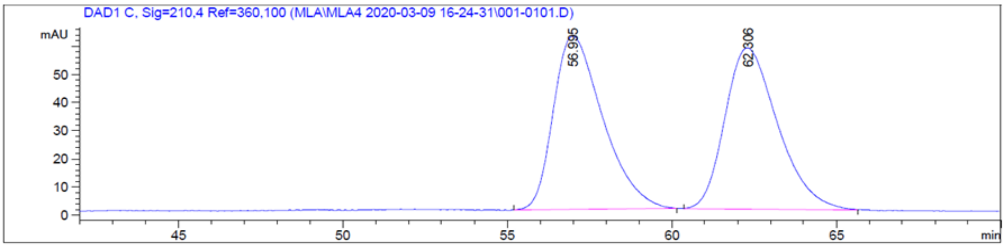

(S)-1-[(1,1'-Biphenyl)-4-yl]-2-(phenylsulfonyl)ethan-1-ol **4e** (KRED-P1-B05, >99% *ee*)

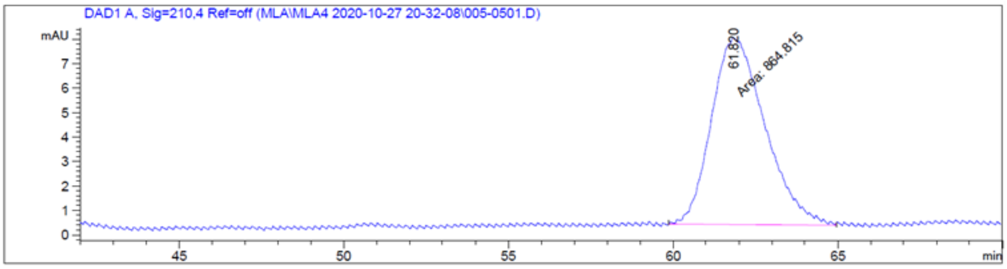

(R)-1-[(1,1'-Biphenyl)-4-yl]-2-(phenylsulfonyl)ethan-1-ol **4e** (KRED-130, >99% *ee*)

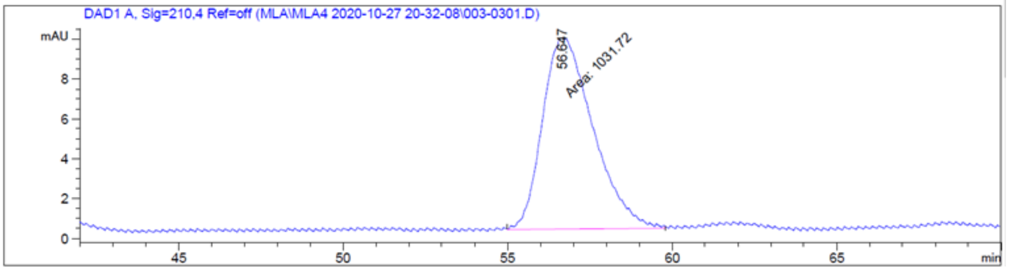

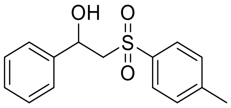

4f

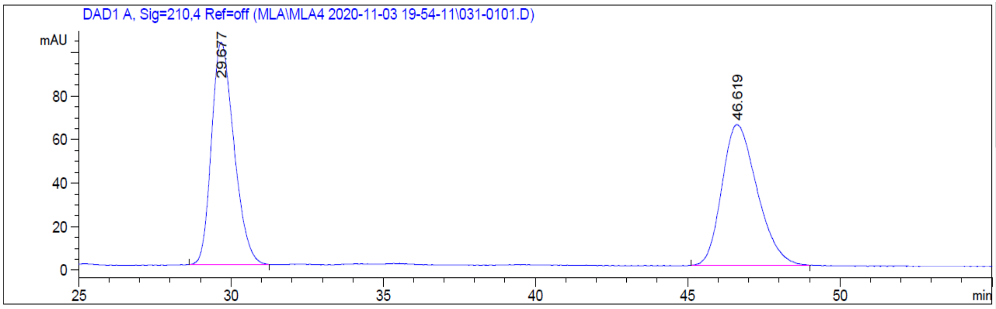

(S)-1-Phenyl-2-tosylethan-1-ol **4f** (KRED-119, 92% *ee*)

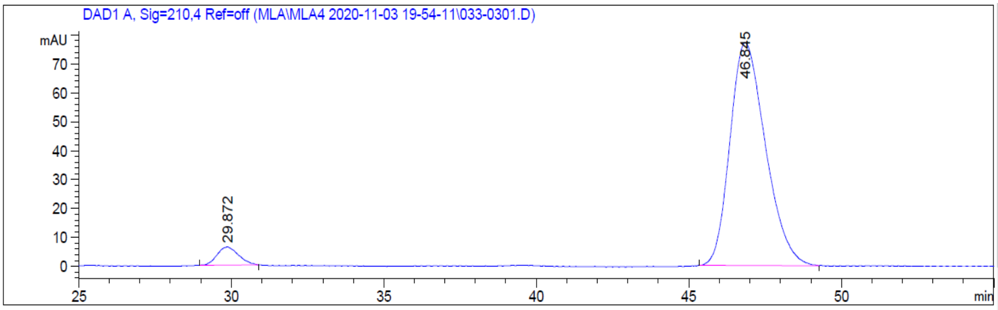

(R)-1-Phenyl-2-tosylethan-1-ol **4f** (KRED-P1-B02, >99% *ee*)

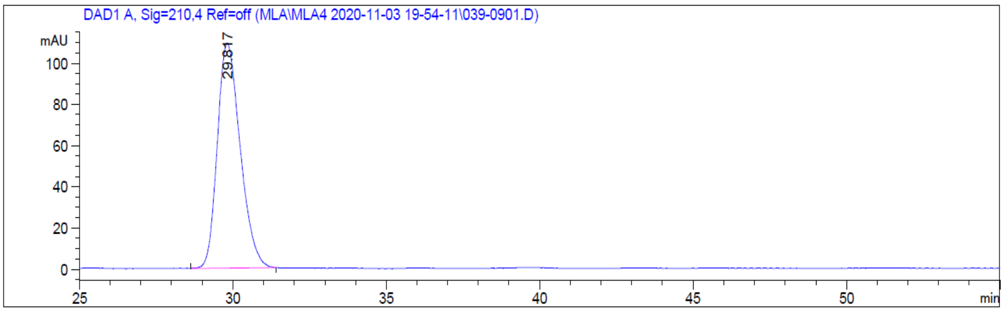

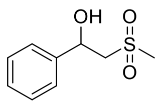

4g

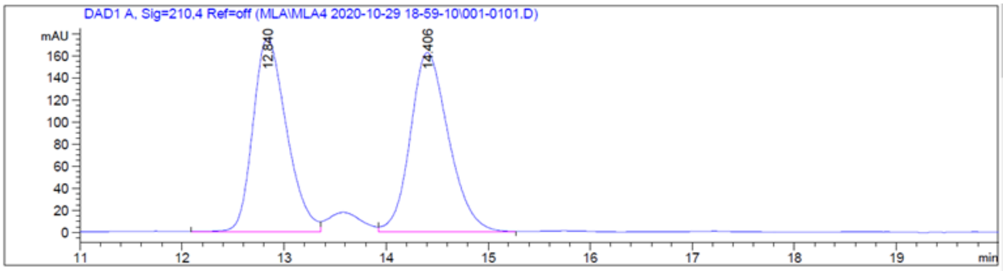

(S)-2-(Methylsulfonyl)-1-phenylethan-1-ol **4g** (KRED-119, >99% *ee*)

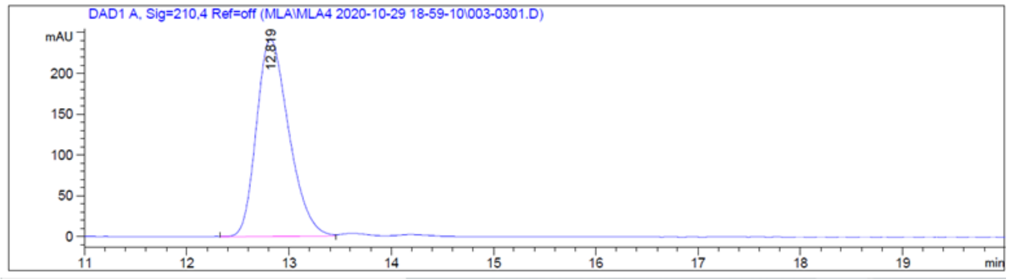

(R)-2-(Methylsulfonyl)-1-phenylethan-1-ol **4g** (KRED-P1-B02, >99% *ee*)

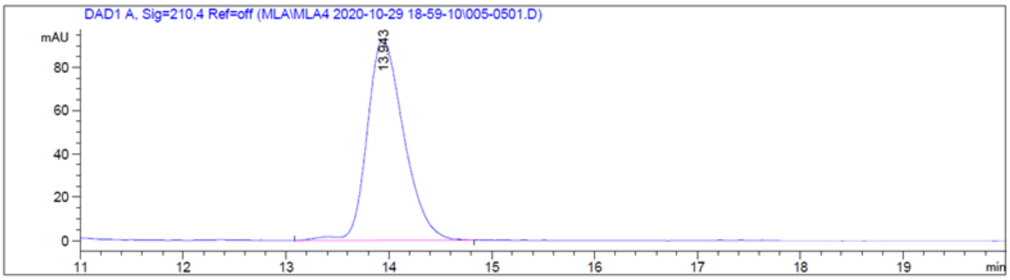

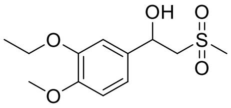

4h

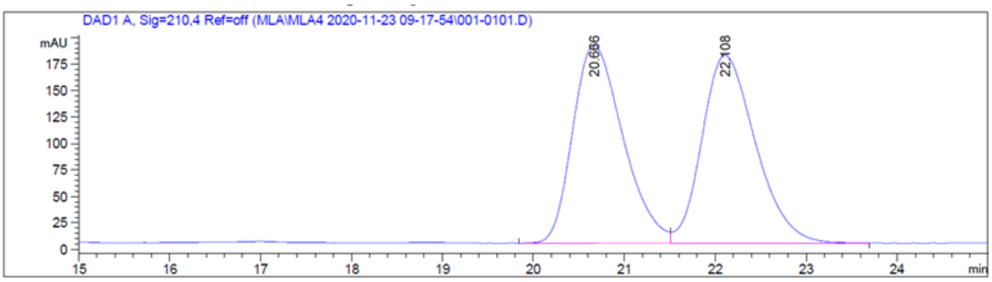

(S)-1-(3-Ethoxy-4-methoxyphenyl)-2-(methylsulfonyl)ethan-1-ol **4h** (KRED-P2-D12, 98% *ee*)

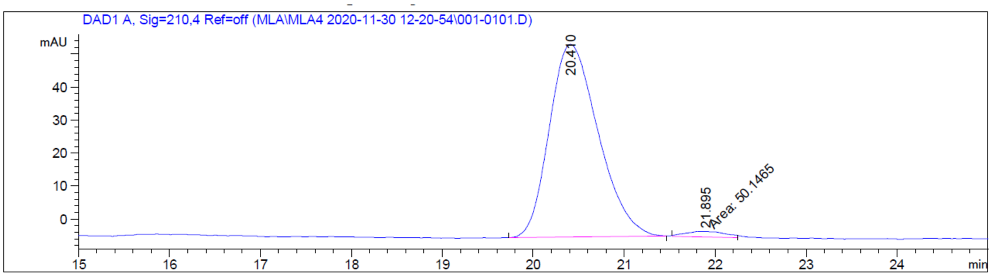

VIII.2. HPLC analysis for semi-preparative sequential transformation of 1a into 4a

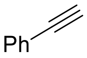

1a

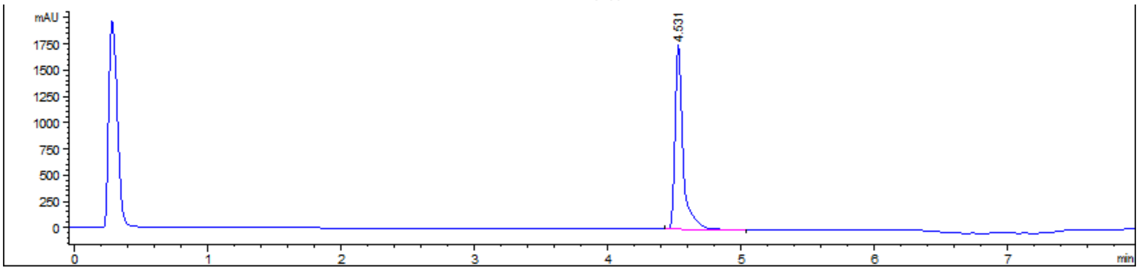

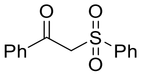

3a

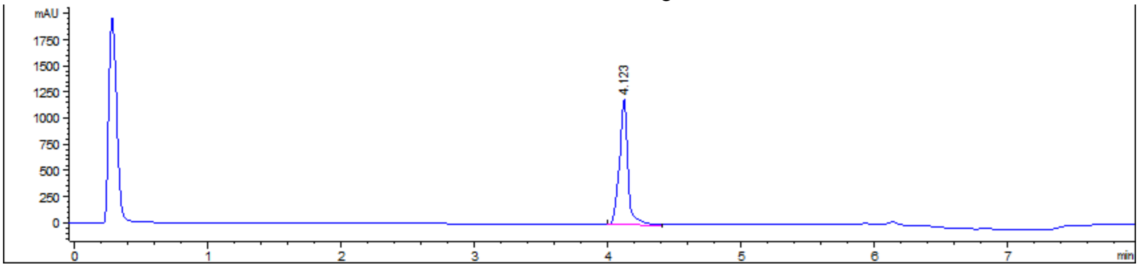

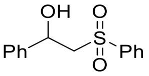

4a

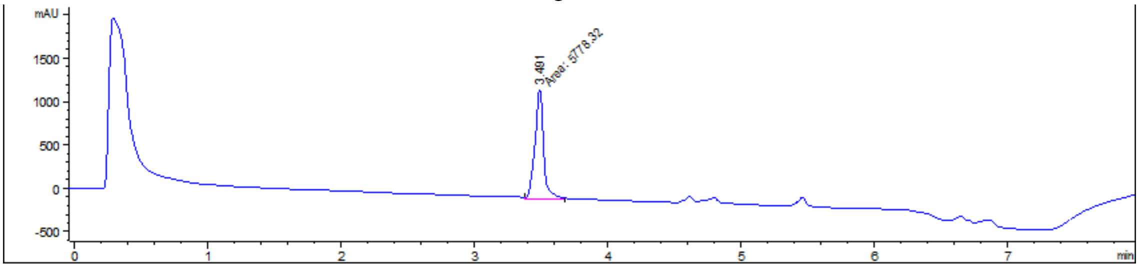

Sequential transformation:

1. $FeCl_3$-catalyzed oxosulfonylation step converting **1a** into alkyne **3a** (95% conversion)

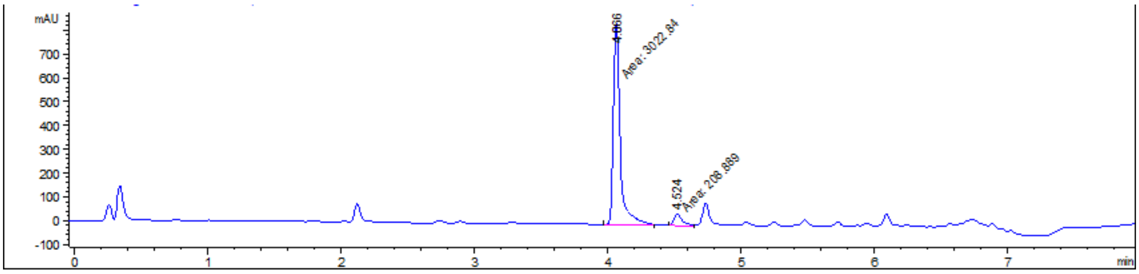

2. Bioreduction step catalyzed by KRED-P1-B02 (>99% conversion)

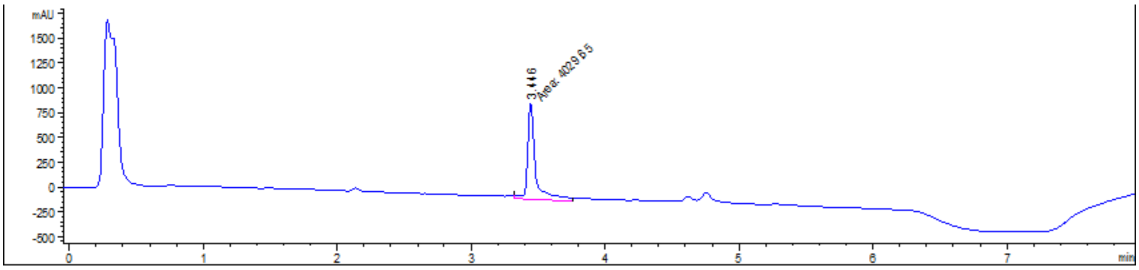

IX. Assignment of the absolute configuration for β-hydroxy sulfones 4a-h

The absolute configuration of alcohols **4a-d**,**g** was established by comparison of the sign of its specific rotation with the reported values (Table S14):

- For 1-phenyl-2-(phenylsulfonyl)ethan-1-ol (**4a**) by Node *et al.*[14]
- For 1-(4-methylphenyl)-2-(phenylsulfonyl)ethan-1-ol (**4b**), 1-(4-methoxyphenyl)-2-(phenylsulfonyl)ethan-1-ol (**4c**), 1-(4-bromophenyl)-2-(phenylsulfonyl)ethan-1-ol (**4d**), and 2-(methylsulfonyl)-1-phenylethan-1-ol (**4g**) by Tao *et al.*[15]
- For 1-(3-ethoxy-4-methoxyphenyl)-2-(methylsulfonyl)ethan-1-ol (**4h**) by Vega *et al.*[16]

Table S14. Optical rotation values reported for β-hydroxy sulfones **4a-h**.[a]

**β-Hydroxy sulfone**	**Experimental** $[\alpha]_D^{20}$	**Literature** $[\alpha]_D^{25}$[14-16]
(*R*)-**4a**	–27.0 (*c* 1.0, $CHCl_3$) for >99% *ee*[b]	–30.3 (*c* 1.0, $CHCl_3$), >99% *ee*
(*S*)-**4b**	+12.8 (c 1.0, $CHCl_3$) for >99% *ee*[c]	+18.5 (*c* 1.5, $CHCl_3$), 96% *ee*
(*S*)-**4c**	+13.8 (c 1.5, $CHCl_3$) for >99% *ee*[d]	+13.9 (*c* 1.5, $CHCl_3$), 98% *ee*
(*S*)-**4d**	+13.0 (c 1.0, $CHCl_3$) for >99% *ee*[d]	+24.5 (*c* 1.5, $CHCl_3$), 94% *ee*
(*S*)-**4e**	+12.2 (c 1.0, $CHCl_3$) for >99% *ee*[c]	Not described
(*R*)-**4f**	–16.1 (*c* 1.0, $CHCl_3$) for >99% *ee*[e]	Not described
(*R*)-**4g**	–29.0 (c 1.0, $CHCl_3$) for >99% *ee*[e]	+49.9 (*c* 1.5, $CHCl_3$), 97% *ee* for the (*S*)-enantiomer
(*R*)-**4h**	–33.4 (*c* 1.0, $CHCl_3$) for 79% *ee*[b]	–22.0 (*c* 0.65, EtOAc), >99% *ee* for the (*R*)-enantiomer

[a] Measurement of the β-hydroxy sulfones optical rotation values was made after column chromatography purification of the corresponding reaction crudes. [b] After bioreduction with *Ras*ADH. [c] After bioreduction with KRED-119. [d] After bioreduction with KRED-P1-B05. [e] After bioreduction with KRED-P1-B10.

The absolute configurations of 1-[(1,1'-biphenyl)-4-yl]-2-(phenylsulfonyl)ethan-1-ol (**4e**) and 1-phenyl-2-tosylethan-1-ol (**4f**) were established after comparison of the order of elution of the enantiomers with the model substrate **4a** under identical HPLC conditions, which also matched with the predicted stereopreference for the enzymes based on the bioreductions with the other similar β-keto sulfones.

The absolute configuration of 1-(3-ethoxy-4-methoxyphenyl)-2-(methylsulfonyl)ethan-1-ol (**4h**) was also established by comparison with the elution order of the enantiomers with the data reported by Ruchelman and Connolly.[17]

X. References

[1] H. Man, K. Kędziora, J. Kulig, A. Frank, I. Lavandera, V. Gotor-Fernández, D. Rother, S. Hart, J. P. Turkenburg, G. Grogan, *Top. Catal.* **2014**, *57*, 356-365.

[2] I. Lavandera, A. Kern, V. Resch, B. Ferreira-Silva, A. Glieder, W. M. F. Fabian, S. de Wildeman, W. Kroutil, *Org. Lett.* **2008**, *10*, 2155-2158.

[3] J. Peters, T. Minuth, M.-R. Kula, *Enzyme Microb. Technol.* **1993**, *11*, 950-958.

[4] C. Heiss, M. Laivenieks, J. G. Zeikus, R. S. Phillips, *Bioorg. Med. Chem.* **2001**, *9*, 1659-1666.

[5] W. Stampfer, B. Kosjek, C. Moitzi, W. Kroutil, K. Faber, *Angew. Chem. Int. Ed.* **2002**, *41*, 1014-1017.

[6] S. Leuchs, L. Greiner, *Chem. Biochem. Eng. Q.* **2011**, *25*, 267-281.

[7] a) C. W. Bradshaw, W. Hummel, C. H. Wong, *J. Org. Chem.* **1992**, *57*, 1532-1536; b) A. Weckbecker, W. Hummel, *Biocatal. Biotransform.* **2006**, *24*, 380-389.

[8] A. Rosiak, W. Frey, J. Christoffers, *Eur. J. Org. Chem.* **2006**, 4044-4054.

[9] a) M. Eissen, J. O. Metzger, *Chem. Eur. J.* **2002**, *8*, 3580-3585; b) EATOS: Environmental Assessment Tool for Organic Syntheses, http://www.metzger.chemie.uni-oldenburg.de/eatos/english.htm.

[10] The excel file to show EATOS results in Figures S4-S7 was adapted using a template provided in: J. H. Schrittwieser, F. Coccia, S. Kara, B. Grischek, W. Kroutil, N. d'Alessandro, F. Hollmann, *Green Chem.* **2013**, *15*, 3318-3331.

[11] D. Zhang, T. Cheng, Q. Zhao, J. Xu, G. Liu, *Org. Lett.* **2014**, *16*, 5764-5767.

[12] P. Cui, Q. Liu, J. Wang, H. Liu, H. Zhou, *Green Chem.* **2019**, *21*, 634-639.

[13] S. Wang, C. Wang, N. Lv, C. Tan, T. Cheng, G. Liu, *ChemCatChem* **2021**, *13*, 909-915.

[14] M. Node, K. Nishide, Y. Shigeta, K. Obata, H. Shiraki, H. Kunishige, *Tetrahedron* **1997**, *53*, 12883-12894.

[15] L. Tao, C. Yin, X.-Q. Dong, X. Zhang, *Org. Biomol. Chem.* **2019**, *17*, 785-788.

[16] K. B. Vega, D. M. V. Cruz, A. R. T. Oliveira, M. R. da Silva, T. L. G. de Lemos, M. C. F. Oliveira, R. D. S. Bernardo, J. R. de Sousa, G. Zanatta, F. D. Nasário, A. J. Marsaioli, M. C. de Mattos, *J. Braz. Chem. Soc.* **2021**, *32*, 1100-1110.

[17] A. L. Ruchelman, T. J. Connolly, *Tetrahedron: Asymmetry* **2015**, *26*, 553-559.

XI. NMR spectra

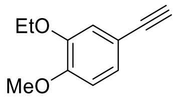

1f

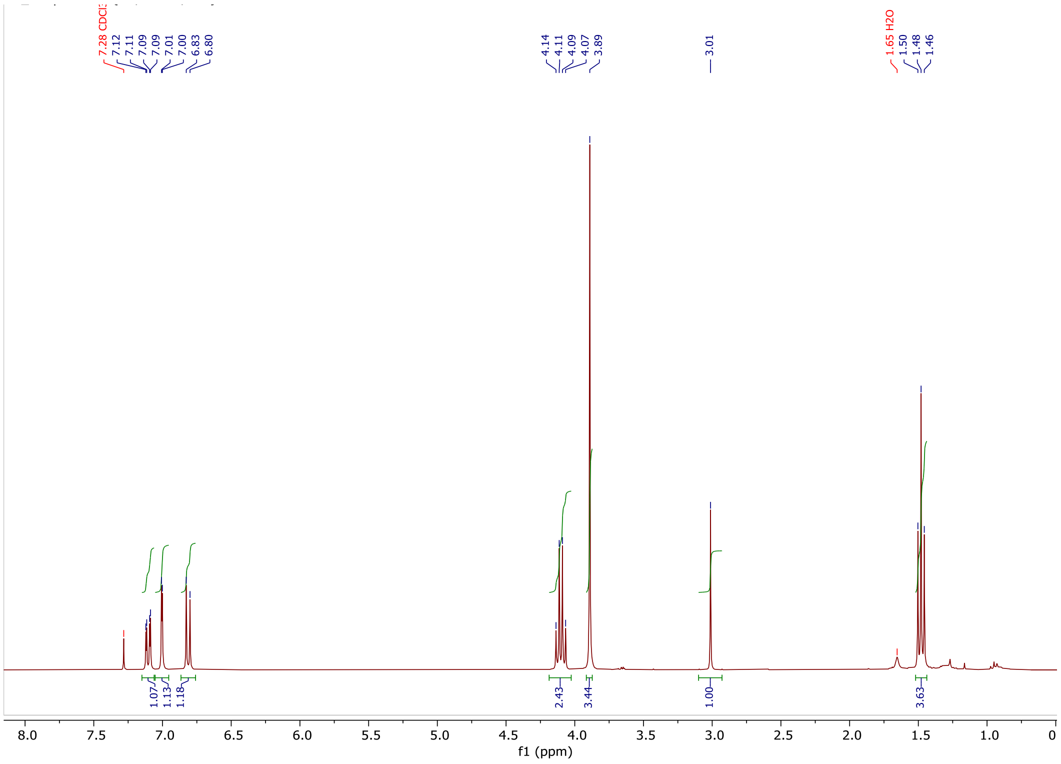

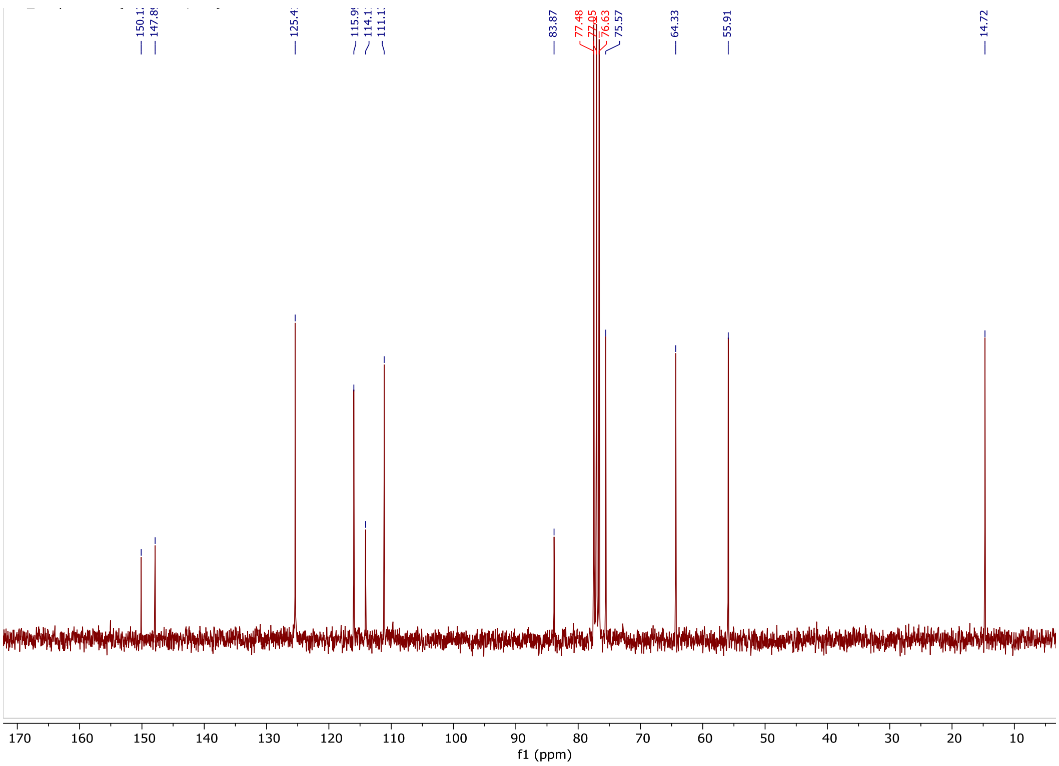
150.1
147.8
125.4
115.9
114.1
111.1
83.87
77.48
77.05
76.63
75.57
64.33
55.91
14.72
170 160 150 140 130 120 110 100 90 80 70 60 50 40 30 20 10
f1 (ppm)

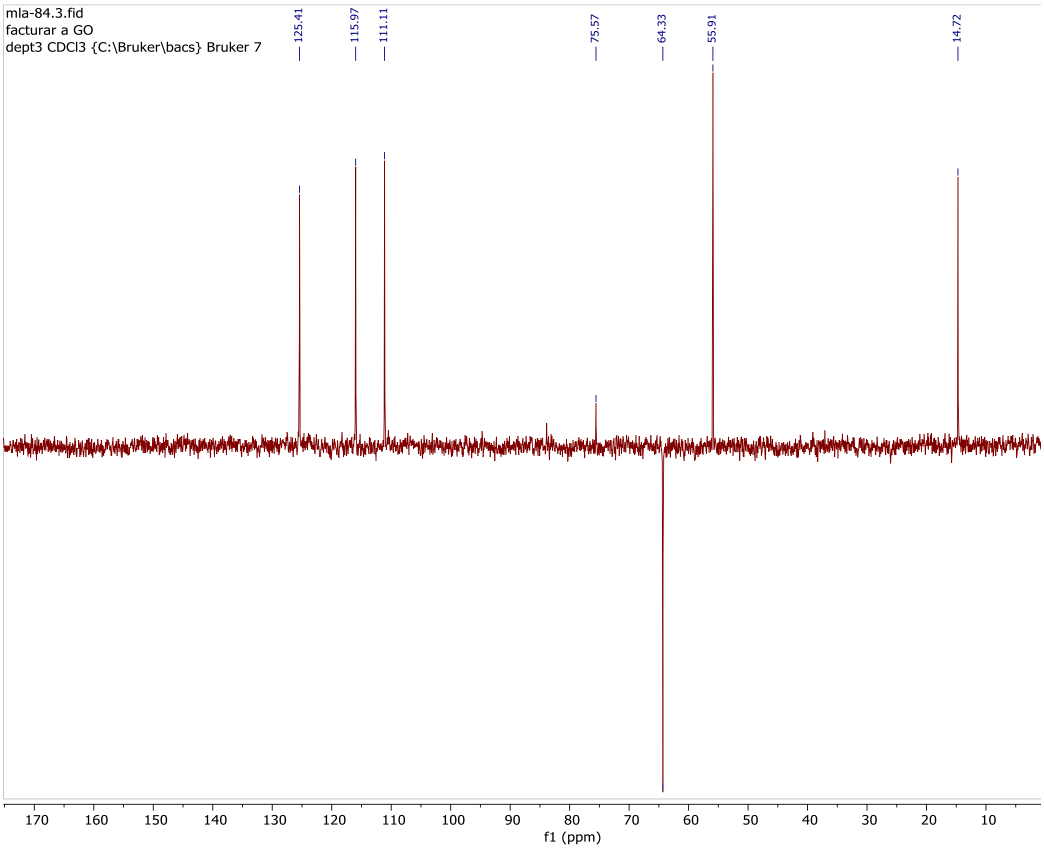
mla-84.3.fid
facturar a GO
dept3 CDCl3 {C:\Bruker\bacs} Bruker 7
125.41
115.97
111.11
75.57
64.33
55.91
14.72
170 160 150 140 130 120 110 100 90 80 70 60 50 40 30 20 10
f1 (ppm)

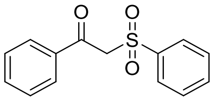
O
O
S
O

3a

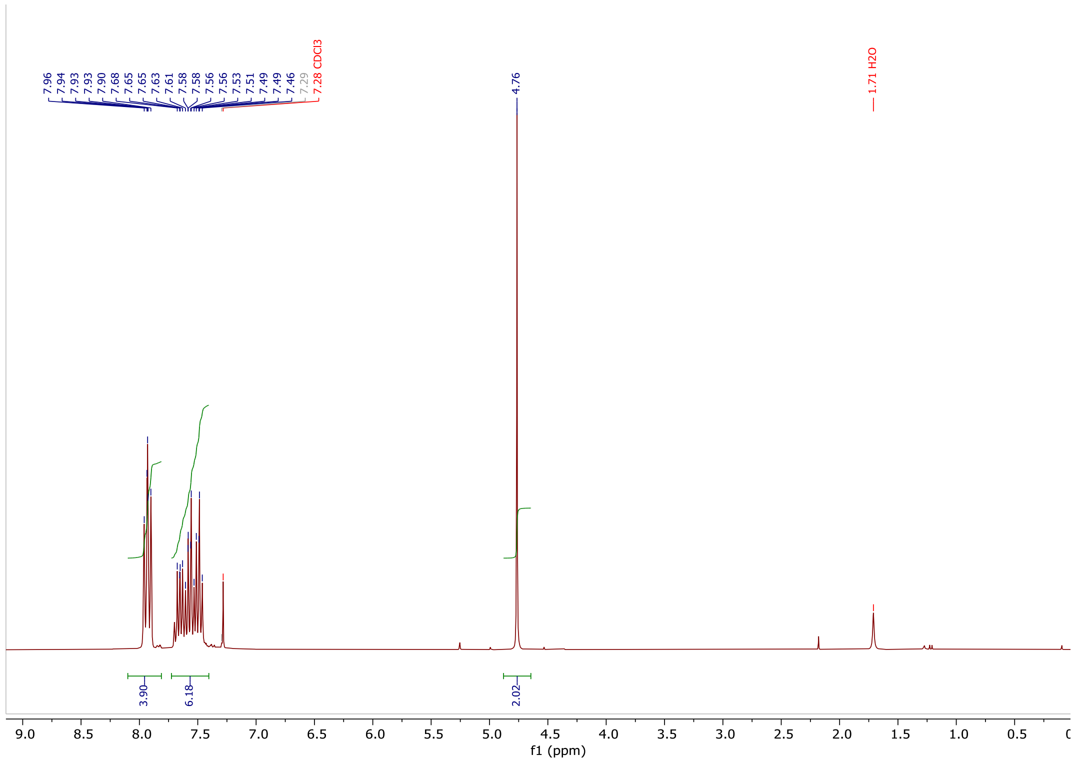
7.96
7.94
7.93
7.93
7.90
7.68
7.65
7.65
7.63
7.61
7.58
7.58
7.56
7.56
7.53
7.51
7.49
7.49
7.46
7.29
7.28 CDCl3
4.76
1.71 H2O
3.90
6.18
2.02
9.0
8.5
8.0
7.5
7.0
6.5
6.0
5.5
5.0
4.5
4.0
3.5
3.0
2.5
2.0
1.5
1.0
0.5
f1 (ppm)

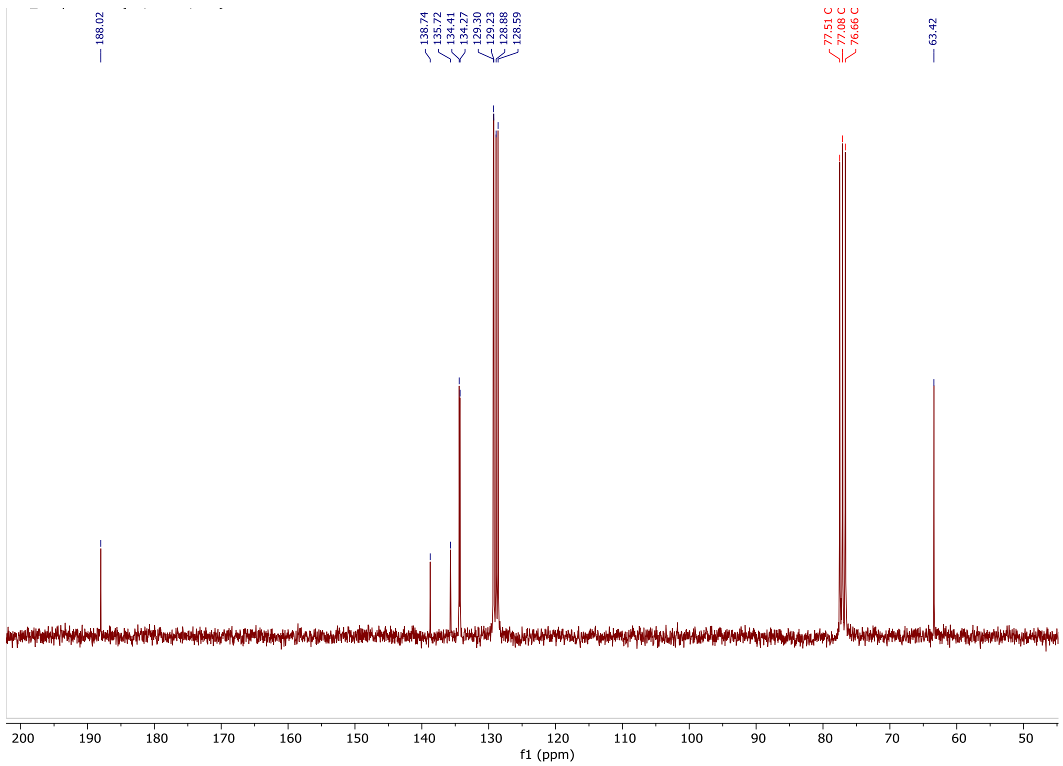
188.02
138.74
135.72
134.41
134.27
129.30
129.23
128.88
128.59
77.51 C
77.08 C
76.66 C
63.42
200 190 180 170 160 150 140 130 120 110 100 90 80 70 60 50
f1 (ppm)

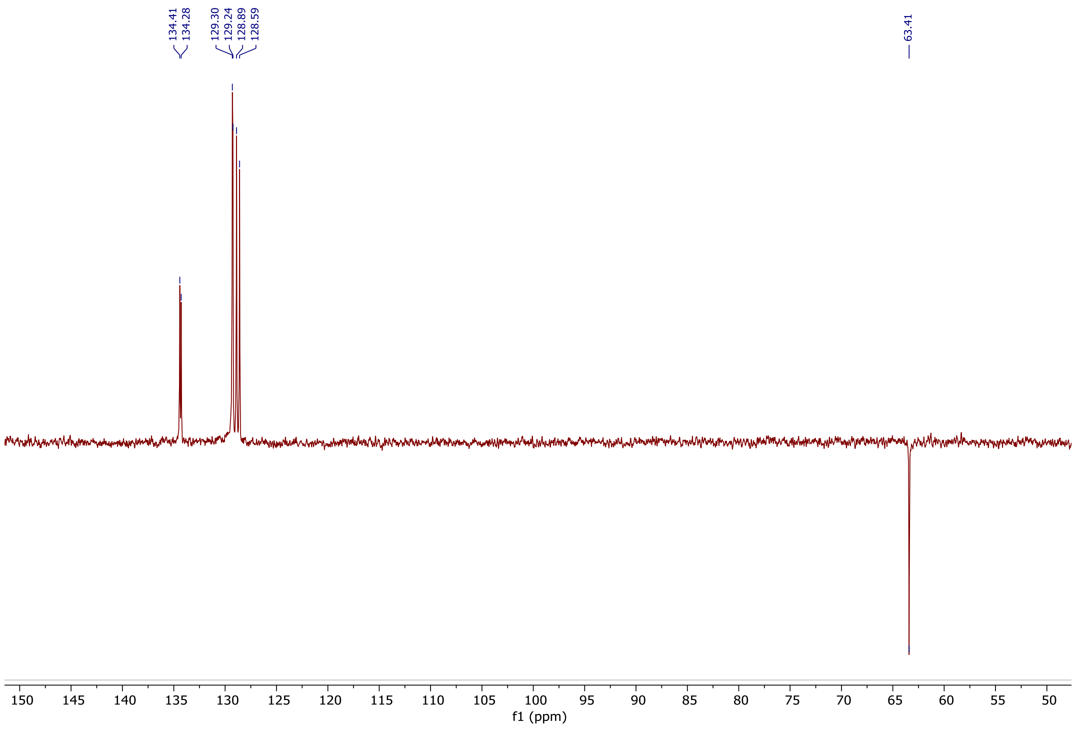
134.41
134.28
129.30
129.24
128.89
128.59
63.41
150 145 140 135 130 125 120 115 110 105 100 95 90 85 80 75 70 65 60 55 50
f1 (ppm)

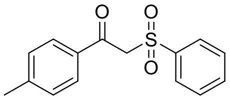

3b

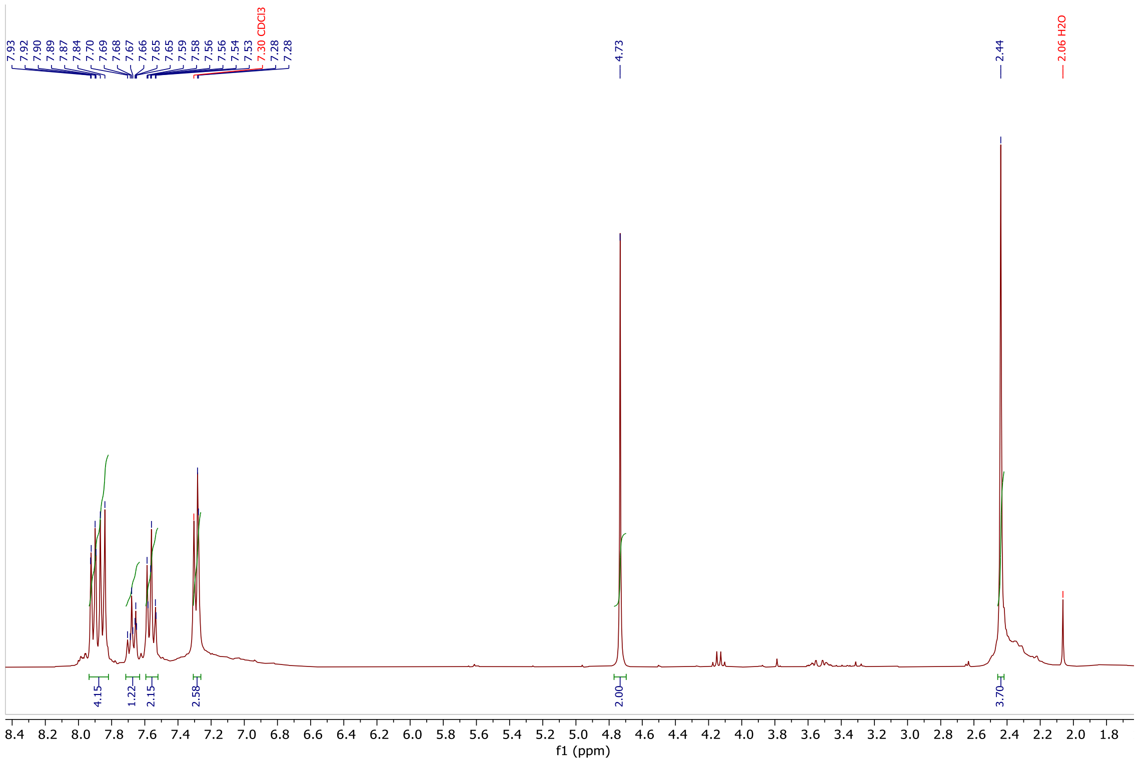

7.93
7.92
7.90
7.89
7.87
7.84
7.70
7.69
7.68
7.67
7.66
7.65
7.65
7.59
7.58
7.56
7.56
7.54
7.53
7.30 CDCl3
7.28
7.28
4.73
2.44
2.06 H2O
4.15
1.22
2.15
2.58
2.00
3.70
f1 (ppm)

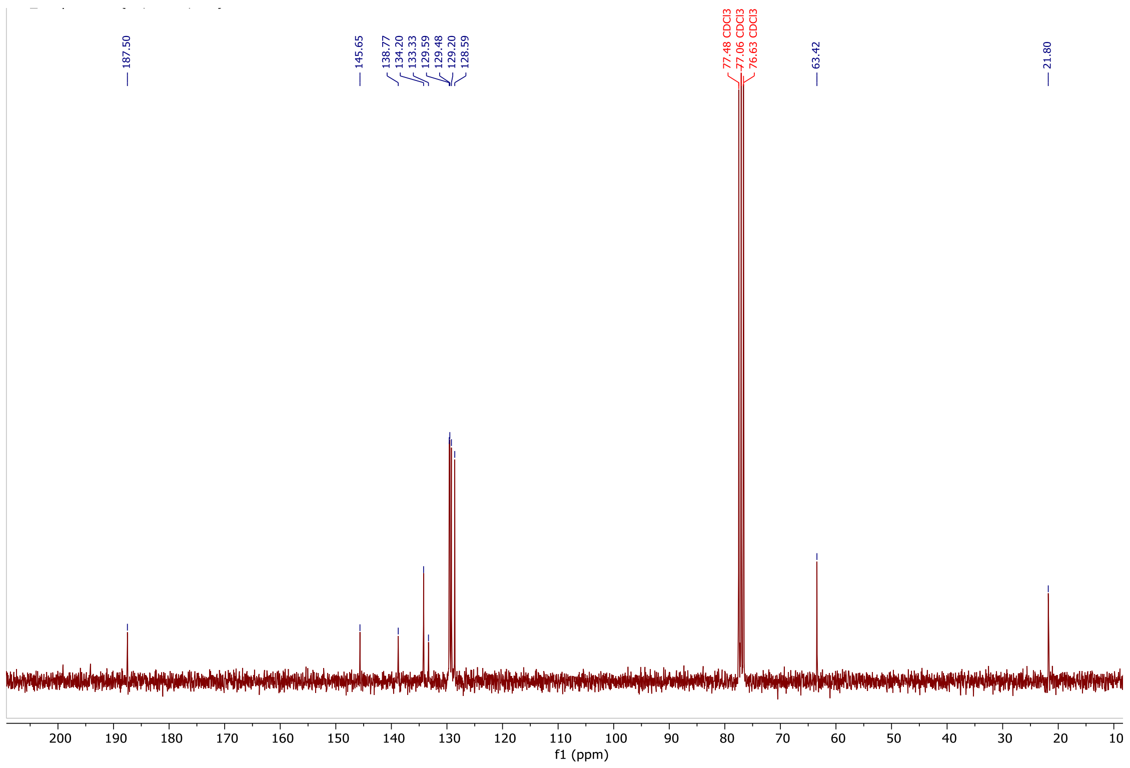
187.50
145.65
138.77
134.20
133.33
129.59
129.48
129.20
128.59
77.48 CDCl3
77.06 CDCl3
76.63 CDCl3
63.42
21.80
200 190 180 170 160 150 140 130 120 110 100 90 80 70 60 50 40 30 20 10
f1 (ppm)

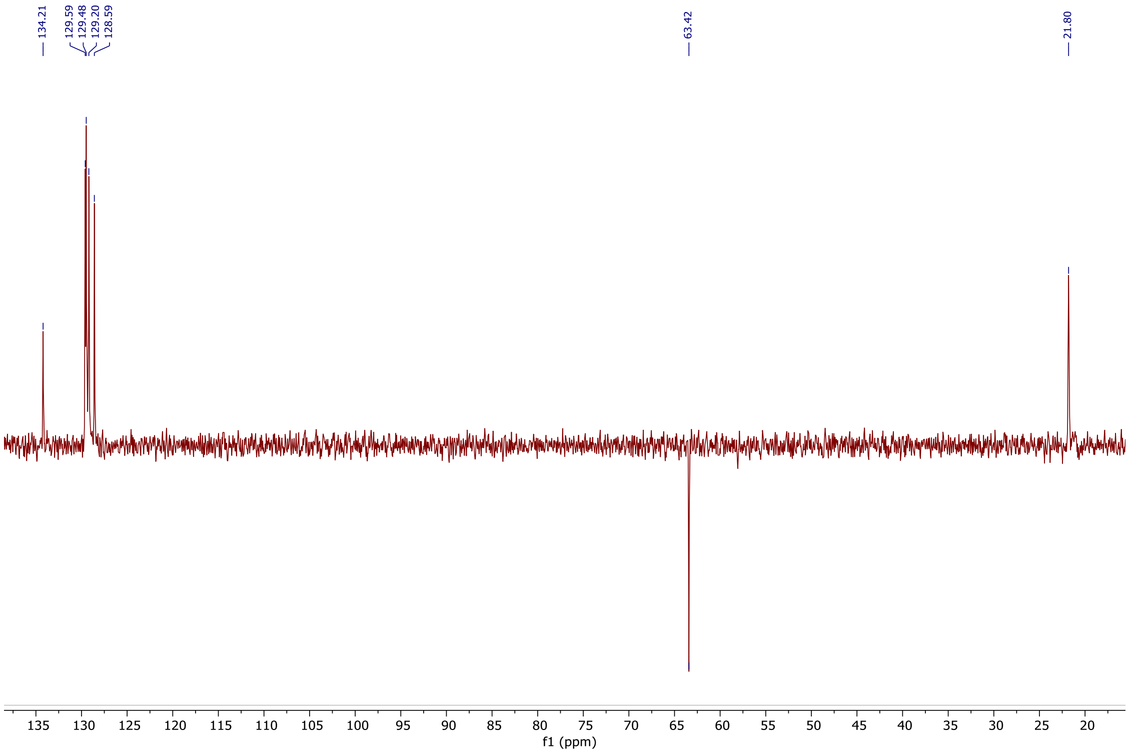
134.21
129.59
129.48
129.20
128.59
63.42
21.80
135 130 125 120 115 110 105 100 95 90 85 80 75 70 65 60 55 50 45 40 35 30 25 20
f1 (ppm)

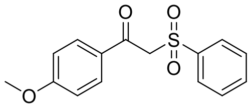
O
O
S
O
O

3c

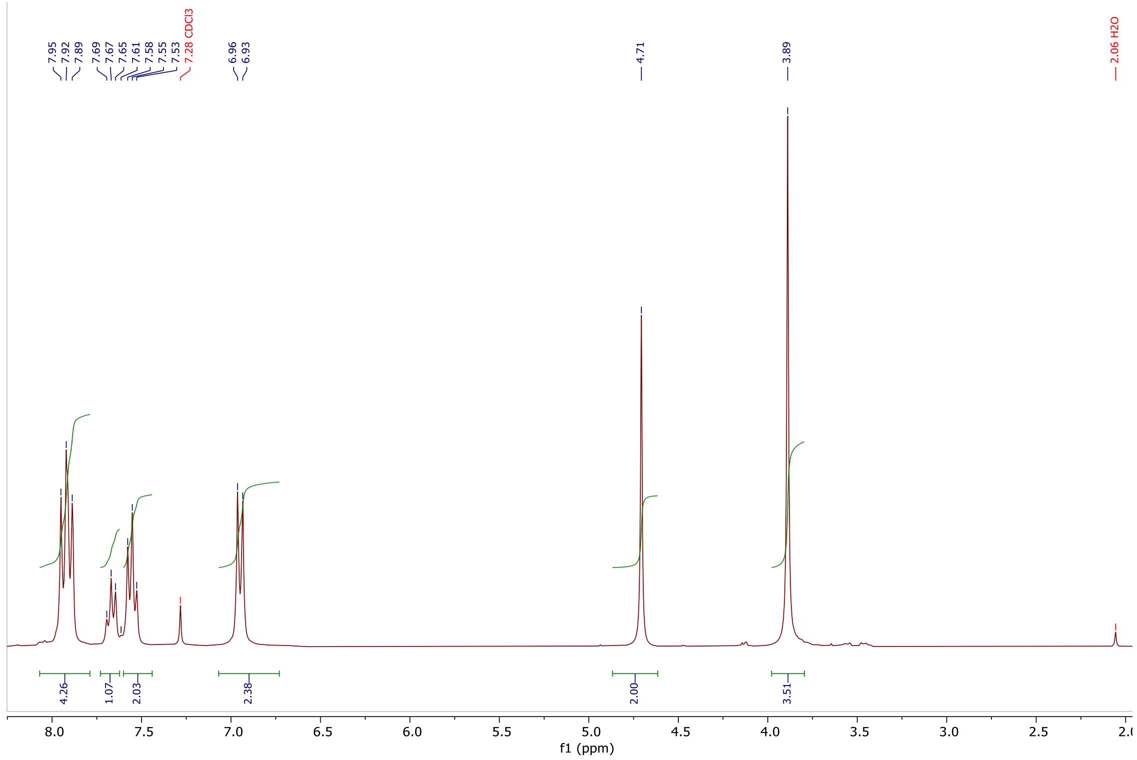
7.95
7.92
7.89
7.69
7.67
7.65
7.61
7.58
7.55
7.53
7.28 CDCl3
6.96
6.93
4.71
3.89
2.06 H2O
4.26
1.07
2.03
2.38
2.00
3.51
8.0
7.5
7.0
6.5
6.0
5.5
5.0
4.5
4.0
3.5
3.0
2.5
f1 (ppm)

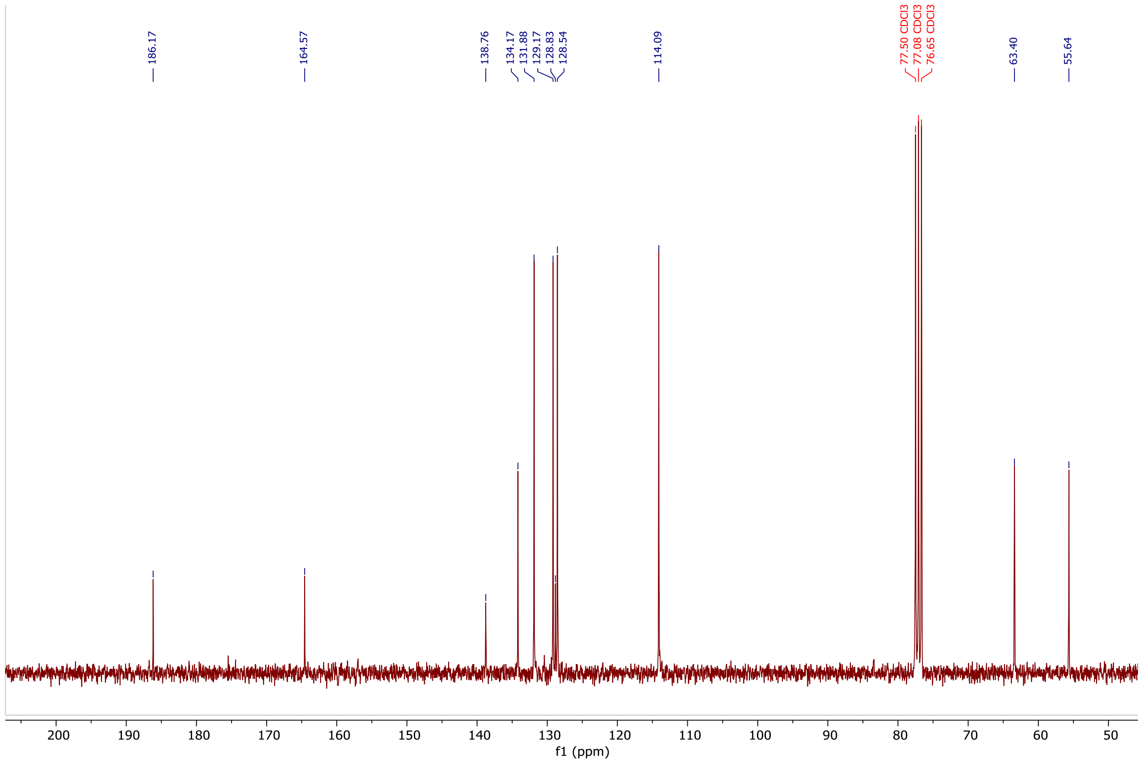
186.17
164.57
138.76
134.17
131.88
129.17
128.83
128.54
114.09
77.50 CDCl3
77.08 CDCl3
76.65 CDCl3
63.40
55.64
f1 (ppm)

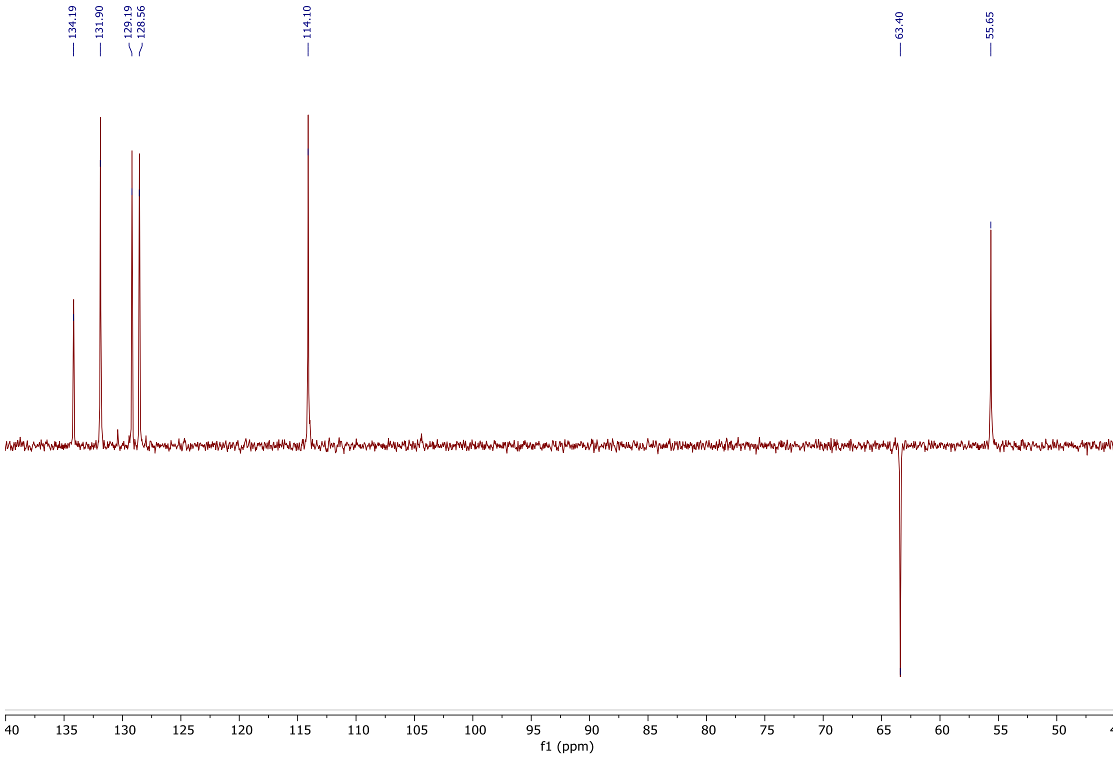
134.19
131.90
129.19
128.56
114.10
63.40
55.65
f1 (ppm)

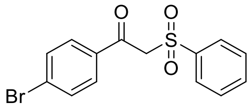

3d

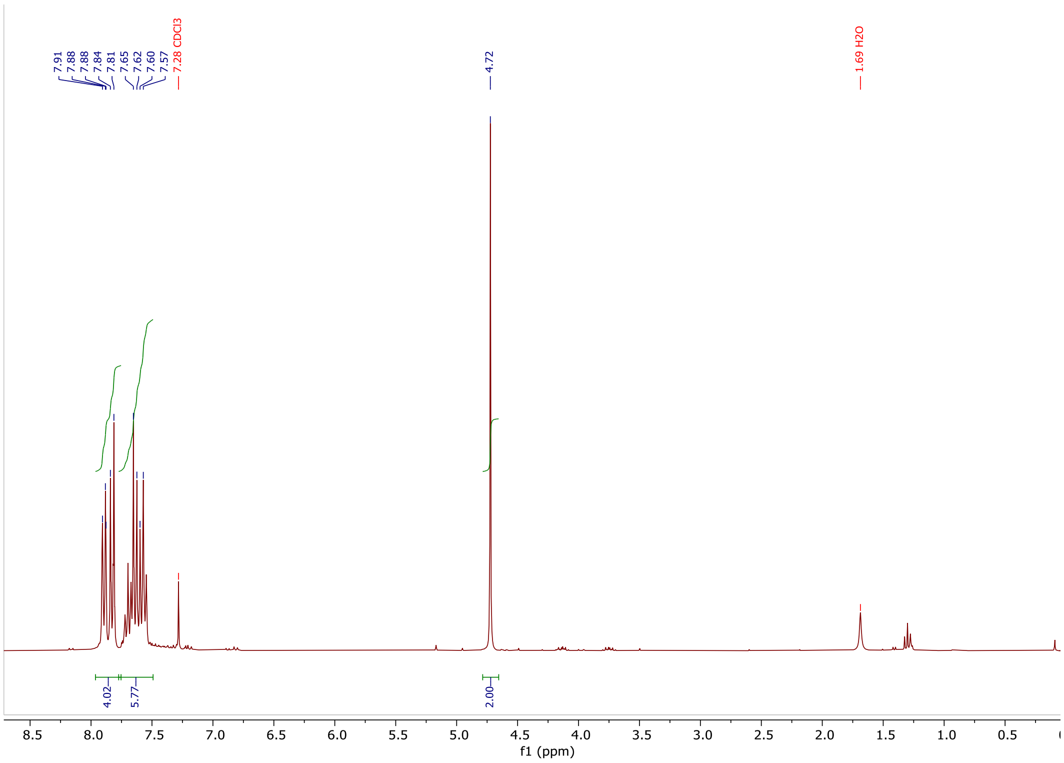
7.91
7.88
7.88
7.84
7.81
7.65
7.62
7.60
7.57
7.28 CDCl3
4.72
1.69 H2O
4.02
5.77
2.00
8.5 8.0 7.5 7.0 6.5 6.0 5.5 5.0 4.5 4.0 3.5 3.0 2.5 2.0 1.5 1.0 0.5
f1 (ppm)

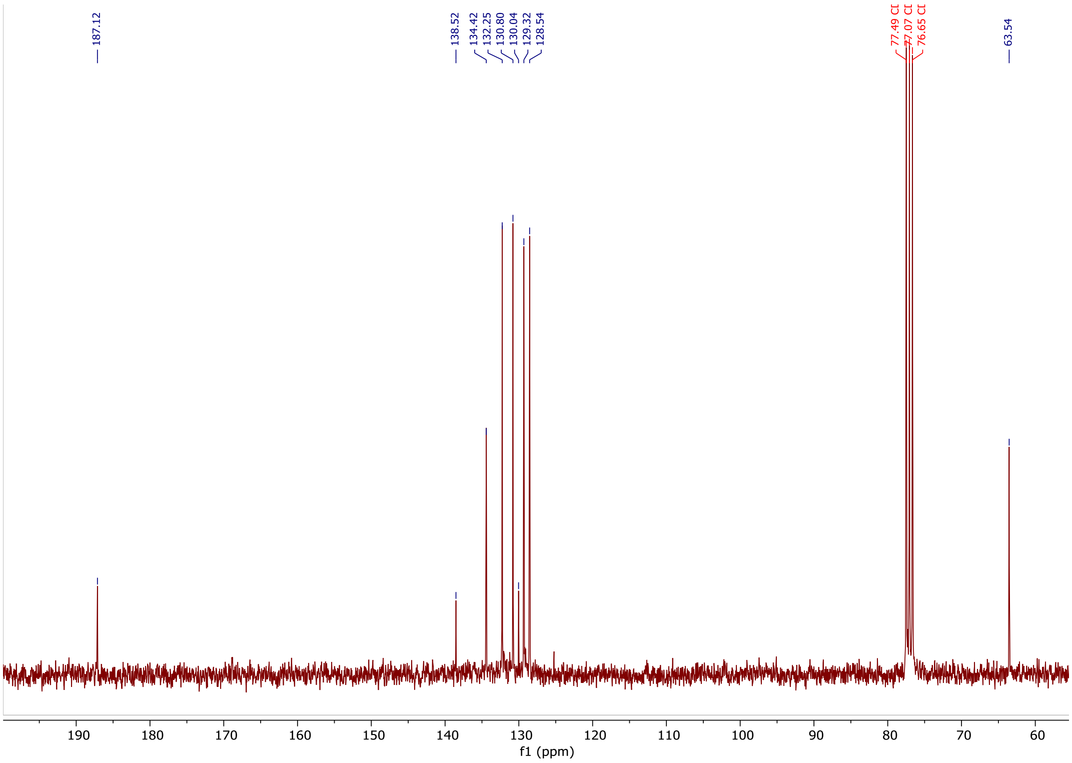
187.12
138.52
134.42
132.25
130.80
130.04
129.32
128.54
77.49 CDCl3
77.07 CDCl3
76.65 CDCl3
63.54
f1 (ppm)

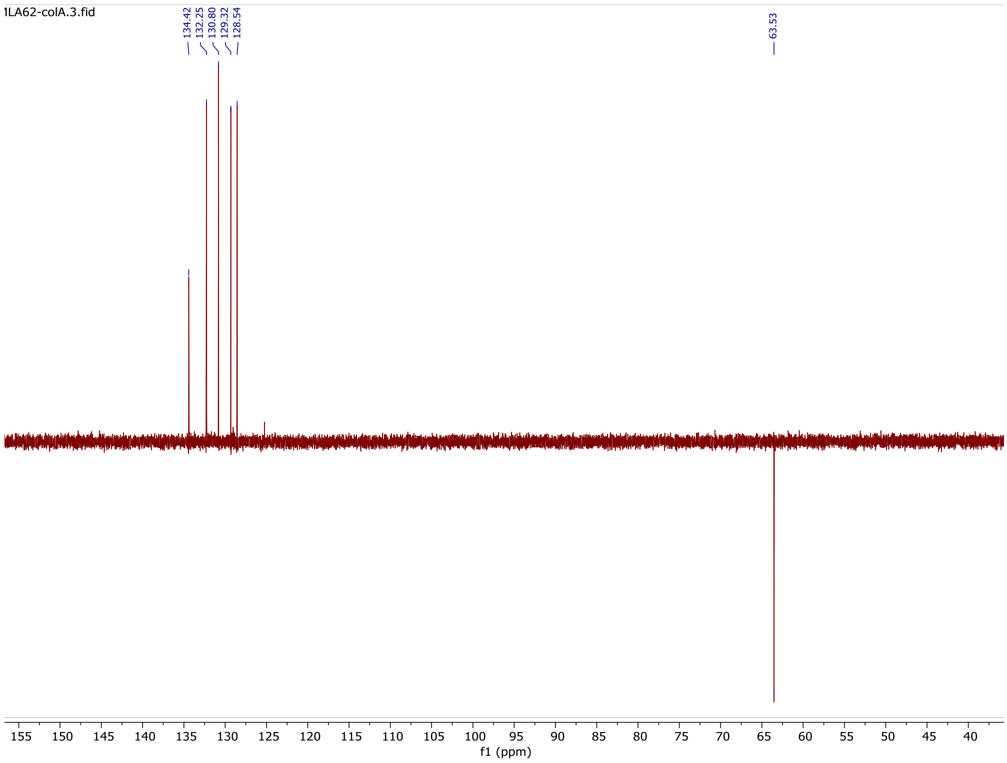
1LA62-colA.3.fid
134.42
132.25
130.80
129.32
128.54
63.53
f1 (ppm)

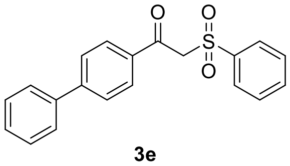

3e

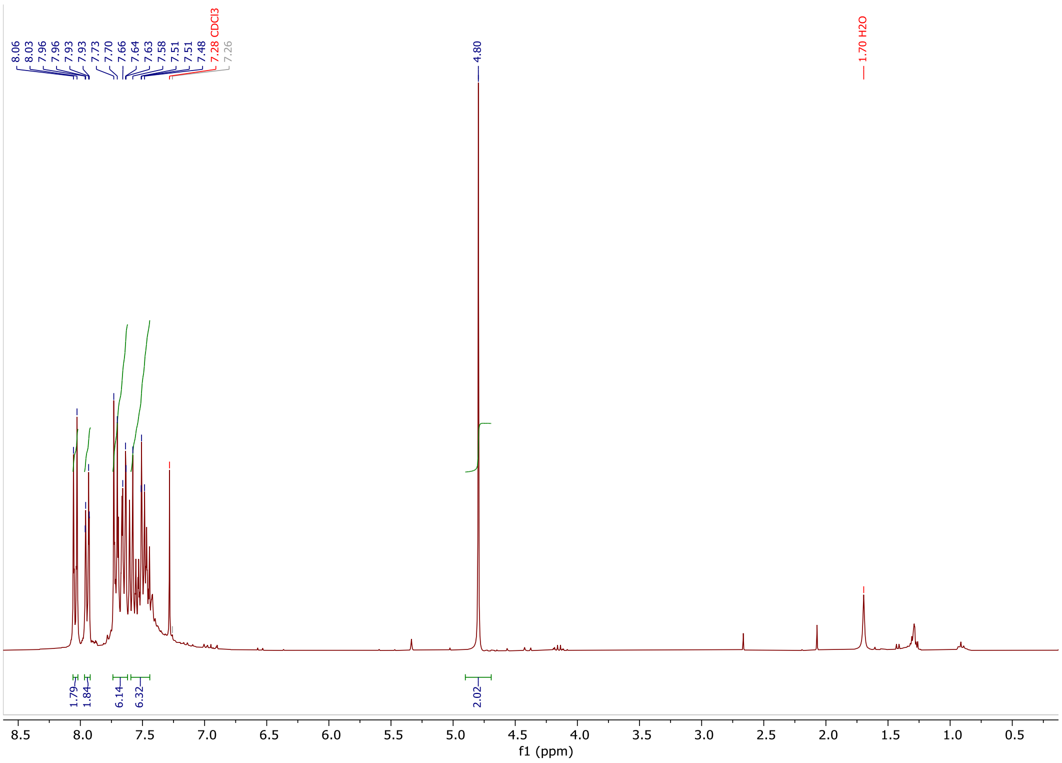

8.06
8.03
7.96
7.96
7.93
7.93
7.73
7.70
7.66
7.64
7.63
7.58
7.51
7.51
7.48
7.28 CDCl3
7.26
4.80
1.70 H2O
1.79
1.84
6.14
6.32
2.02
8.5
8.0
7.5
7.0
6.5
6.0
5.5
5.0
4.5
4.0
3.5
3.0
2.5
2.0
1.5
1.0
0.5
f1 (ppm)

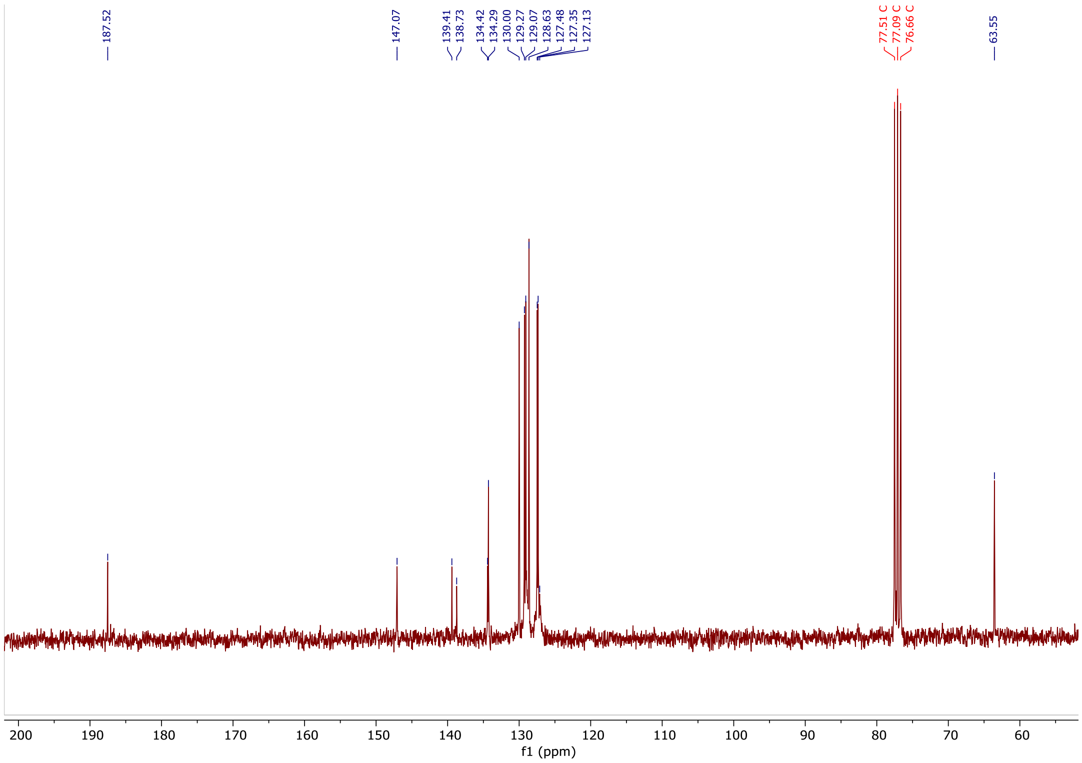
187.52
147.07
139.41
138.73
134.42
134.29
130.00
129.27
129.07
128.63
127.48
127.35
127.13
77.51 C
77.09 C
76.66 C
63.55
200 190 180 170 160 150 140 130 120 110 100 90 80 70 60
f1 (ppm)

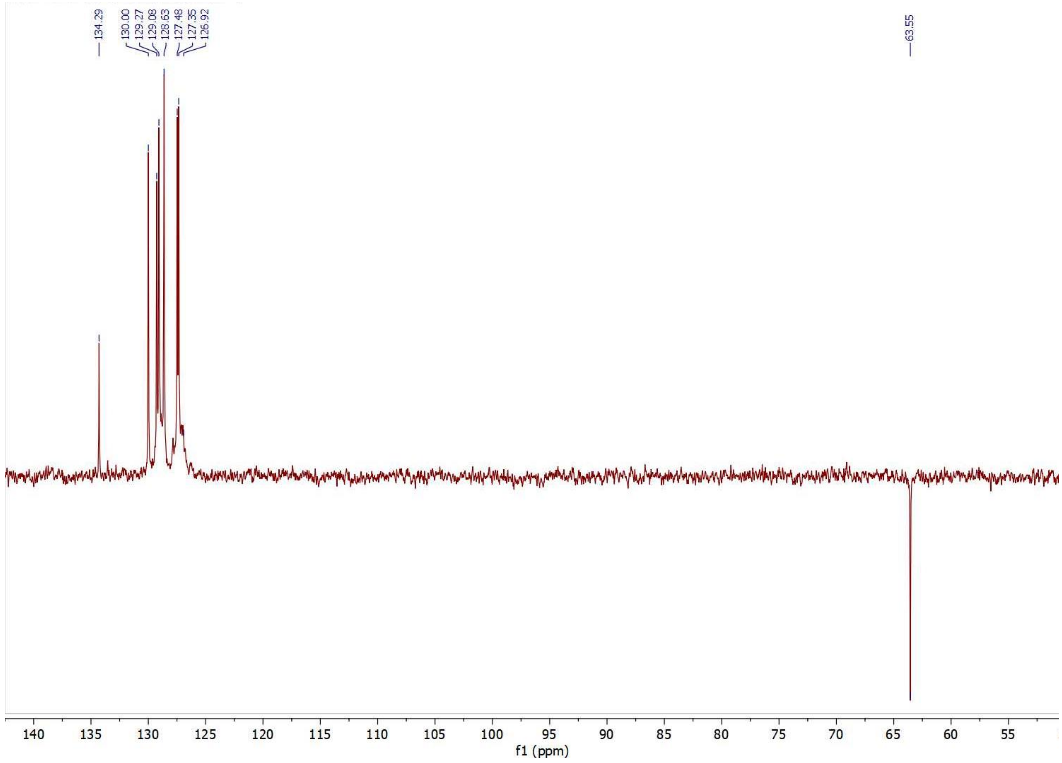
134.29
130.00
129.27
129.08
128.63
127.48
127.35
126.92
63.55
140 135 130 125 120 115 110 105 100 95 90 85 80 75 70 65 60 55
f1 (ppm)

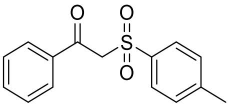

3f

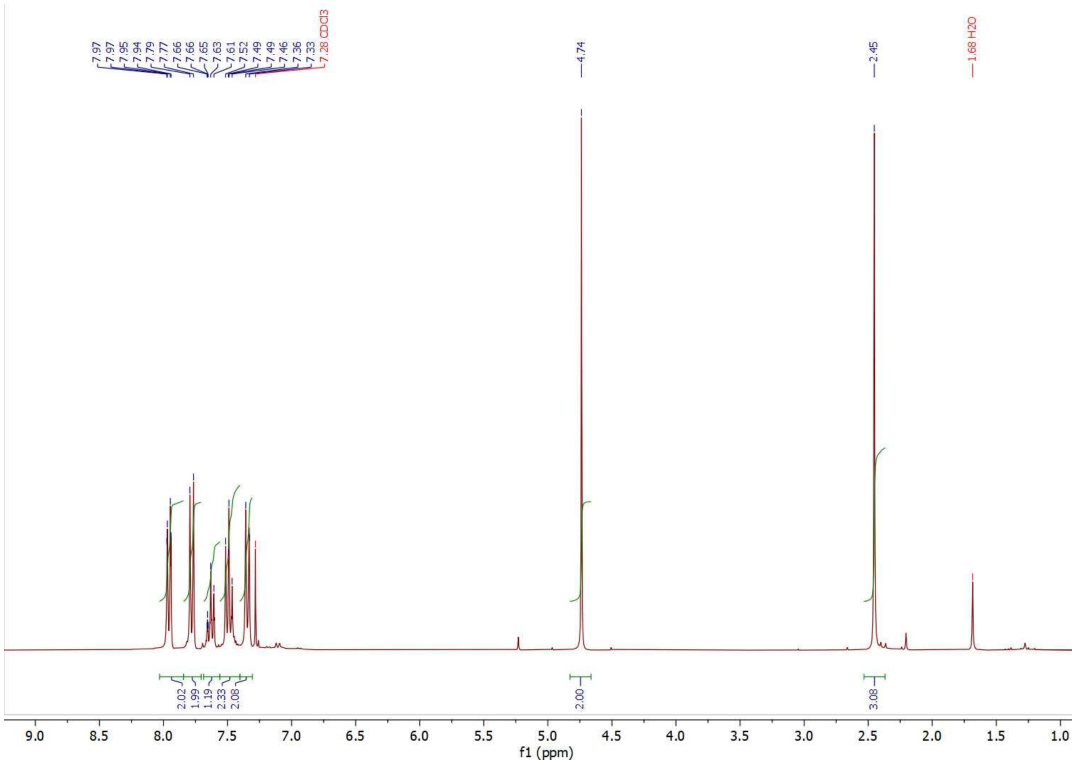

7.97
7.97
7.95
7.94
7.79
7.77
7.66
7.66
7.65
7.63
7.61
7.52
7.49
7.49
7.46
7.36
7.33
7.28 CDCl3
4.74
2.45
1.68 H2O
2.02
1.99
1.19
2.33
2.08
2.00
3.08
9.0
8.5
8.0
7.5
7.0
6.5
6.0
5.5
5.0
4.5
4.0
3.5
3.0
2.5
2.0
1.5
1.0
f1 (ppm)

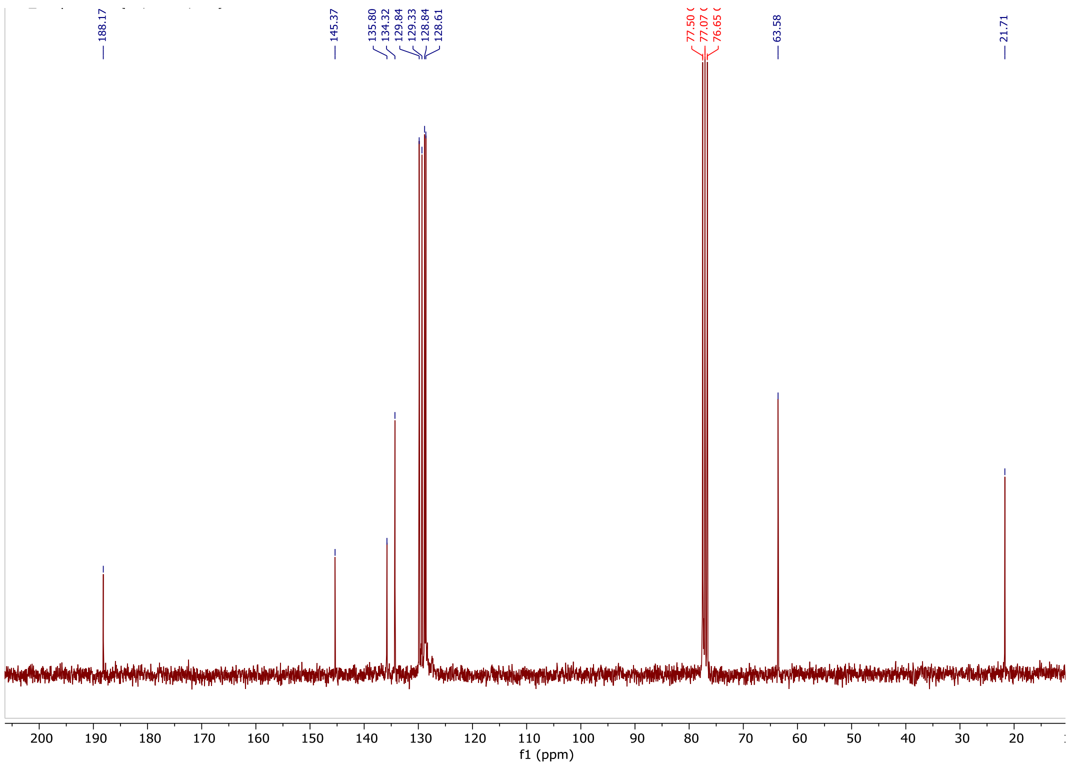

188.17
145.37
135.80
134.32
129.84
129.33
128.84
128.61
77.50
77.07
76.65
63.58
21.71
200
190
180
170
160
150
140
130
120
110
100
90
80
70
60
50
40
30
20
f1 (ppm)

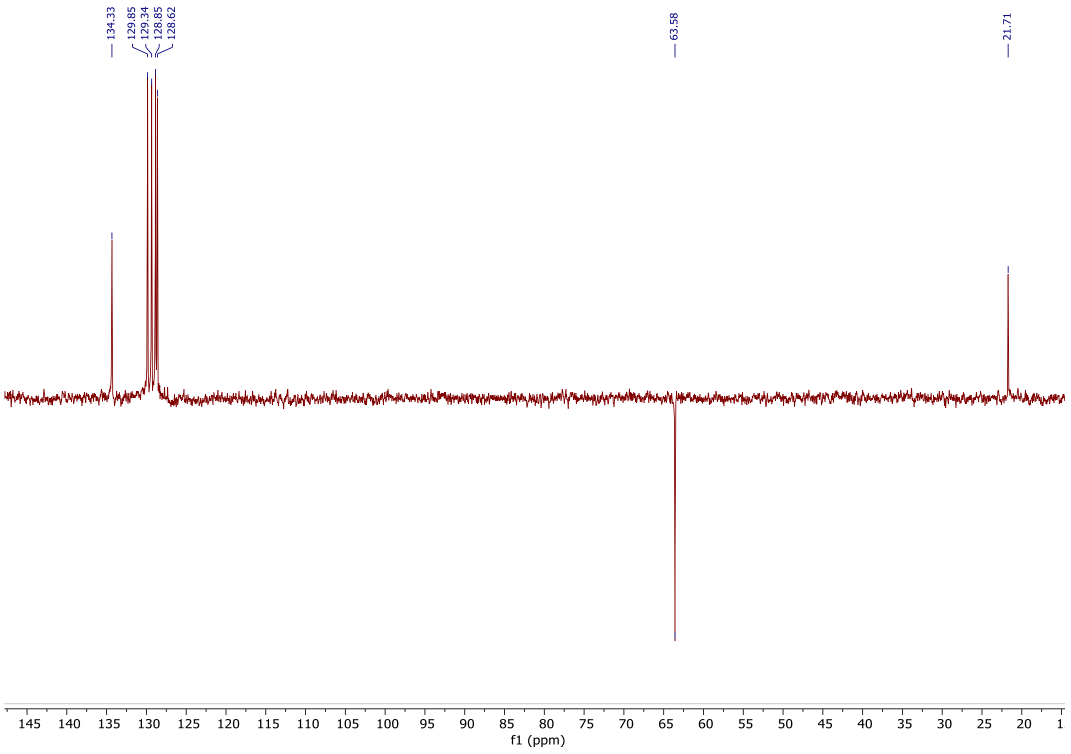

134.33
129.85
129.34
128.85
128.62
63.58
21.71
145
140
135
130
125
120
115
110
105
100
95
90
85
80
75
70
65
60
55
50
45
40
35
30
25
20
f1 (ppm)

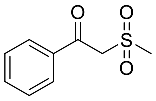

3g

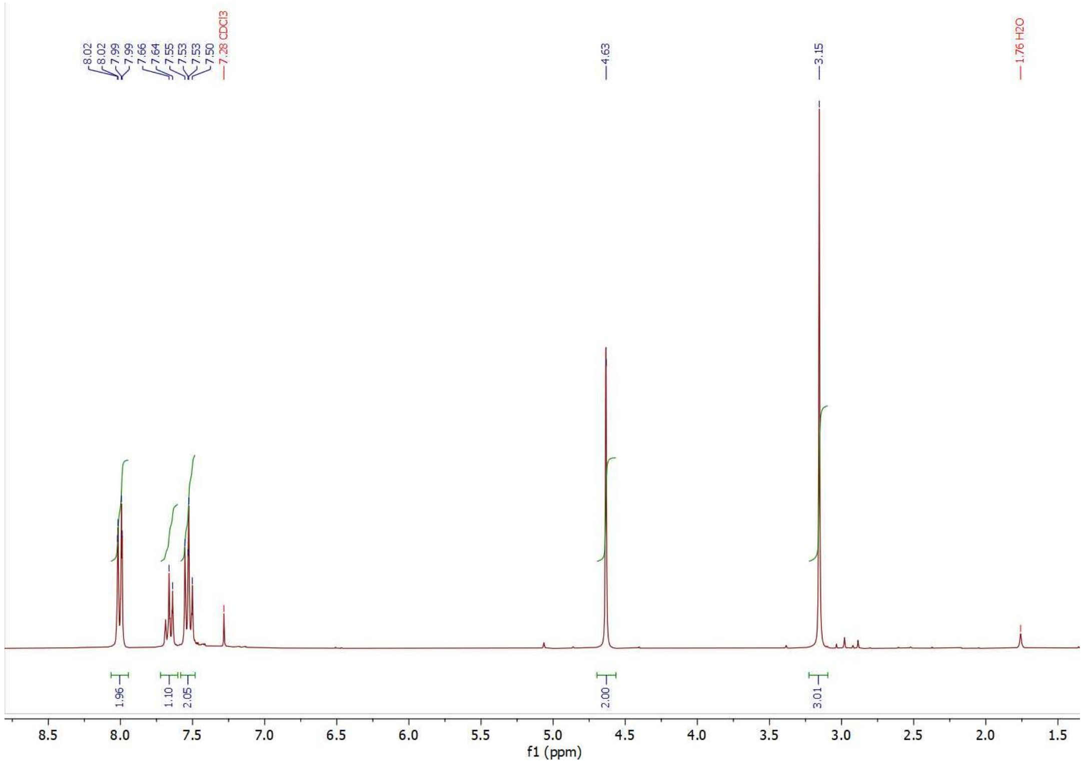
8.02
8.02
7.99
7.99
7.66
7.64
7.55
7.53
7.53
7.50
7.28 CDCl3
4.63
3.15
1.76 H2O
1.96
1.10
2.05
2.00
3.01
8.5
8.0
7.5
7.0
6.5
6.0
5.5
5.0
4.5
4.0
3.5
3.0
2.5
2.0
1.5
f1 (ppm)

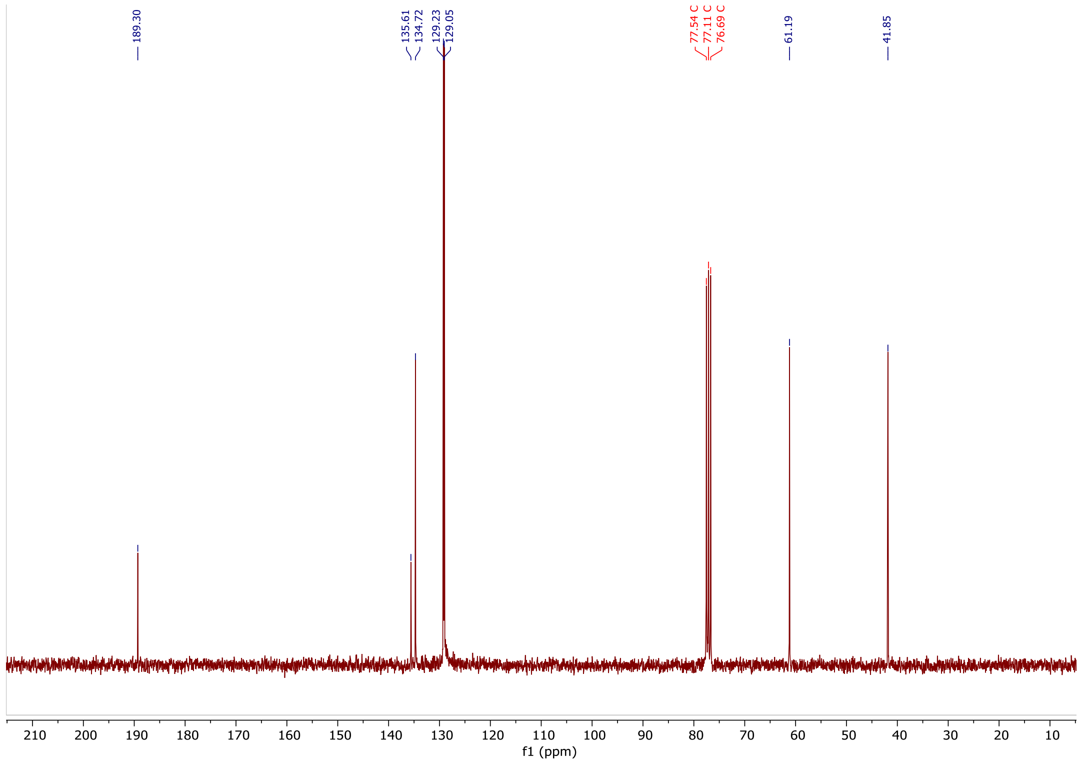

189.30
135.61
134.72
129.23
129.05
77.54 C
77.11 C
76.69 C
61.19
41.85
210 200 190 180 170 160 150 140 130 120 110 100 90 80 70 60 50 40 30 20 10
f1 (ppm)

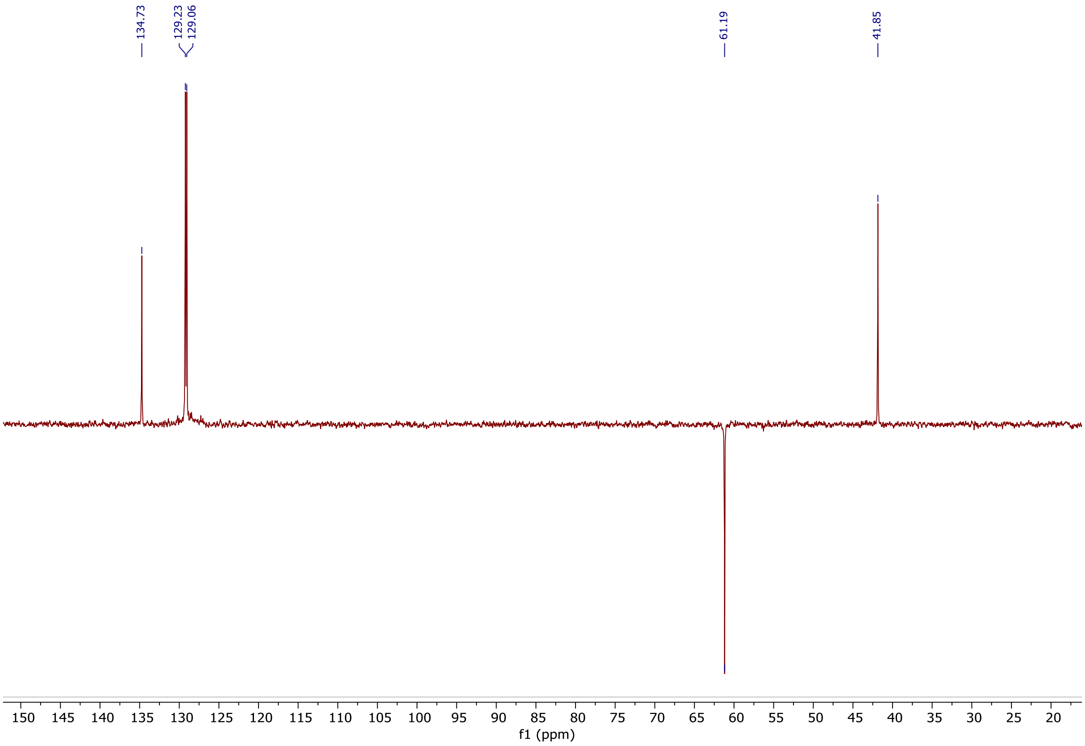

134.73
129.23
129.06
61.19
41.85
150 145 140 135 130 125 120 115 110 105 100 95 90 85 80 75 70 65 60 55 50 45 40 35 30 25 20
f1 (ppm)

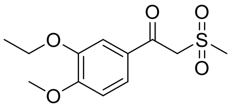

3h

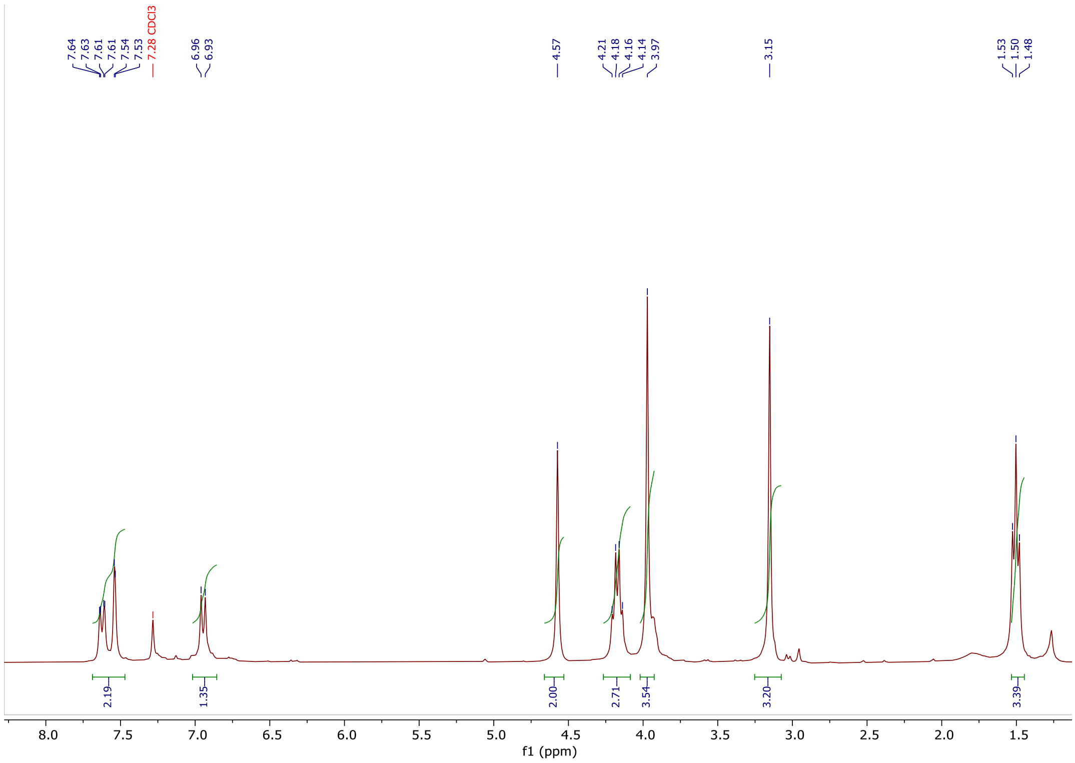
7.64
7.63
7.61
7.61
7.54
7.53
7.28 CDCl3
6.96
6.93
4.57
4.21
4.18
4.16
4.14
3.97
3.15
1.53
1.50
1.48
2.19
1.35
2.00
2.71
3.54
3.20
3.39
8.0
7.5
7.0
6.5
6.0
5.5
5.0
4.5
4.0
3.5
3.0
2.5
2.0
1.5
f1 (ppm)

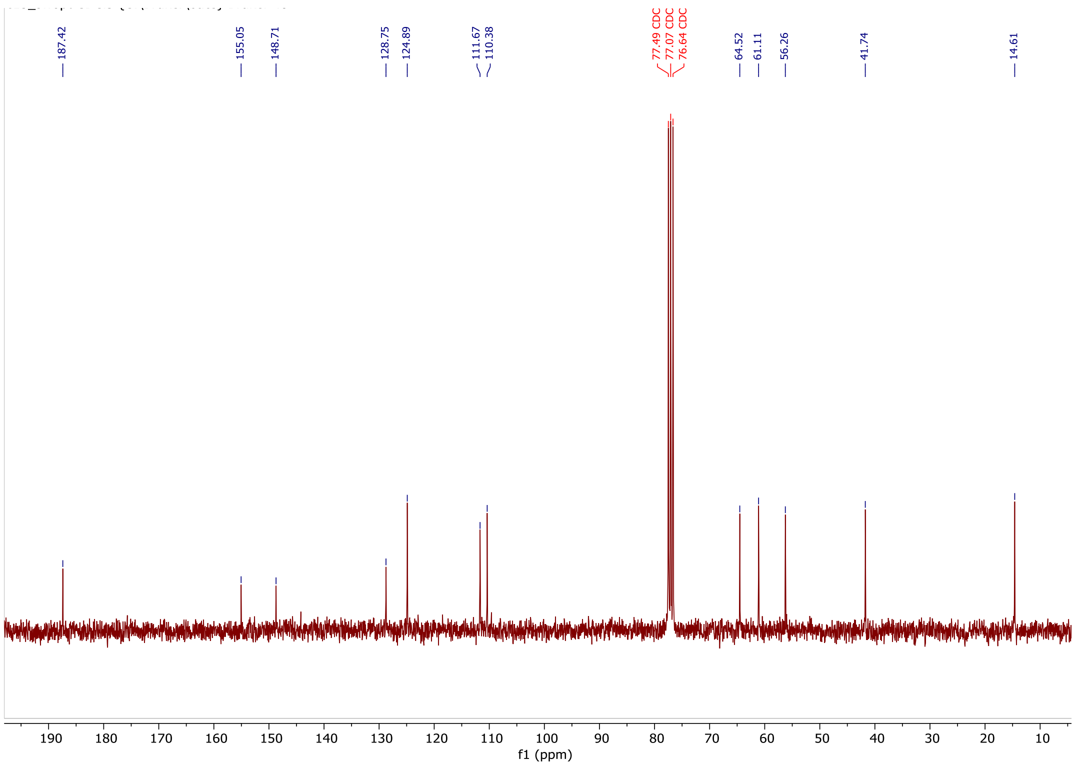
187.42
155.05
148.71
128.75
124.89
111.67
110.38
77.49 CDC
77.07 CDC
76.64 CDC
64.52
61.11
56.26
41.74
14.61
190 180 170 160 150 140 130 120 110 100 90 80 70 60 50 40 30 20 10
f1 (ppm)

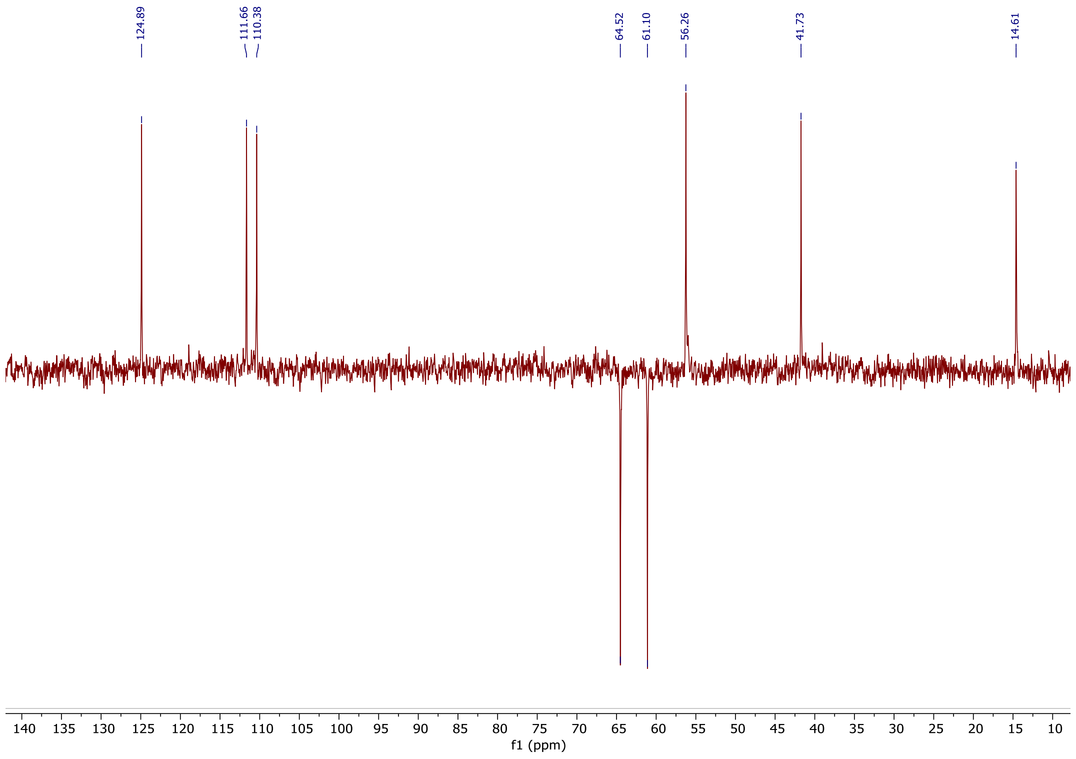
124.89
111.66
110.38
64.52
61.10
56.26
41.73
14.61
140 135 130 125 120 115 110 105 100 95 90 85 80 75 70 65 60 55 50 45 40 35 30 25 20 15 10
f1 (ppm)

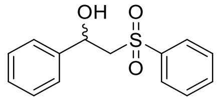
OH
O
S
O

4a

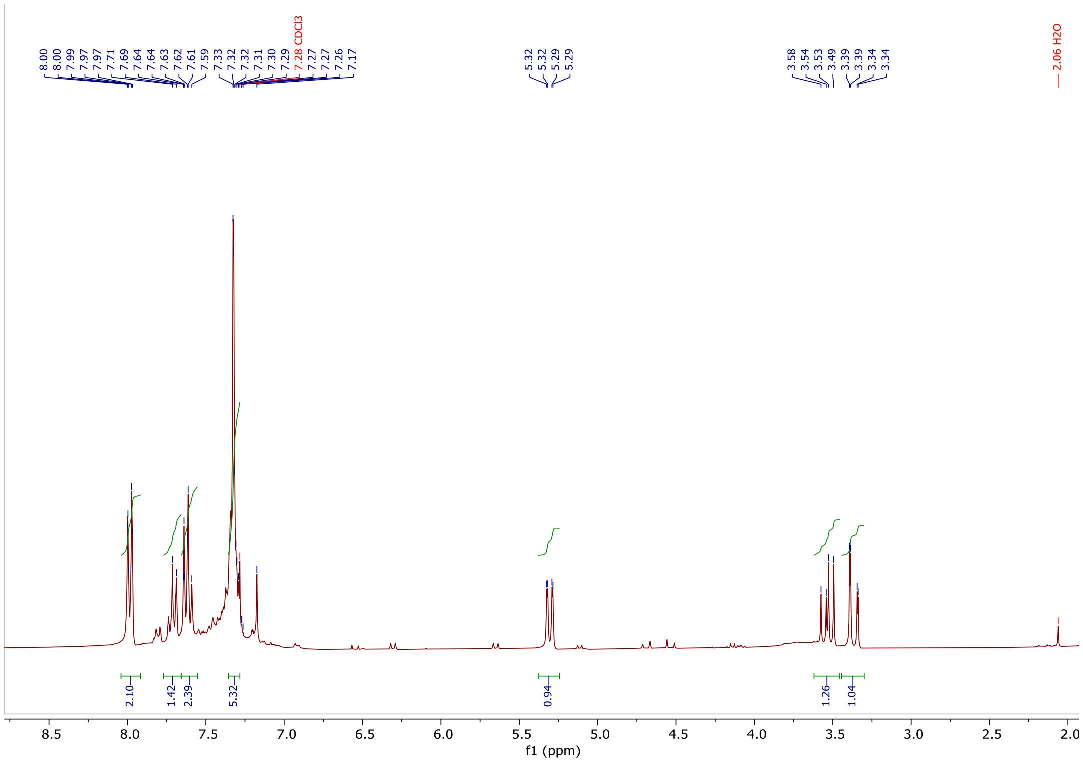
8.00
8.00
7.99
7.97
7.97
7.71
7.69
7.64
7.64
7.63
7.62
7.61
7.59
7.33
7.32
7.32
7.31
7.30
7.29
7.28 CDCl3
7.27
7.27
7.26
7.17
5.32
5.32
5.29
5.29
3.58
3.54
3.53
3.49
3.39
3.39
3.34
3.34
2.06 H2O
2.10
1.42
2.39
5.32
0.94
1.26
1.04
8.5
8.0
7.5
7.0
6.5
6.0
5.5
5.0
4.5
4.0
3.5
3.0
2.5
2.0
f1 (ppm)

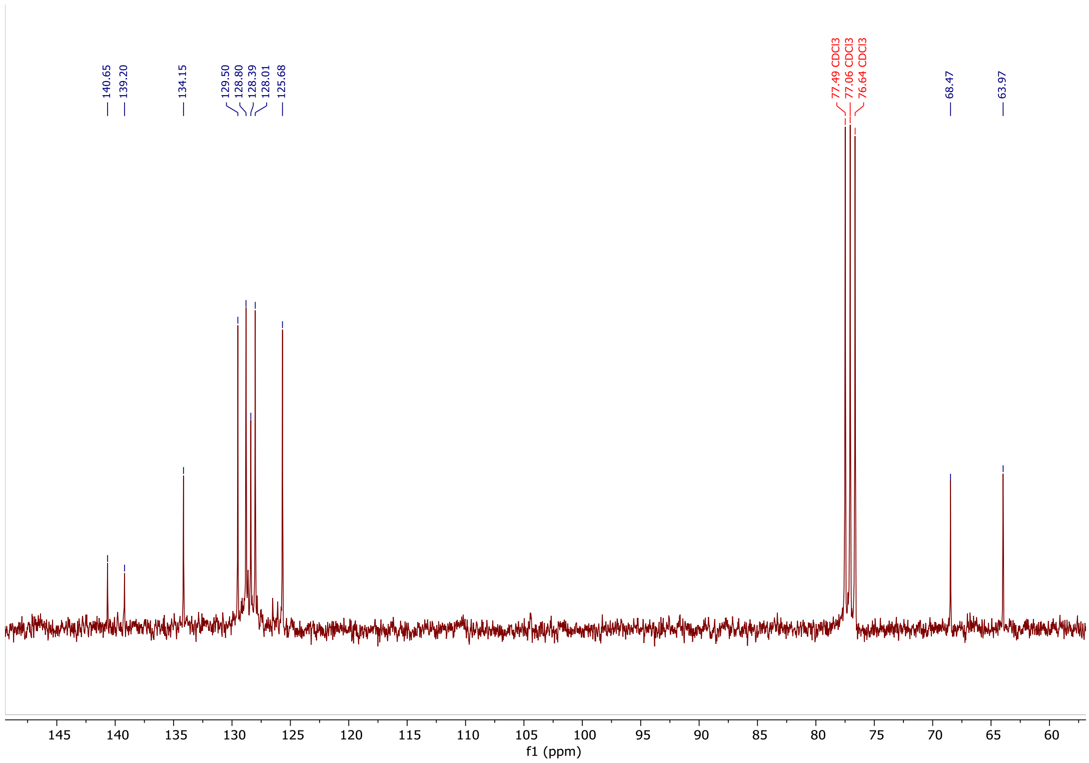
140.65
139.20
134.15
129.50
128.80
128.39
128.01
125.68
77.49 CDCl3
77.06 CDCl3
76.64 CDCl3
68.47
63.97
145 140 135 130 125 120 115 110 105 100 95 90 85 80 75 70 65 60
f1 (ppm)

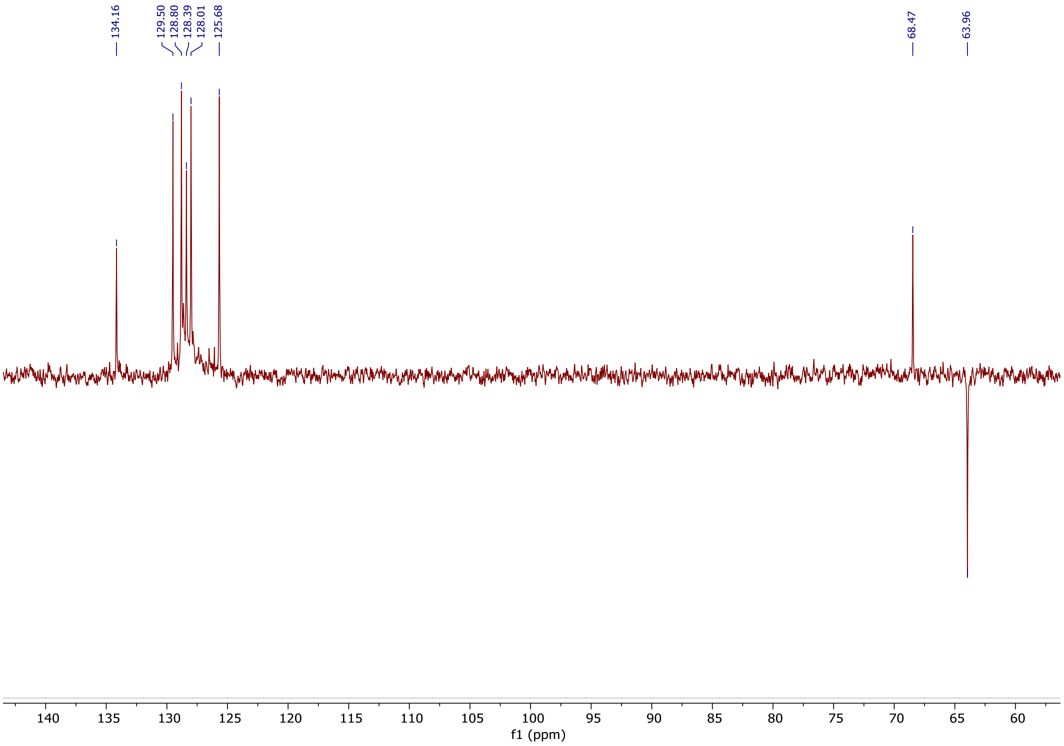
134.16
129.50
128.80
128.39
128.01
125.68
68.47
63.96
140 135 130 125 120 115 110 105 100 95 90 85 80 75 70 65 60
f1 (ppm)

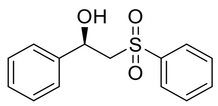

(*R*)-**4a**

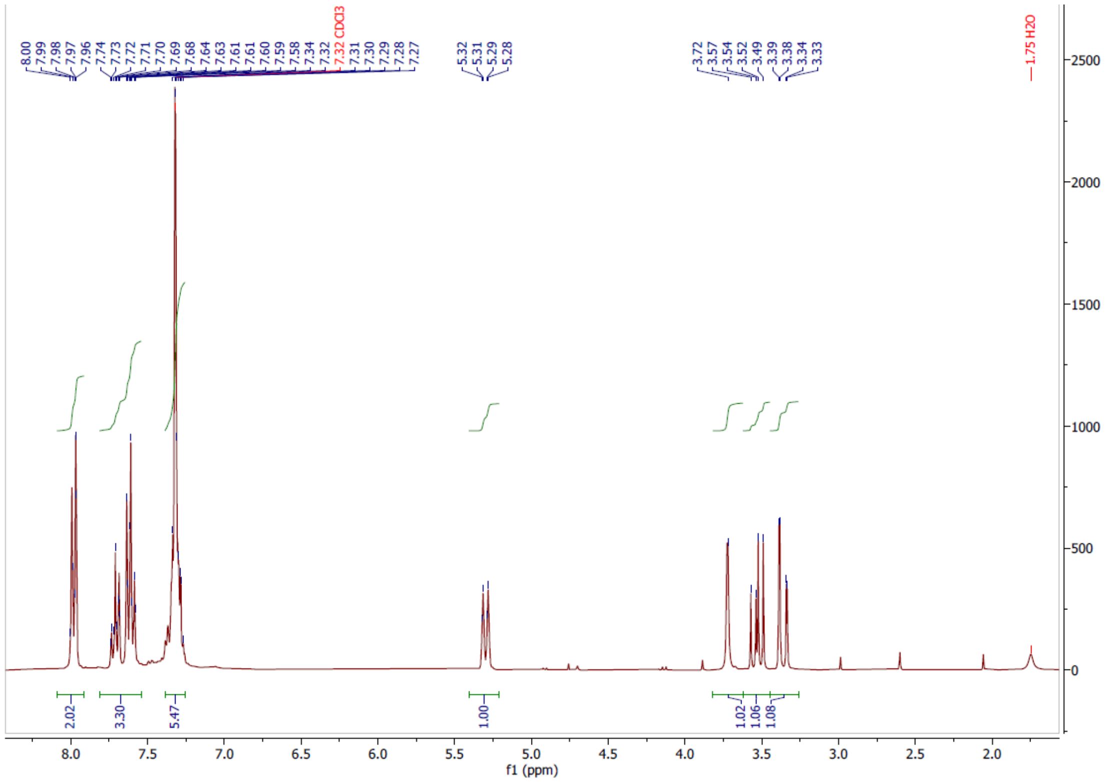

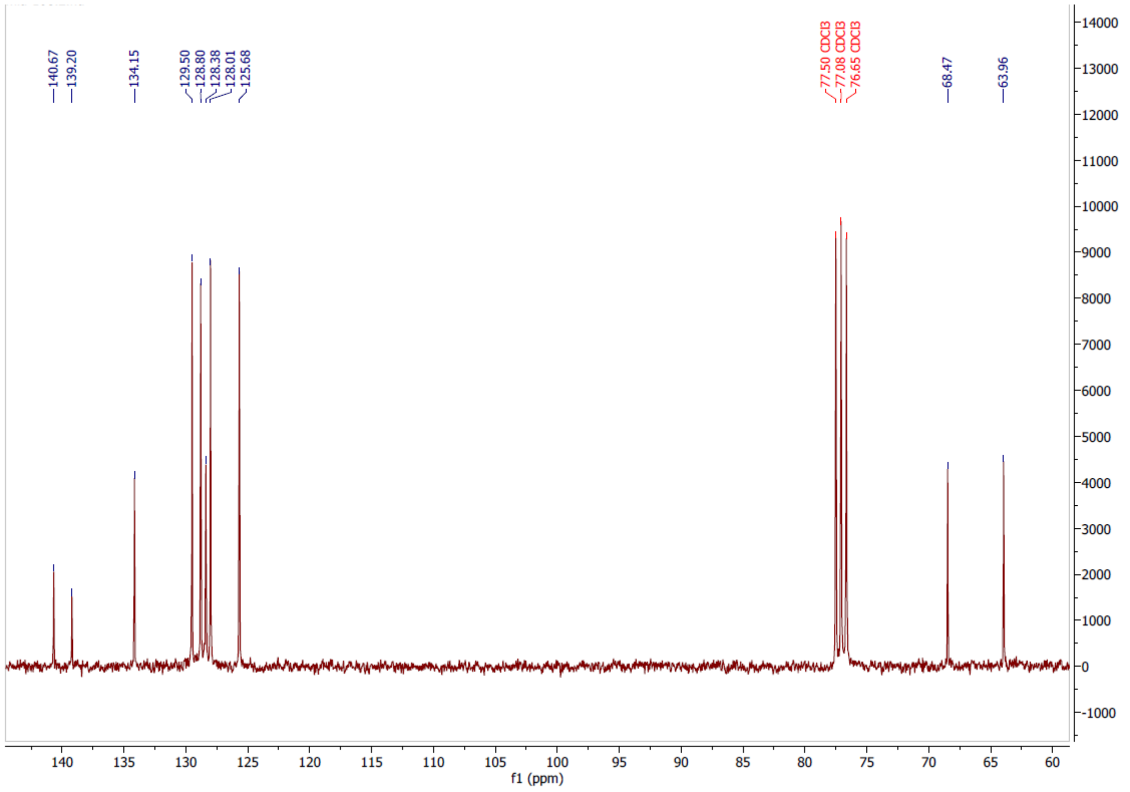

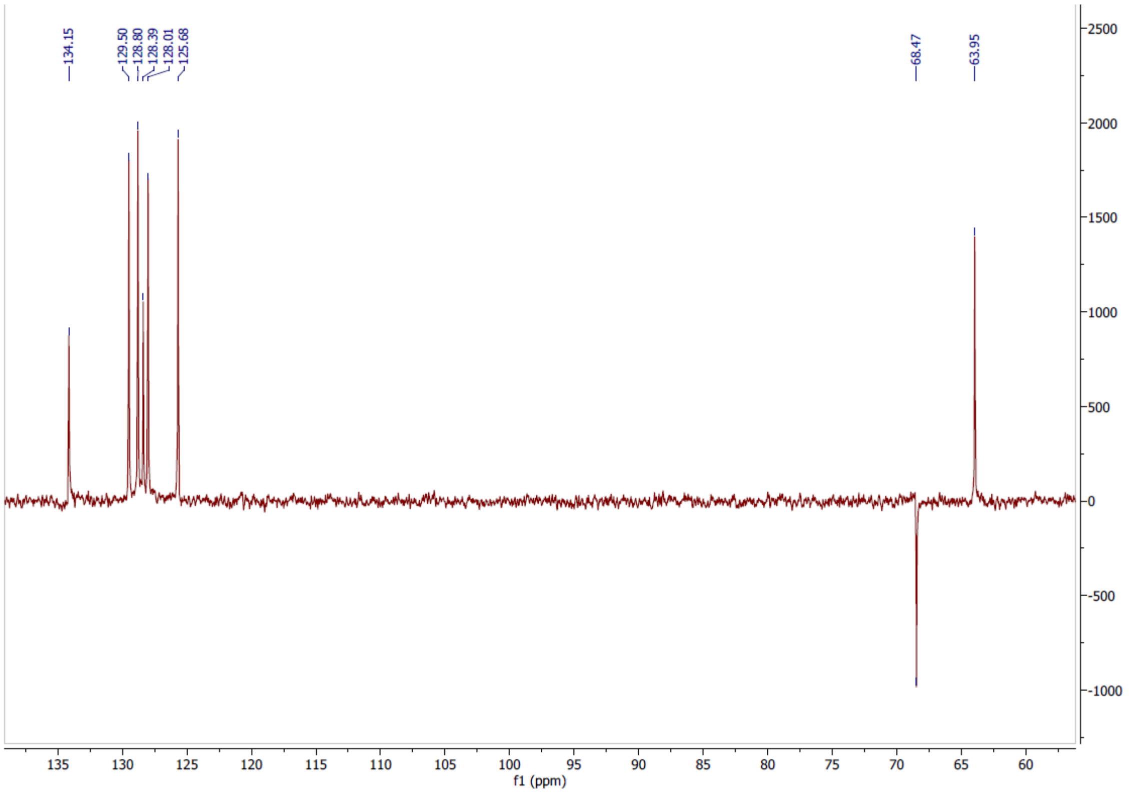
134.15
129.50
128.80
128.39
128.01
125.68
68.47
63.95
2500
2000
1500
1000
500
0
-500
-1000
135
130
125
120
115
110
105
100
95
90
85
80
75
70
65
60
f1 (ppm)

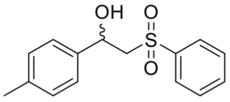

4b

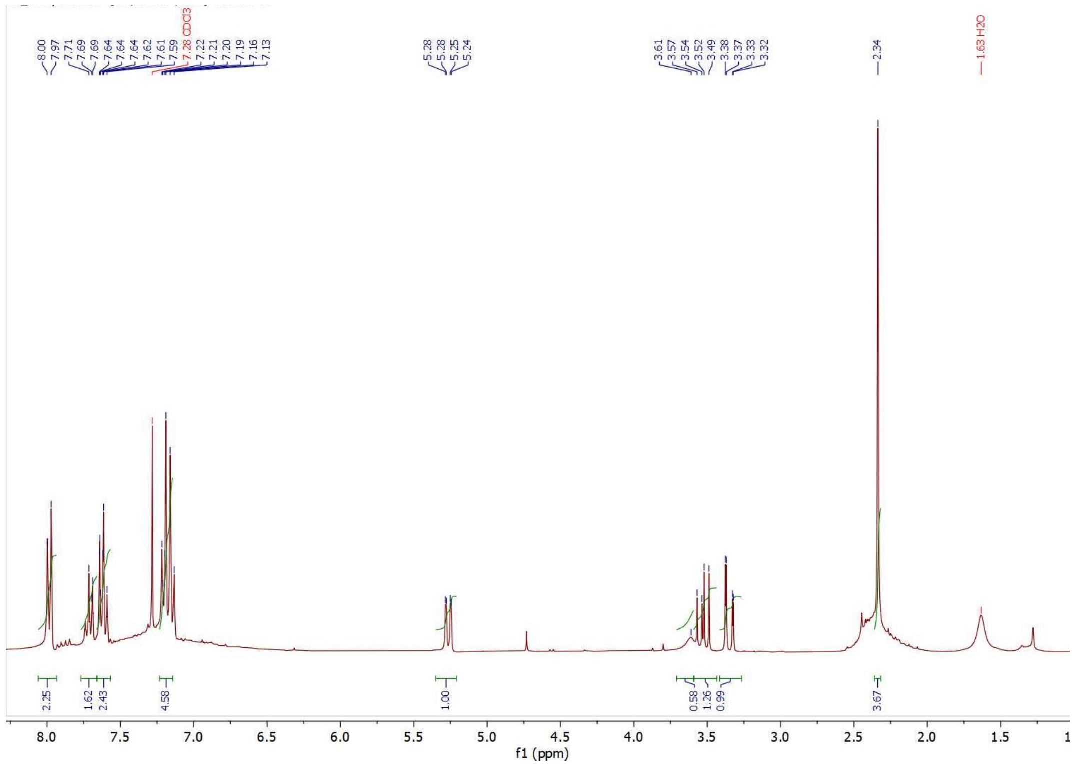

8.00
7.97
7.71
7.69
7.69
7.64
7.64
7.64
7.62
7.61
7.59
7.28 CDCl3
7.22
7.21
7.20
7.19
7.16
7.13
5.28
5.28
5.25
5.24
3.61
3.57
3.54
3.52
3.49
3.38
3.37
3.33
3.32
2.34
1.63 H2O
2.25
1.62
2.43
4.58
1.00
0.58
1.26
0.99
3.67
f1 (ppm)

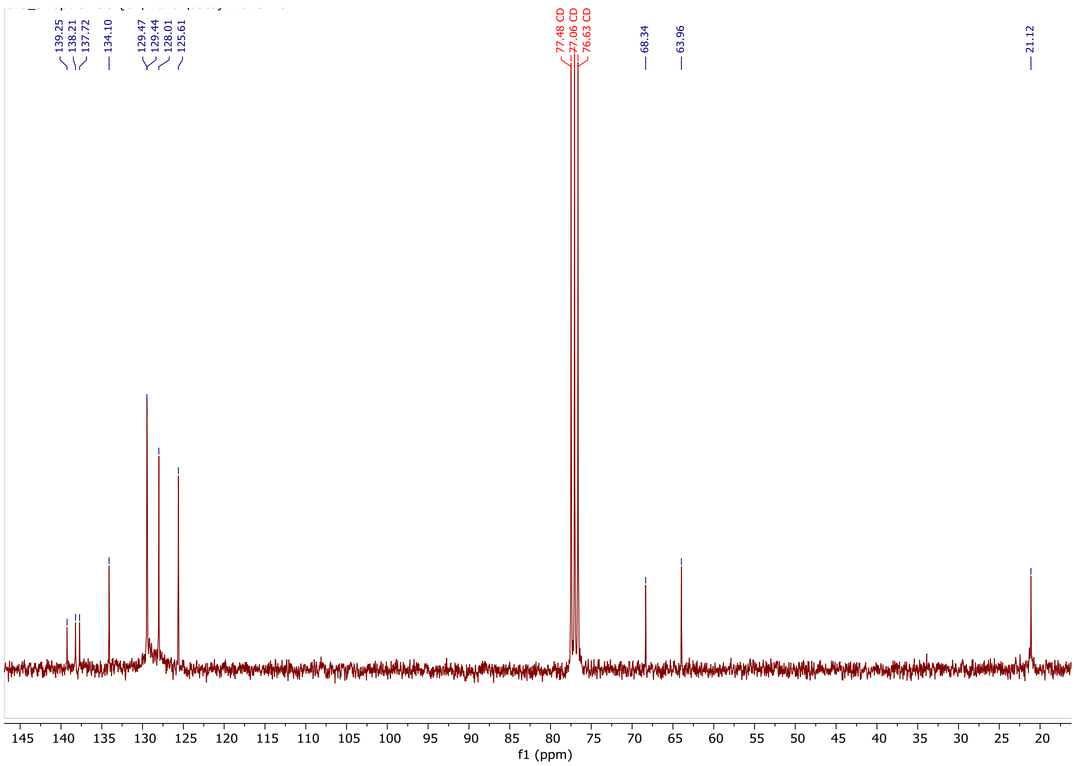

139.25
138.21
137.72
134.10
129.47
129.44
128.01
125.61
77.48 CD
77.06 CD
76.63 CD
68.34
63.96
21.12
145 140 135 130 125 120 115 110 105 100 95 90 85 80 75 70 65 60 55 50 45 40 35 30 25 20
f1 (ppm)

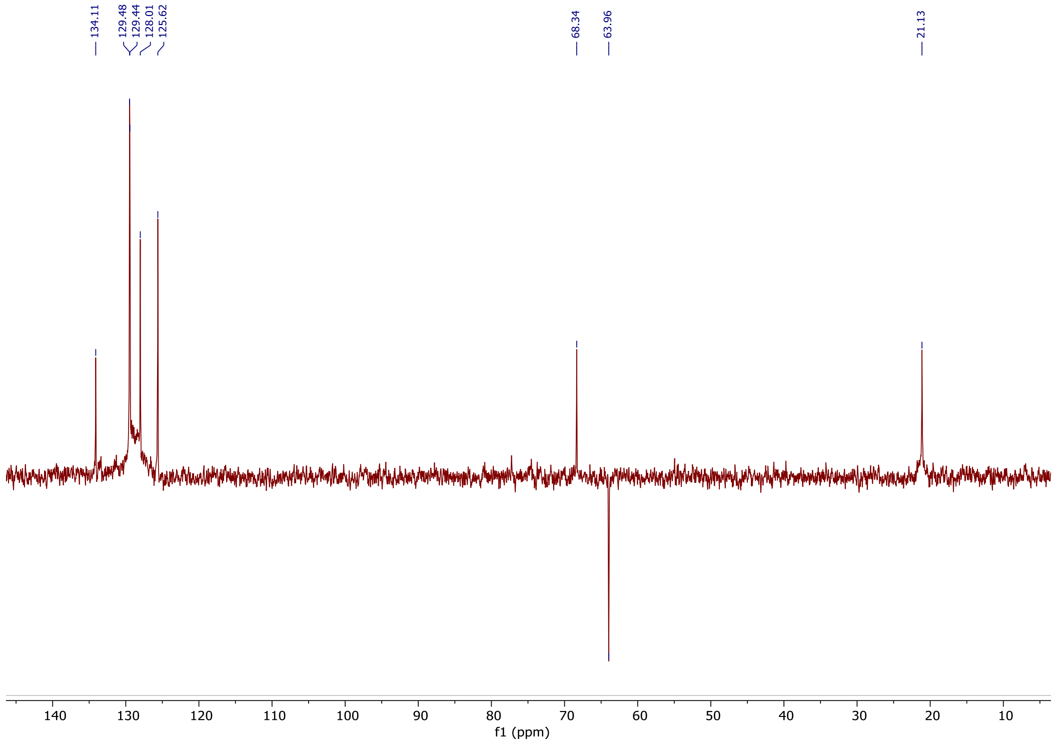

134.11
129.48
129.44
128.01
125.62
68.34
63.96
21.13
140 130 120 110 100 90 80 70 60 50 40 30 20 10
f1 (ppm)

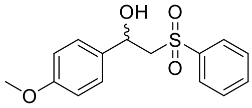
OH
O
S
O
O

4c

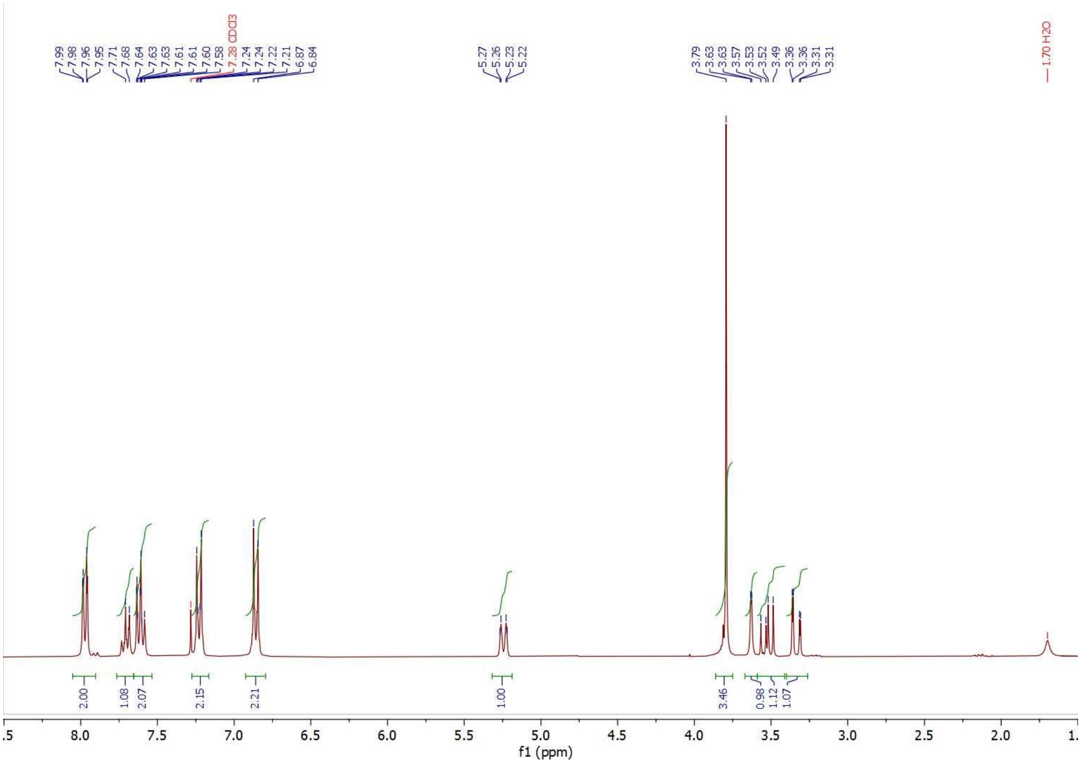
7.99
7.98
7.96
7.95
7.71
7.68
7.64
7.63
7.63
7.61
7.60
7.58
7.28 CDCl3
7.24
7.24
7.22
7.21
6.87
6.84
5.27
5.26
5.23
5.22
3.79
3.63
3.63
3.57
3.53
3.52
3.49
3.36
3.36
3.31
3.31
1.70 H2O
2.00
1.08
2.07
2.15
2.21
1.00
3.46
0.98
1.12
1.07
8.0
7.5
7.0
6.5
6.0
5.5
5.0
4.5
4.0
3.5
3.0
2.5
2.0
f1 (ppm)

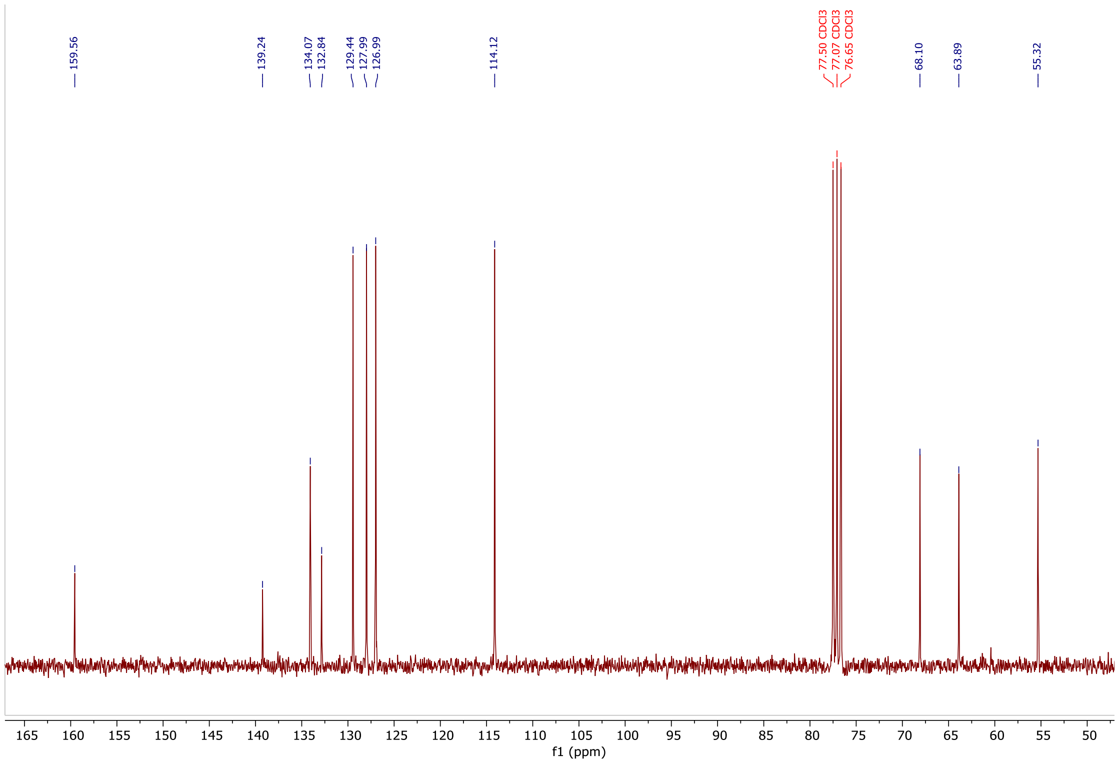
159.56
139.24
134.07
132.84
129.44
127.99
126.99
114.12
77.50 CDCl3
77.07 CDCl3
76.65 CDCl3
68.10
63.89
55.32
165 160 155 150 145 140 135 130 125 120 115 110 105 100 95 90 85 80 75 70 65 60 55 50
f1 (ppm)

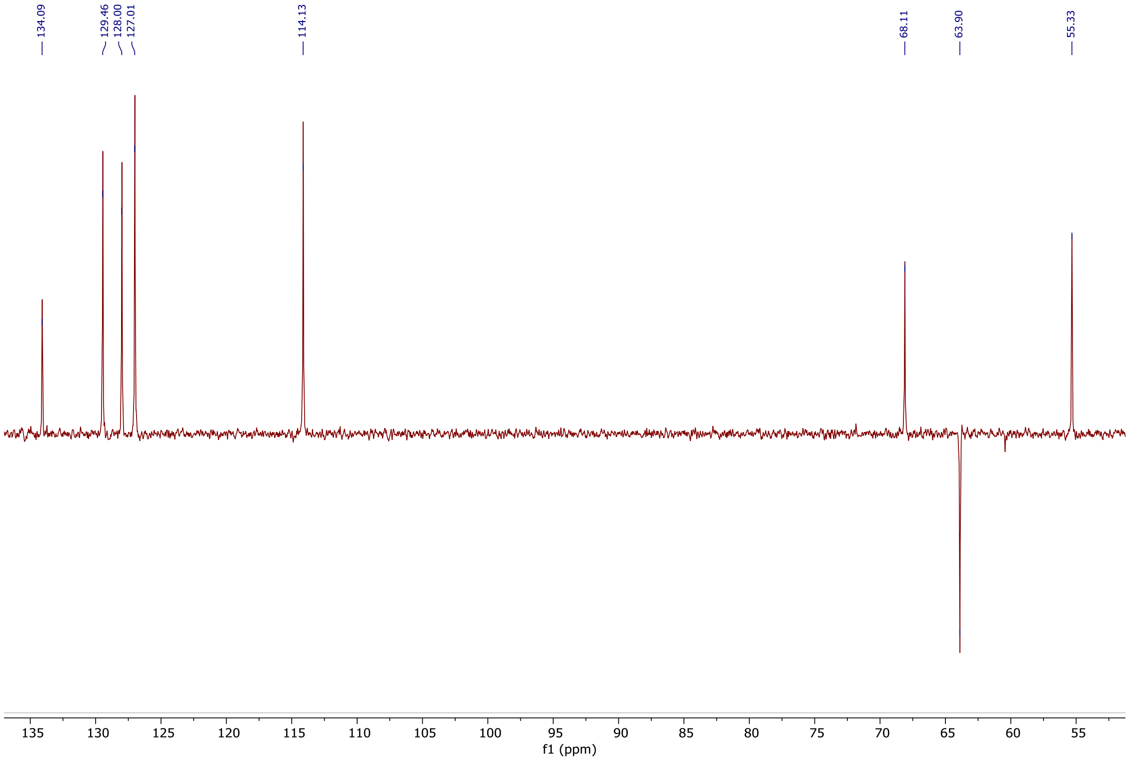
134.09
129.46
128.00
127.01
114.13
68.11
63.90
55.33
135 130 125 120 115 110 105 100 95 90 85 80 75 70 65 60 55
f1 (ppm)

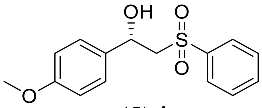

(*S*)-**4c**

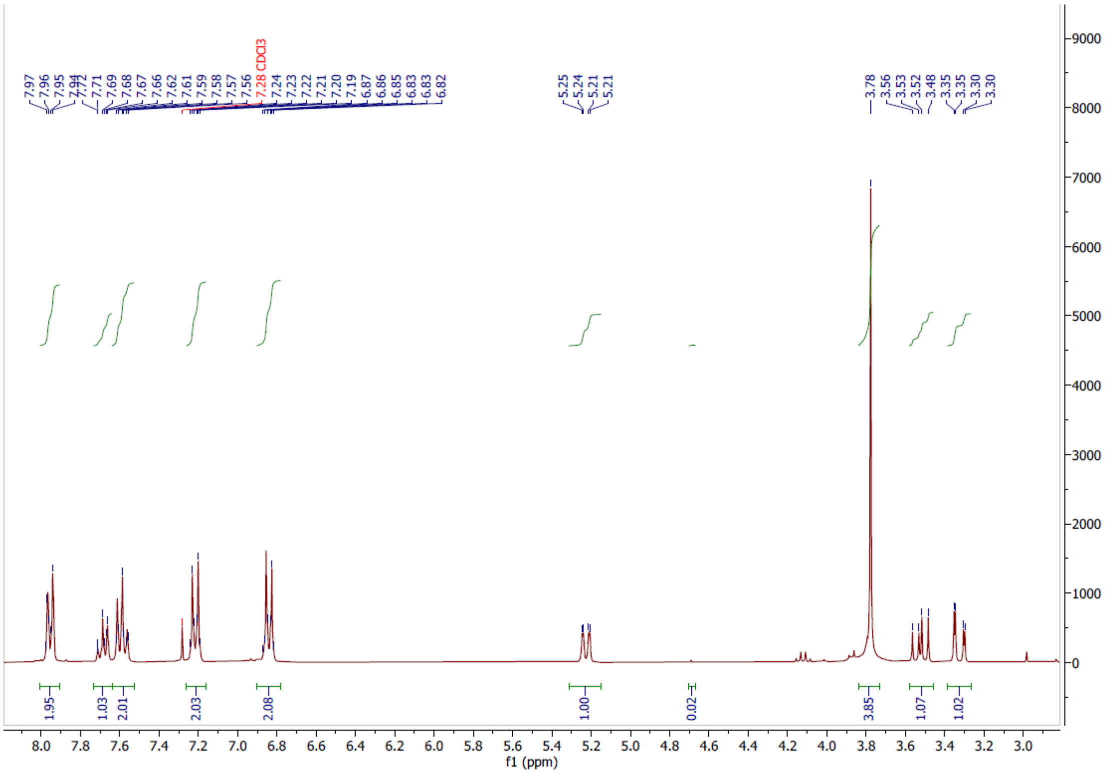

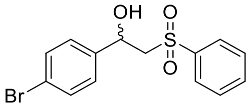

OH
O
S
O
Br

4d

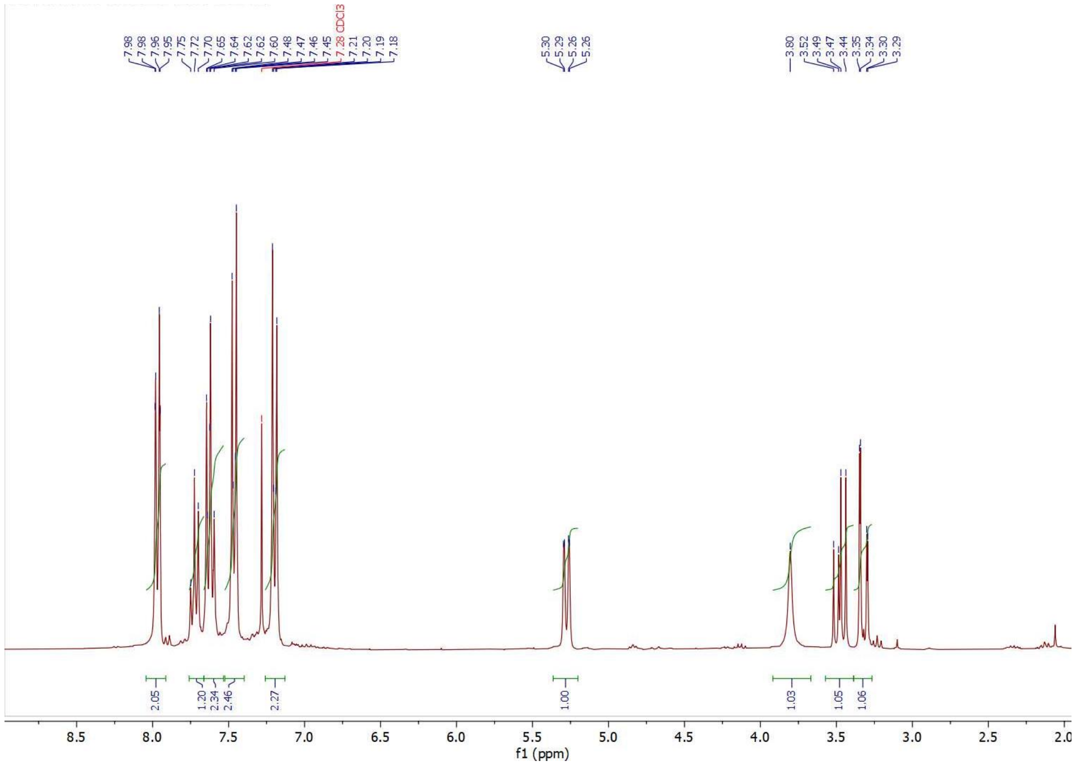

7.98
7.98
7.96
7.95
7.75
7.72
7.70
7.65
7.64
7.62
7.62
7.60
7.48
7.47
7.46
7.45
7.28 CDCl3
7.21
7.20
7.19
7.18
5.30
5.29
5.26
5.26
3.80
3.52
3.49
3.47
3.44
3.35
3.34
3.30
3.29
2.05
1.20
2.34
2.46
2.27
1.00
1.03
1.05
1.06
8.5
8.0
7.5
7.0
6.5
6.0
5.5
5.0
4.5
4.0
3.5
3.0
2.5
2.0
f1 (ppm)

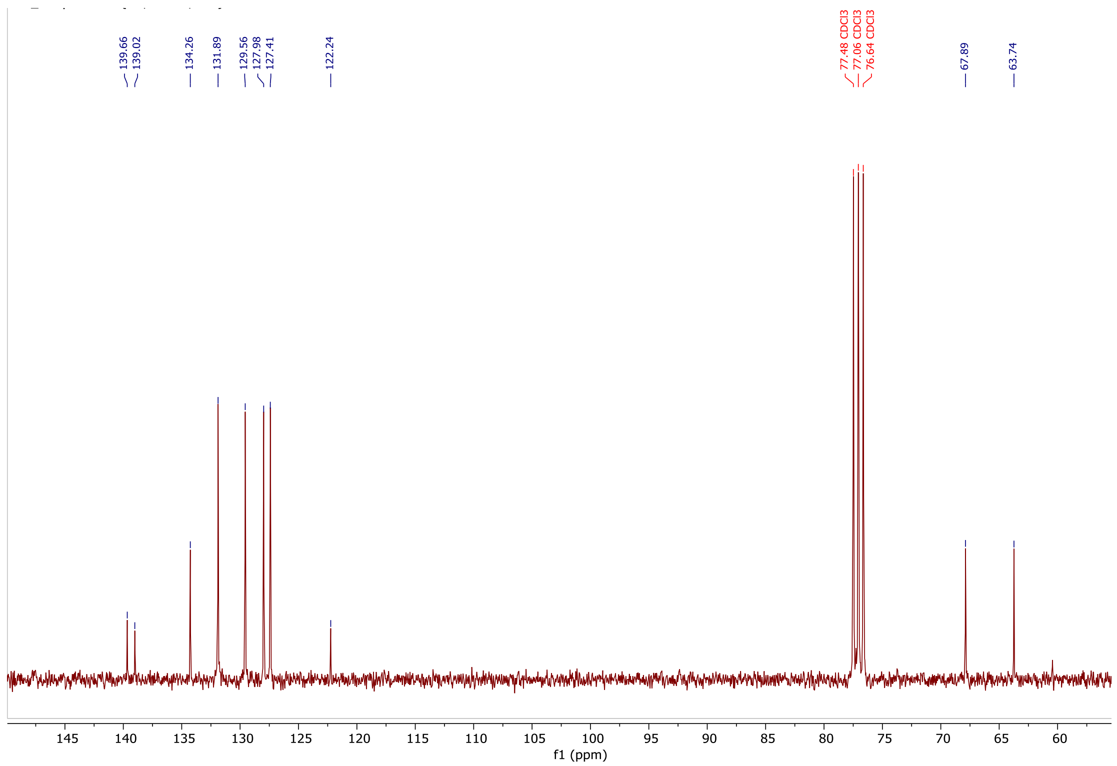
139.66
139.02
134.26
131.89
129.56
127.98
127.41
122.24
77.48 CDCl3
77.06 CDCl3
76.64 CDCl3
67.89
63.74
145 140 135 130 125 120 115 110 105 100 95 90 85 80 75 70 65 60
f1 (ppm)

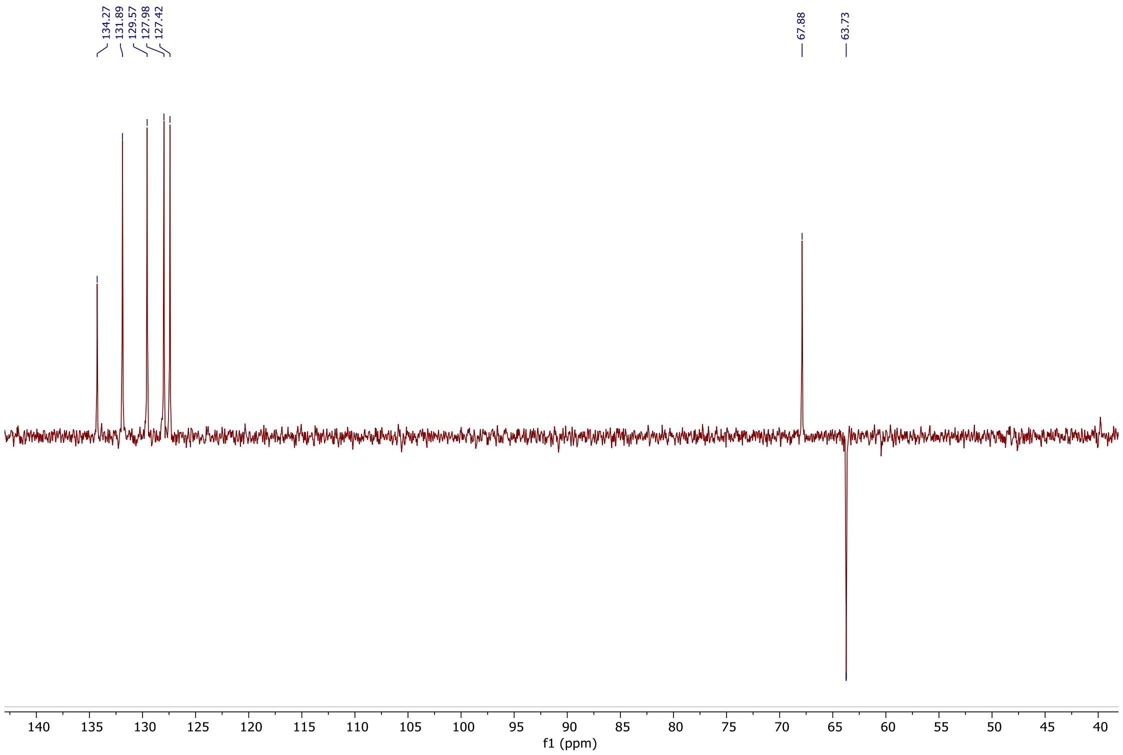
134.27
131.89
129.57
127.98
127.42
67.88
63.73
140 135 130 125 120 115 110 105 100 95 90 85 80 75 70 65 60 55 50 45 40
f1 (ppm)

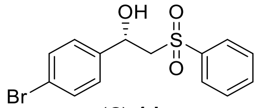

(*S*)-**4d**

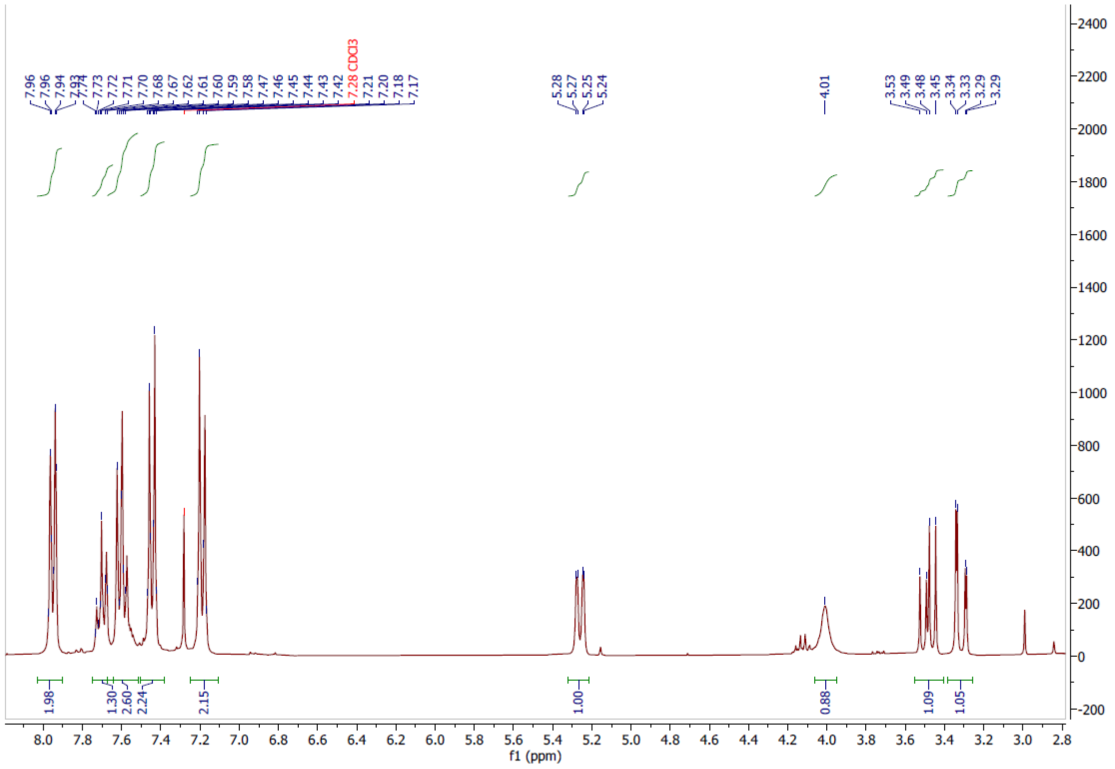

7.96
7.96
7.94
7.93
7.74
7.73
7.72
7.71
7.70
7.68
7.67
7.62
7.61
7.60
7.59
7.58
7.47
7.46
7.45
7.44
7.43
7.42
7.28 CDCl3
7.21
7.20
7.18
7.17
5.28
5.27
5.25
5.24
4.01
3.53
3.49
3.48
3.45
3.34
3.33
3.29
3.29
1.98
1.30
2.60
2.24
2.15
1.00
0.88
1.09
1.05
f1 (ppm)

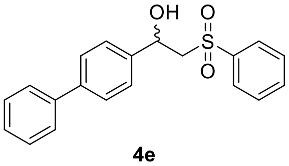

4e

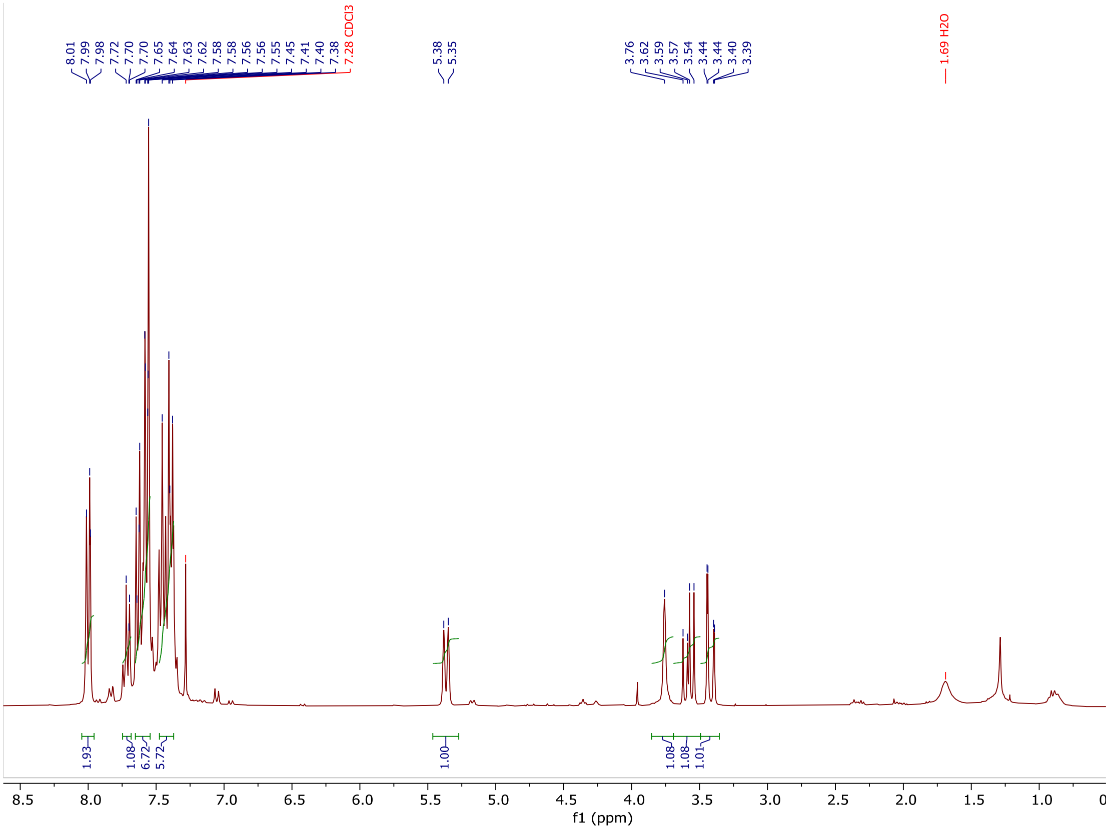

8.01
7.99
7.98
7.72
7.70
7.70
7.65
7.64
7.63
7.62
7.58
7.58
7.56
7.56
7.55
7.45
7.41
7.40
7.38
7.28 CDCl3
5.38
5.35
3.76
3.62
3.59
3.57
3.54
3.44
3.44
3.40
3.39
1.69 H2O
1.93
1.08
6.72
5.72
1.00
1.08
1.08
1.01
f1 (ppm)

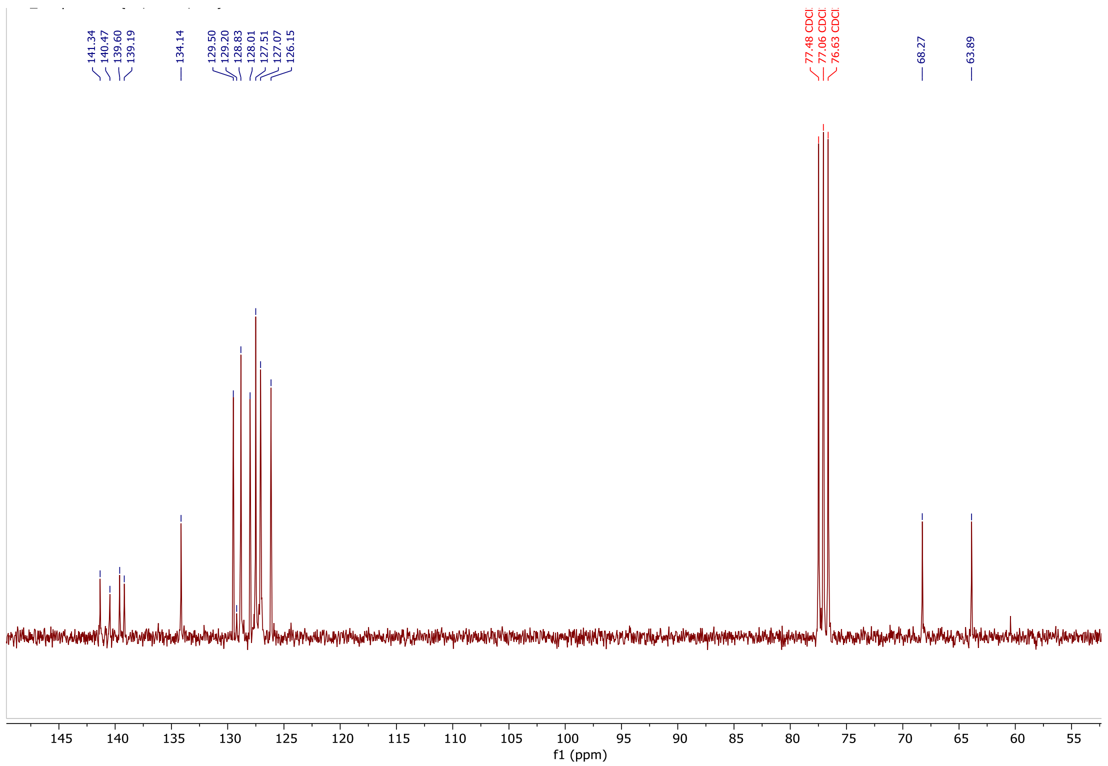
141.34
140.47
139.60
139.19
134.14
129.50
129.20
128.83
128.01
127.51
127.07
126.15
77.48 CDCl
77.06 CDCl
76.63 CDCl
68.27
63.89
145 140 135 130 125 120 115 110 105 100 95 90 85 80 75 70 65 60 55
f1 (ppm)

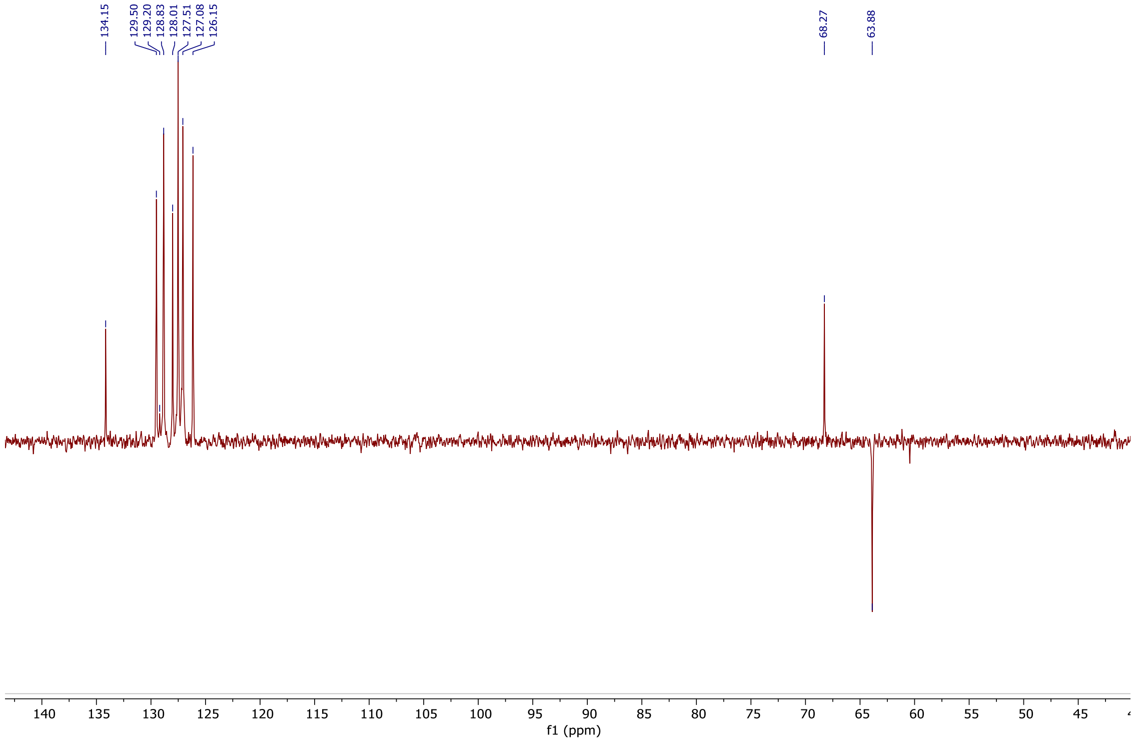
134.15
129.50
129.20
128.83
128.01
127.51
127.08
126.15
68.27
63.88
140 135 130 125 120 115 110 105 100 95 90 85 80 75 70 65 60 55 50 45
f1 (ppm)

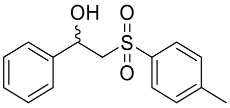

4f

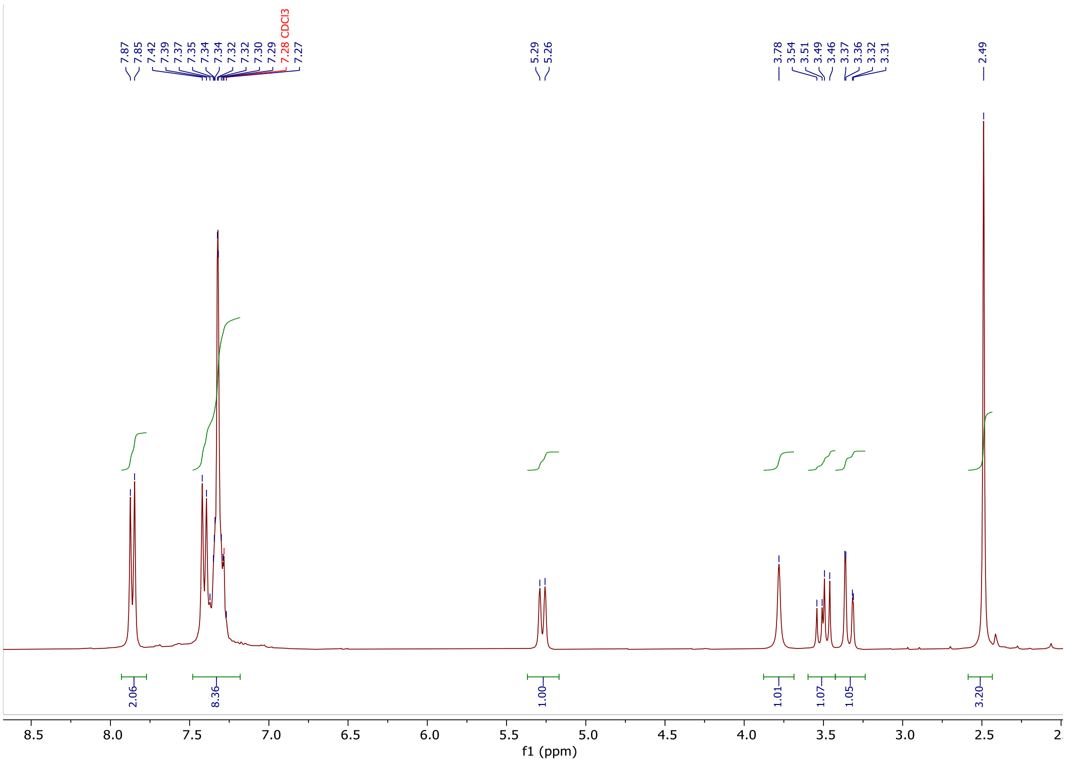

7.87
7.85
7.42
7.39
7.37
7.35
7.34
7.34
7.32
7.30
7.29
7.28 CDCl3
7.27
5.29
5.26
3.78
3.54
3.51
3.49
3.46
3.37
3.36
3.32
3.31
2.49
2.06
8.36
1.00
1.01
1.07
1.05
3.20
8.5
8.0
7.5
7.0
6.5
6.0
5.5
5.0
4.5
4.0
3.5
3.0
2.5
2
f1 (ppm)

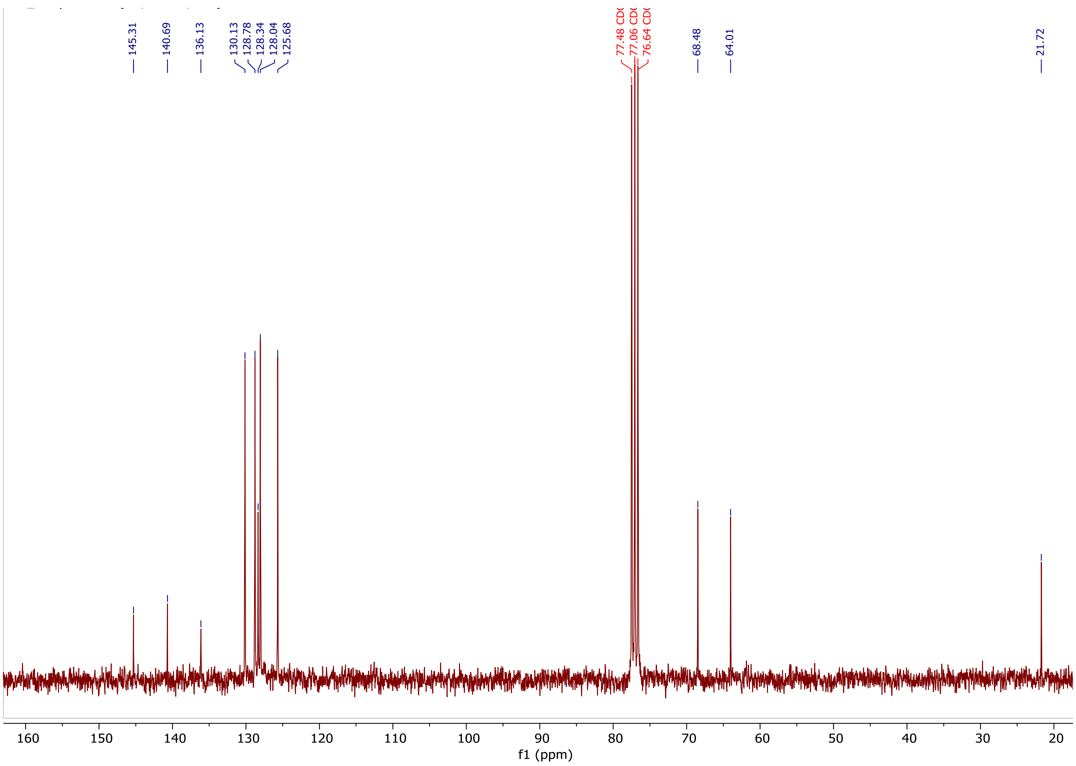
145.31
140.69
136.13
130.13
128.78
128.34
128.04
125.68
77.48 CDCl3
77.06 CDCl3
76.64 CDCl3
68.48
64.01
21.72
160
150
140
130
120
110
100
90
80
70
60
50
40
30
20
f1 (ppm)

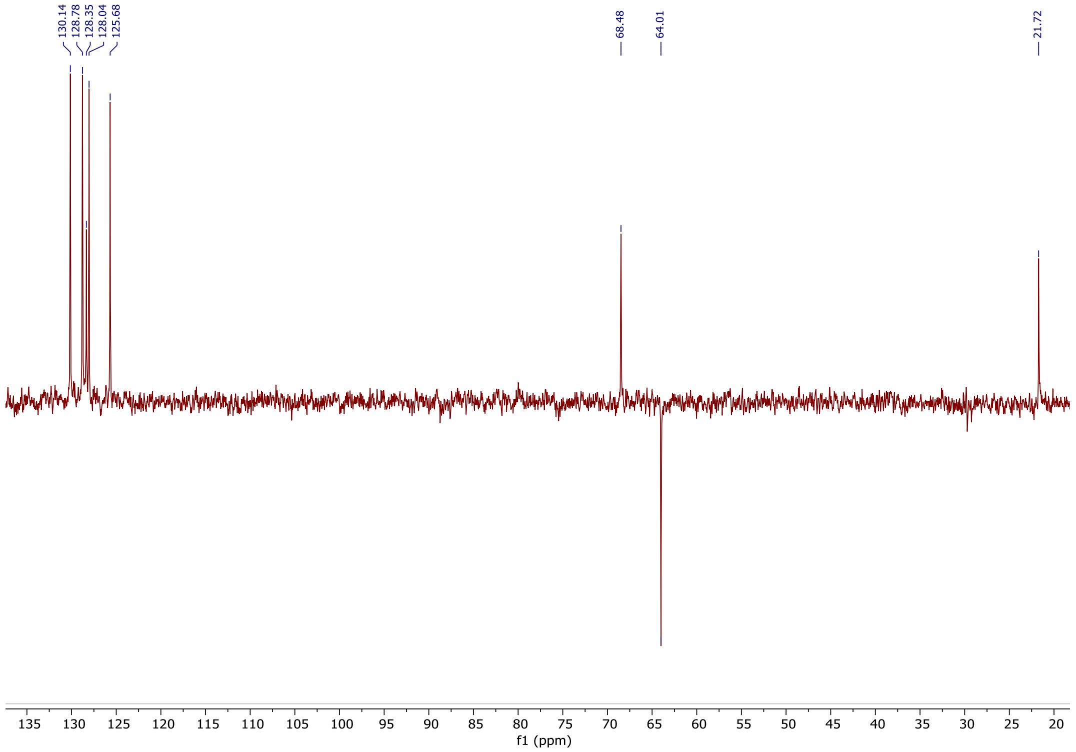
130.14
128.78
128.35
128.04
125.68
68.48
64.01
21.72
135
130
125
120
115
110
105
100
95
90
85
80
75
70
65
60
55
50
45
40
35
30
25
20
f1 (ppm)

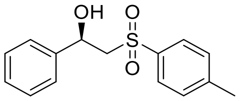

(*R*)-**4f**

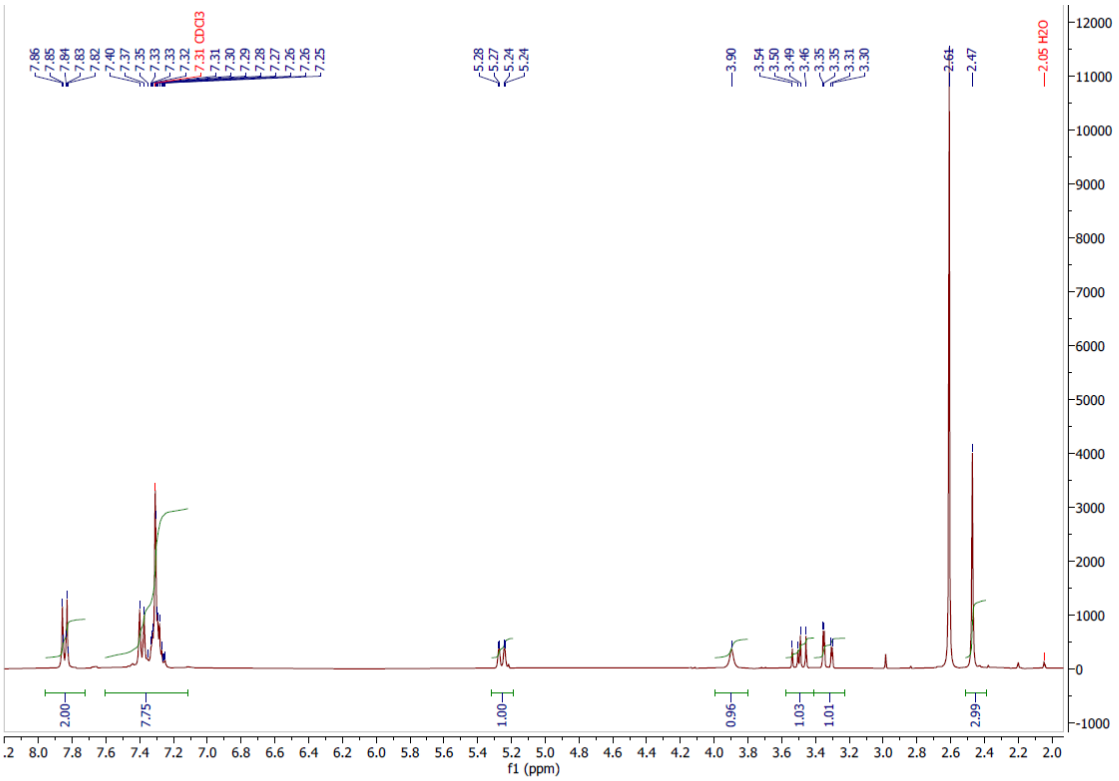
7.86
7.85
7.84
7.83
7.82
7.40
7.37
7.35
7.33
7.33
7.32
7.31 CDCl3
7.31
7.30
7.29
7.28
7.27
7.26
7.26
7.25
5.28
5.27
5.24
5.24
3.90
3.54
3.50
3.49
3.46
3.35
3.35
3.31
3.30
2.61
2.47
2.05 H2O
2.00
7.75
1.00
0.96
1.03
1.01
2.99
f1 (ppm)

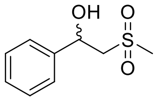

4g

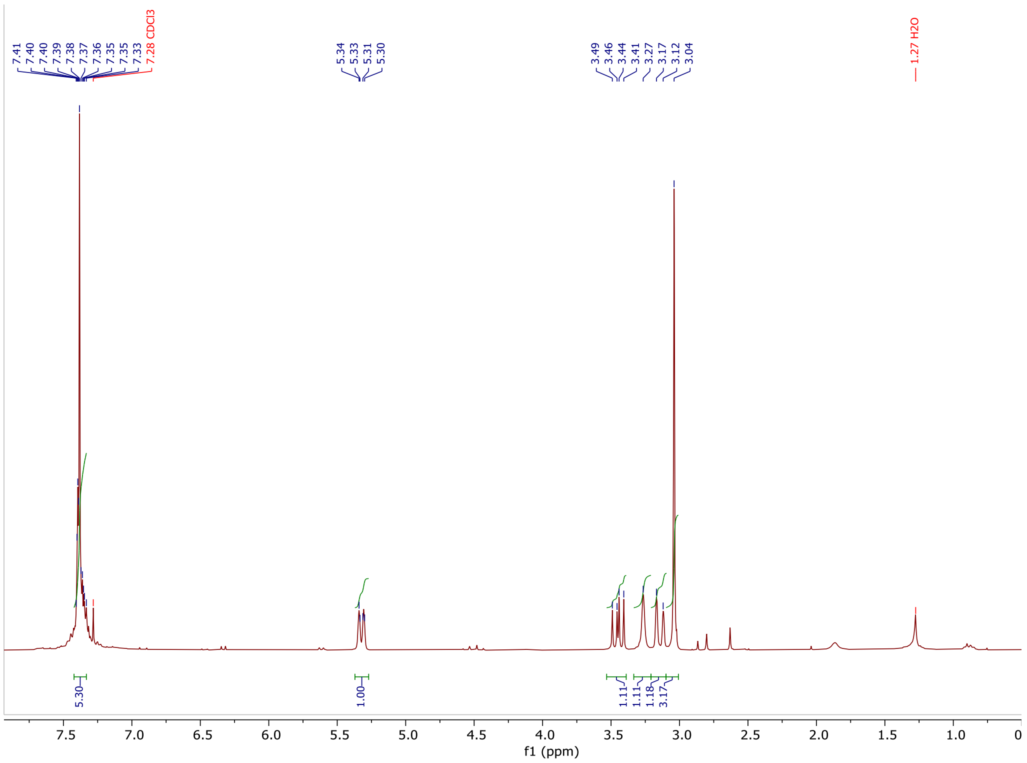

7.41
7.40
7.40
7.39
7.38
7.37
7.36
7.35
7.35
7.33
7.28 CDCl3
5.34
5.33
5.31
5.30
3.49
3.46
3.44
3.41
3.27
3.17
3.12
3.04
1.27 H2O
5.30
1.00
1.11
1.11
1.18
3.17
7.5
7.0
6.5
6.0
5.5
5.0
4.5
4.0
3.5
3.0
2.5
2.0
1.5
1.0
0
f1 (ppm)

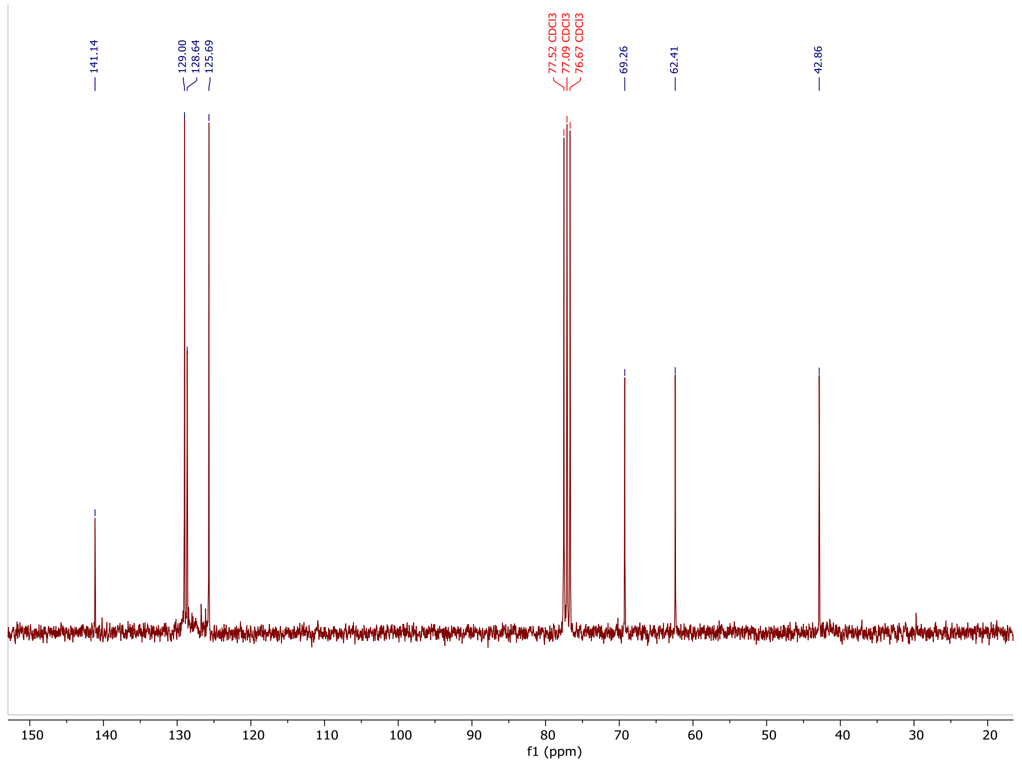
141.14
129.00
128.64
125.69
77.52 CDCl3
77.09 CDCl3
76.67 CDCl3
69.26
62.41
42.86
150 140 130 120 110 100 90 80 70 60 50 40 30 20
f1 (ppm)

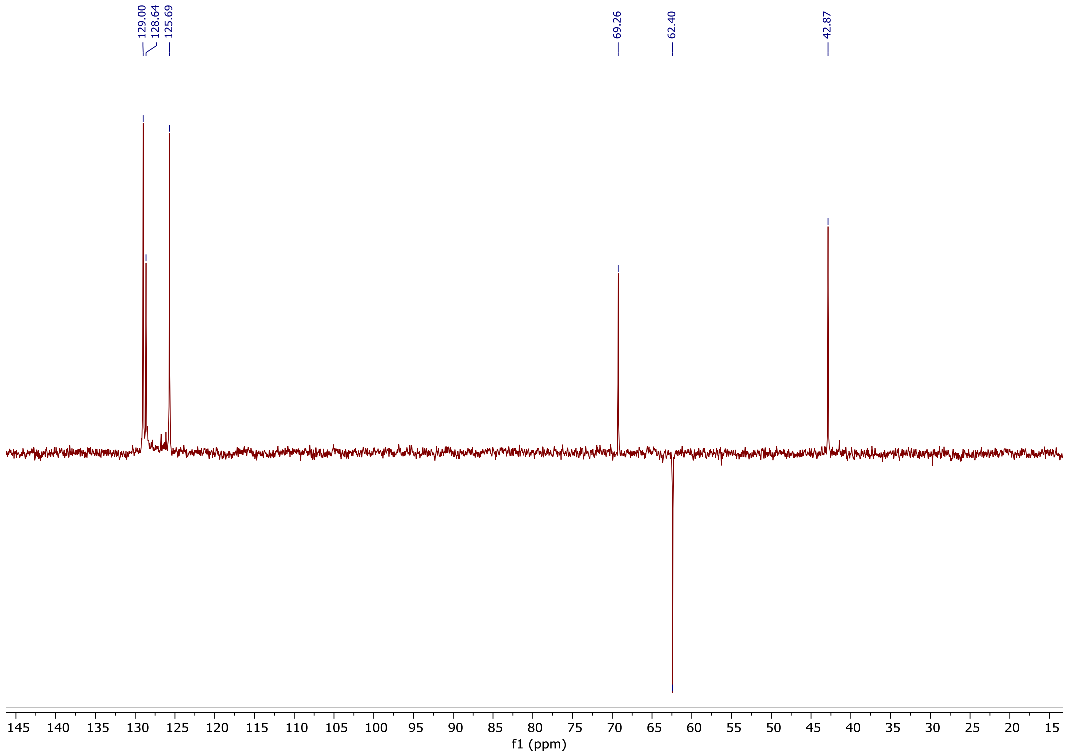
129.00
128.64
125.69
69.26
62.40
42.87
145 140 135 130 125 120 115 110 105 100 95 90 85 80 75 70 65 60 55 50 45 40 35 30 25 20 15
f1 (ppm)

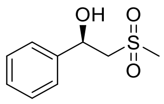
OH
O
S
O

(*R*)-**4g**

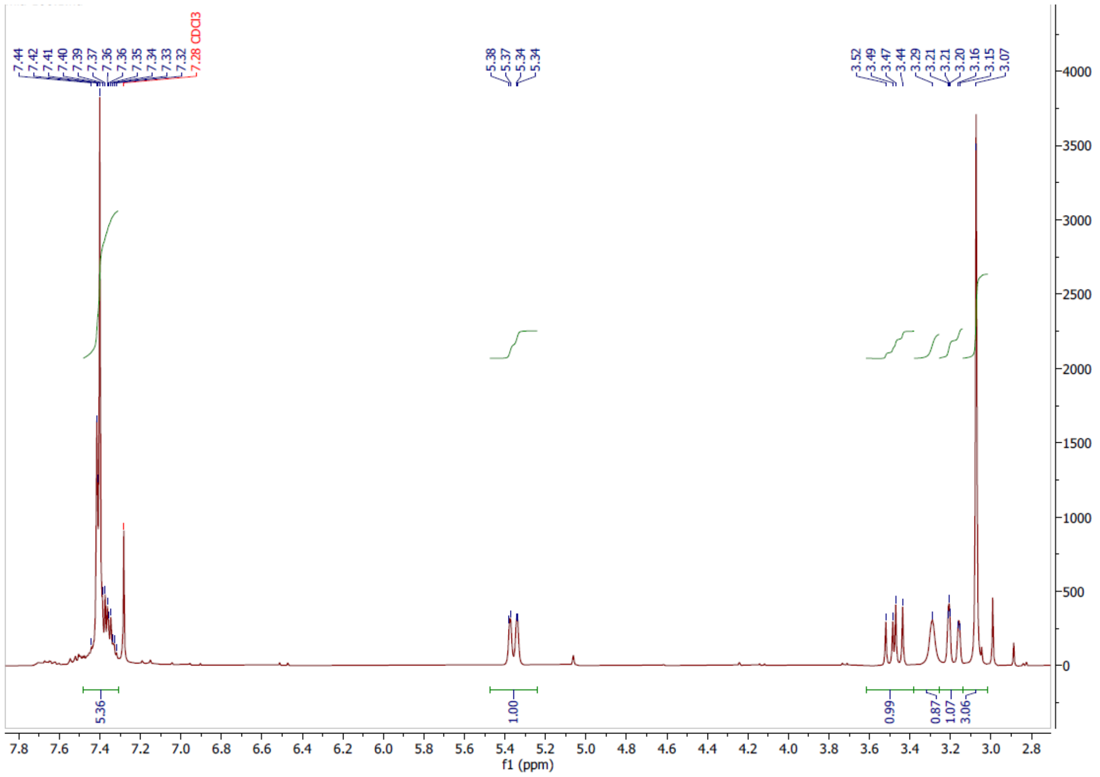
7.44
7.42
7.41
7.40
7.39
7.37
7.36
7.36
7.35
7.34
7.33
7.32
7.28 CDCl3
5.38
5.37
5.34
5.34
3.52
3.49
3.47
3.44
3.29
3.21
3.21
3.20
3.16
3.15
3.07
5.36
1.00
0.99
0.87
1.07
3.06
f1 (ppm)

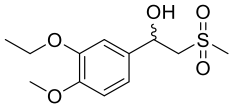

4h

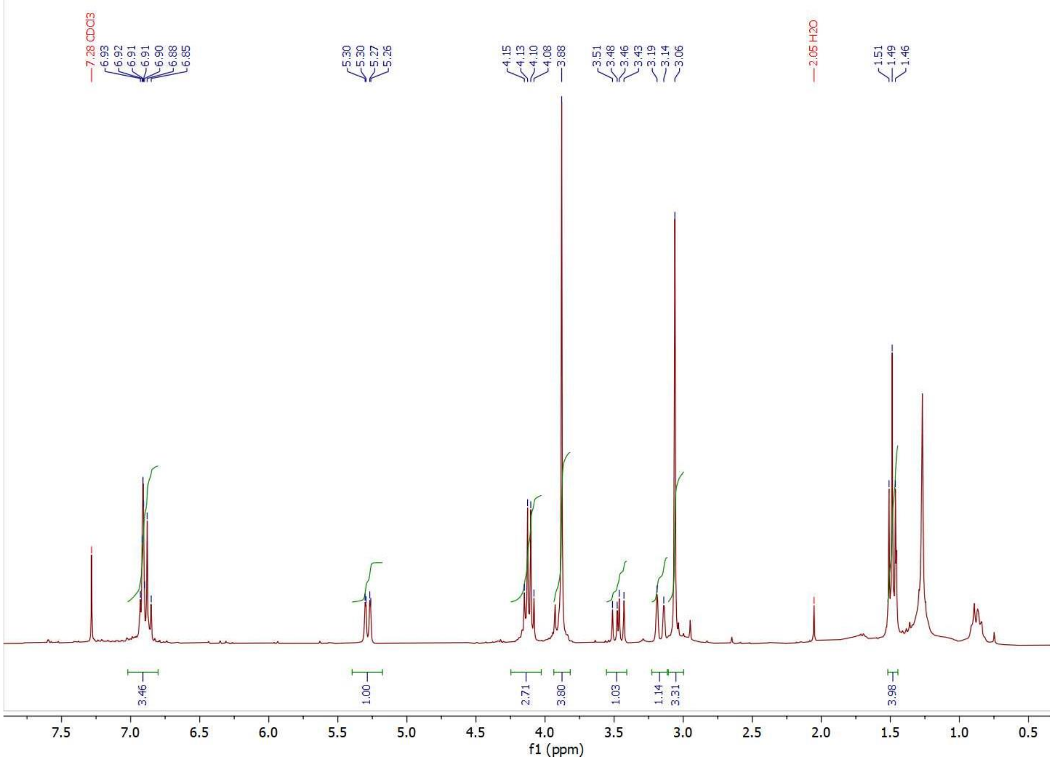
7.28 CDCl3
6.93
6.92
6.91
6.91
6.90
6.88
6.85
5.30
5.30
5.27
5.26
4.15
4.13
4.10
4.08
3.88
3.51
3.48
3.46
3.43
3.19
3.14
3.06
2.05 H2O
1.51
1.49
1.46
3.46
1.00
2.71
3.80
1.03
1.14
3.31
3.98
7.5
7.0
6.5
6.0
5.5
5.0
4.5
4.0
3.5
3.0
2.5
2.0
1.5
1.0
0.5
f1 (ppm)

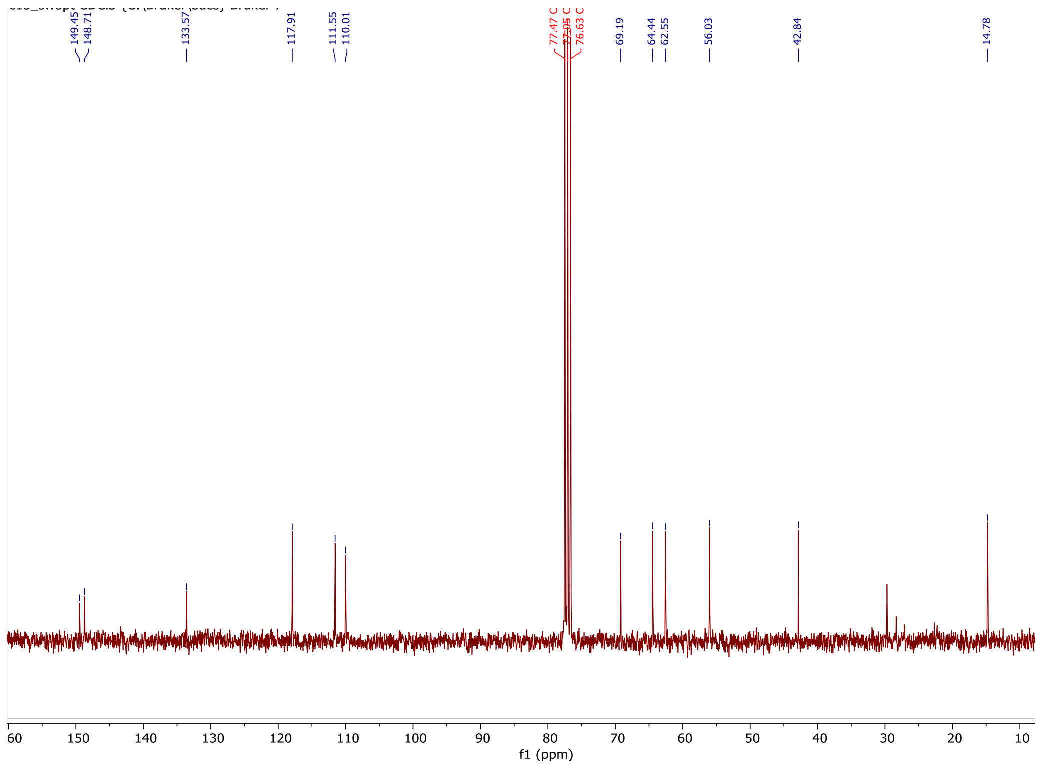
149.45
148.71
133.57
117.91
111.55
110.01
77.47 C
77.05 C
76.63 C
69.19
64.44
62.55
56.03
42.84
14.78
f1 (ppm)

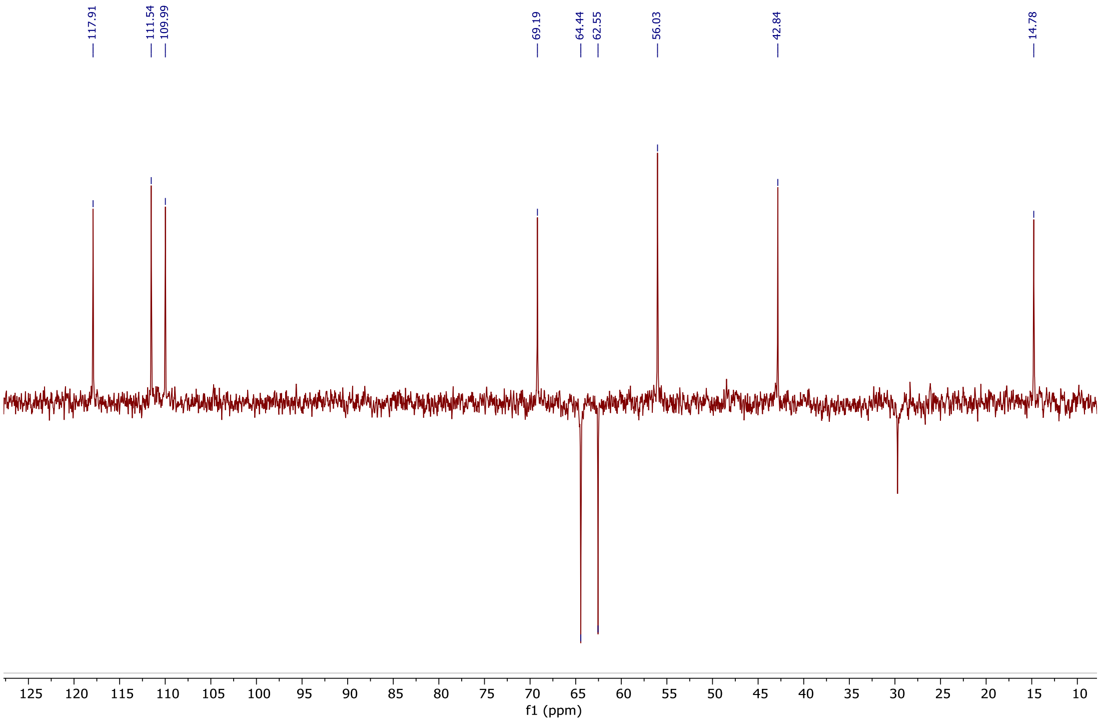
117.91
111.54
109.99
69.19
64.44
62.55
56.03
42.84
14.78
f1 (ppm)
